# OsChz1 acts as a histone chaperone in modulating chromatin organization and genome function in rice

**DOI:** 10.1038/s41467-020-19586-z

**Published:** 2020-11-11

**Authors:** Kangxi Du, Qiang Luo, Liufan Yin, Jiabing Wu, Yuhao Liu, Jianhua Gan, Aiwu Dong, Wen-Hui Shen

**Affiliations:** 1 State Key Laboratory of Genetic Engineering, Collaborative Innovation Center of Genetics and Development, International Associated Laboratory of CNRS-Fudan-HUNAU on Plant Epigenome Research, Department of Biochemistry, Institute of Plant Biology, School of Life Sciences, Fudan University, Shanghai, 200438 China; 2 Department of Physiology and Biophysics, School of Life Sciences, Fudan University, Shanghai, 200438 China; 3 Institut de Biologie Moléculaire des Plantes, UPR2357 CNRS, Université de Strasbourg, 12 rue du Général Zimmer, 67084 Strasbourg, Cédex France

**Keywords:** Epigenetics, Gene regulation, Plant genetics

## Abstract

While the yeast Chz1 acts as a specific histone-chaperone for H2A.Z, functions of CHZ-domain proteins in multicellular eukaryotes remain obscure. Here, we report on the functional characterization of OsChz1, a sole CHZ-domain protein identified in rice. OsChz1 interacts with both the canonical H2A-H2B dimer and the variant H2A.Z-H2B dimer. Within crystal structure the C-terminal region of OsChz1 binds H2A-H2B via an acidic region, pointing to a previously unknown recognition mechanism. Knockout of *OsChz1* leads to multiple plant developmental defects. At genome-wide level, loss of OsChz1 causes mis-regulations of thousands of genes and broad alterations of nucleosome occupancy as well as reductions of H2A.Z-enrichment. While OsChz1 associates with chromatin regions enriched of repressive histone marks (H3K27me3 and H3K4me2), its loss does not affect the genome landscape of DNA methylation. Taken together, it is emerging that OsChz1 functions as an important H2A/H2A.Z-H2B chaperone in dynamic regulation of chromatin for higher eukaryote development.

## Abstract (web summary)

Function of CHZ-domain proteins in multicellular eukaryotes remains unclear. Here, the authors characterize the sole CHZ-domain protein identified in rice and show that it functions as an H2A/H2A.Z-H2B chaperone in dynamic regulation of chromatin organization and genome function.

## Introduction

Nucleosome is the fundamental structural unit of chromatin in eukaryotes. It is composed of almost two superhelical turns of DNA (∼146 bp) wrapped around an octamer formed by two copies each of the four core histone proteins H2A, H2B, H3, and H4^1^. Linker histone H1 binds internucleosomal DNA to stabilize adjacent nucleosomes and to mediate higher-order chromatin packaging^2^. While chromatin appears rigid at the cytological level, nucleosomes are highly dynamic^3^. Nucleosome assembly, disassembly, and reassembly occur not only during genome replication but also during transcription and during DNA-damage repair^4^. Proper assembly of histones with DNA requires histone–chaperones, which function in preventing unintended interactions between the positive-electrical-charged histone and the negative-electrical-charged DNA molecules under physiological conditions^5,6^. In addition to the canonical histone dynamics, histone variant incorporation constitutes a key mean in modulating nucleosome compositions^7^.

Unlike the canonical histones that are packaged into nucleosomes majorly during DNA replication, histone variants are deposited throughout the cell cycle and are incorporated into nucleosomes largely in DNA replication-independent pathways^8^. Among the five families of histones, the H2A family possesses the largest number of variants, including H2A.Z, H2A.X, macroH2A, H2A.Bbd, and H2A.W^9,10^. From them, H2A.Z is most highly conserved across different eukaryote kingdoms during evolution^11,12^. In yeast, animal, and plant, H2A.Z is found at many genes and is distributed most often around transcription start sites (TSS) with a particular preference for +1 nucleosome^13–17^. H2A.Z accumulation can affect transcription positively or negatively. Studies in plants, majorly in Arabidopsis (*Arabidopsis thaliana*) but also in rice (*Oryza sativa*), indicate that the presence of H2A.Z at TSS associates with transcriptional activation or repression, whereas its presence over gene bodies negatively correlates with transcription^18–24^. Besides transcription, H2A.Z is also involved in the regulation of other genomic processes, such as anti-silencing and anti-spreading of heterochromatin, chromosome segregation, homologous recombination, and DNA repair^11^. Strikingly, in contrast to the extensively studied functions of H2A.Z, histone–chaperones responsible for escorting H2A.Z in nucleosome assembly/disassembly remain poorly known.

NUCLEOSOME ASSEMBLY PROTEIN1 (NAP1) is evolutionarily conserved and belongs to the H2A-H2B histone–chaperone family^25,26^. In addition to its major function as chaperone of the canonical H2A-H2B histones, the yeast NAP1 (yNAP1) was found in multiprotein complexes co-immunopurified with H2A.Z^27^. Either free or yNAP1-associated H2A.Z-H2B dimers could be transferred by the ATP-dependent chromatin-remodeling complex SWR1 (SWR1-c) to replace H2A-H2B in an in vitro nucleosome assembly assay, implying that yNAP1 functions as an escort rather than part of the histone transfer machinery^27^. Later, Chz1 (Chaperone for H2A.Z-H2B) was identified as a specific chaperone for H2A.Z in yeast^28^. An in vitro binding assay revealed that yChz1 exhibits a stronger interaction with the H2A.Z-H2B dimer than with the H2A-H2B dimer^28^. Structurally, yChz1 forms a long irregular chain capped by two short α-helix motifs when it binds to H2A.Z-H2B, and the binding of yChz1 to H2A.Z-H2B enhances the stability of the yChz1-H2A.Z-H2B complex^28,29^. yChz1 binds at a specific surface of H2A.Z, which differs from the yNAP1-binding surface, and yChz1 and yNAP1 exhibit both redundant and distinct functions in the subcellular transport and the chromatin incorporation of H2A.Z^30–32^. More recently, a DEF/Y motif located at the C-terminus of yChz1 was identified to directly engage the arginine residues within the H2A.Z L2 loop and a hydrophobic pocket in H2B for H2A.Z-H2B dimer interaction, enhancing the binding preference of yChz1 for H2A.Z-H2B^33^. Surprisingly, instead of working in H2A.Z incorporation, the Arabidopsis NAP1-RELATED PROTEIN1 (NRP1) and NRP2 were proposed to regulate gene expression by counteracting SWR1 to prevent excessive accumulation of H2A.Z in chromatin^34^.

In this work, we characterize *OsChz1*, a sole gene encoding CHZ-domain protein in the agronomical important crop rice to gain knowledge about the function of CHZ-domain proteins in higher eukaryotes. Molecular and biochemical analyses reveal that *OsChz1* is broadly expressed in various tissues of rice and the OsChz1 protein binds both H2A-H2B and H2A.Z-H2B. The crystal structure of OsChz1-H2A-H2B complex shows that OsChz1 uses an acidic binding surface to engage the H2A-H2B dimer. CRISPR/Cas9-knockout of *OsChz1* uncovers its key functions in the regulation of multiple plant growth and developmental processes. Genome-wide profiling at transcriptome, nucleosome occupancy, H2A.Z enrichment, OsChz1 binding, and DNA-methylation levels demonstrate that *OsChz1* plays key functions in vivo in the regulation of chromatin dynamics and genome transcription.

## Results

### Identification and expression analysis of *OsChz1*

**Fig. 1:**
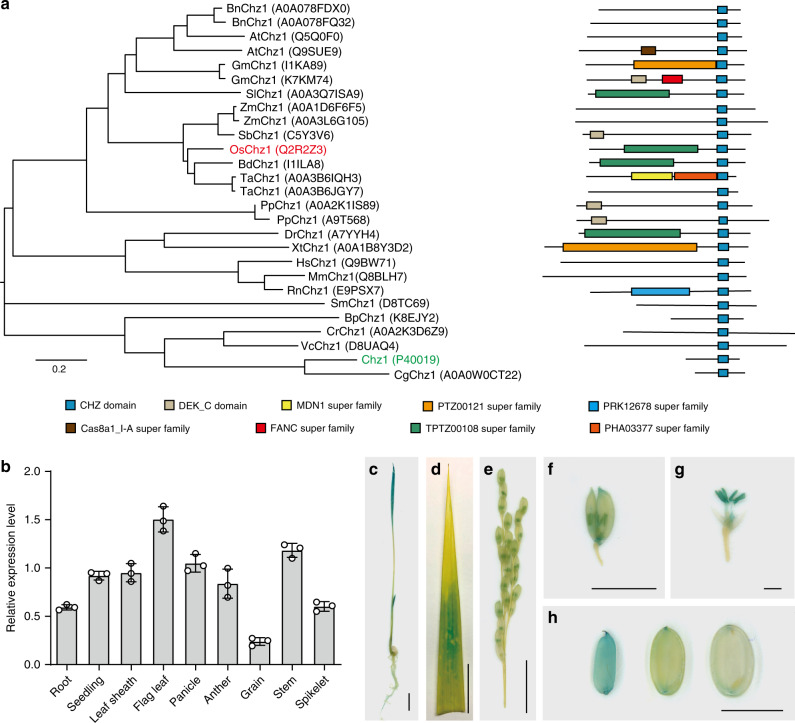
Identification and expression analysis of *OsChz1*. **a** Phylogenetic tree (left) and protein domain organization (right) analyses of CHZ-domain-containing proteins in higher eukaryotes. The founding member from yeast (yChz1) was highlighted in green; and the Chz1 homolog identified in rice, OsChz1, was highlighted in red. Neighbor-Joining phylogeny based on full-length amino acid sequence alignment was calculated using MEGA v10.1.8 and illustrated using FigTree v1.4.3. The diverse protein domains are displayed in different colored boxes as indicated. Bn, *Brassica napus*; At, *Arabidopsis thaliana*; Sl, *Solanum lycopersicum*; Gm, *Glycine max*; Ta, *Triticum aestivum*; Bd, *Brachypodium distachyon*; Os, *Oryza sativa*; Sb, *Sorghum bicolor*; Zm, *Zea mays*; Pp, *Physcomitrella patens*; Xt, *Xenopus tropicalis*; Dr, *Danio rerio*; Hs, *Homo sapien*s; Mm, *Mus musculus*; Rn, *Rattus norvegicus*; Bp, *Bathycoccus prasinos*; Sm, *Selaginella moellendorffii*; Cr, *Chlamydomonas reinhardtii*; Vc, *Volvox carteri*; Cg, *Candida glabrata*. **b** Quantitative RT-PCR analysis of *OsChz1* expression. Relative expression levels are shown for root (10-day-old), seedling (10-day-old), leaf sheath, flag leaf, panicle, anther, grain (3 days after anthesis), stem, and spikelet. The rice *Ubiquitin5* gene was used as an internal control. Values are means ± standard deviation from *n* = 3 independent biological replicates. **c**–**h** Histochemical detection of *pOsChz1:GUS* expression in transgenic rice plants. GUS activity (staining in blue) was observed in seedling (**c**), flag leaf (**d**), panicle (**e**), spikelet (**f**), anther (**g**), and grains (**h**). Scale bars, 1 cm in **c**–**e**, 5 mm in **f**, **h**, 1 mm in **g**. Source data underlying **b** are provided as a Source Data file.

Using the CHZ-domain sequence in Blast search, we have identified yChz1 homologs in diverse organisms from the UniProt database. While absent in some invertebrates such as *Caenorhabditis elegans* and *Drosophila melanogaster*, CHZ-domain proteins are found in humans and vertebrates (Fig. 1a). Remarkably, CHZ-domain proteins are well-conserved during the green lineage evolution and are found in organisms ranging from unicellular algae to vasculature higher plants (Fig. 1a). Besides the CHZ domain, which is located near to the C-terminus, the other regions of the CHZ-domain proteins share little homology, with some potential functional domains identified but failed to show a substantial association with CHZ-domain during evolution (Fig. 1a). Unlike most higher plants where two or more copies of CHZ-encoding genes exist in a given species, rice contains a single gene, *OsChz1*, which encodes the sole CHZ-domain protein in this organism. RT-PCR analysis revealed that *OsChz1* is broadly expressed in various rice organs, with the highest level in flag leaf, varied intermediate levels in stem, panicle, leaf sheath, young seedling, anther, spikelet, and root, and the lowest level in mature grain (Fig. 1b). We further examined the expression pattern of *OsChz1* by promoter-fusion with the encoding sequence of the visual marker β-glucuronidase (*pOsChz1:GUS*). The *pOsChz1:GUS* transgenic plants showed strong GUS signals in the root, coleoptile, and leaf blade of young seedlings (Fig. 1c), in the basal part of flag leaves (Fig. 1d), in the spikelets of panicle (Fig. 1e, f), particularly in the male and female reproductive organs such as stamens and pistil (Fig. 1g), and in grains (Fig. 1h). The GUS signal in grains dropped drastically upon grain maturation and desiccation (Fig. 1g). Taken together, the expression pattern of *OsChz1* indicates that it could play important functions during rice plant growth and development, and the single copy of this gene provides a facility in its knockout for functional study in planta.

### OsChz1 can act as a histone–chaperone for both H2A-H2B and H2A.Z-H2B

**Fig. 2:**
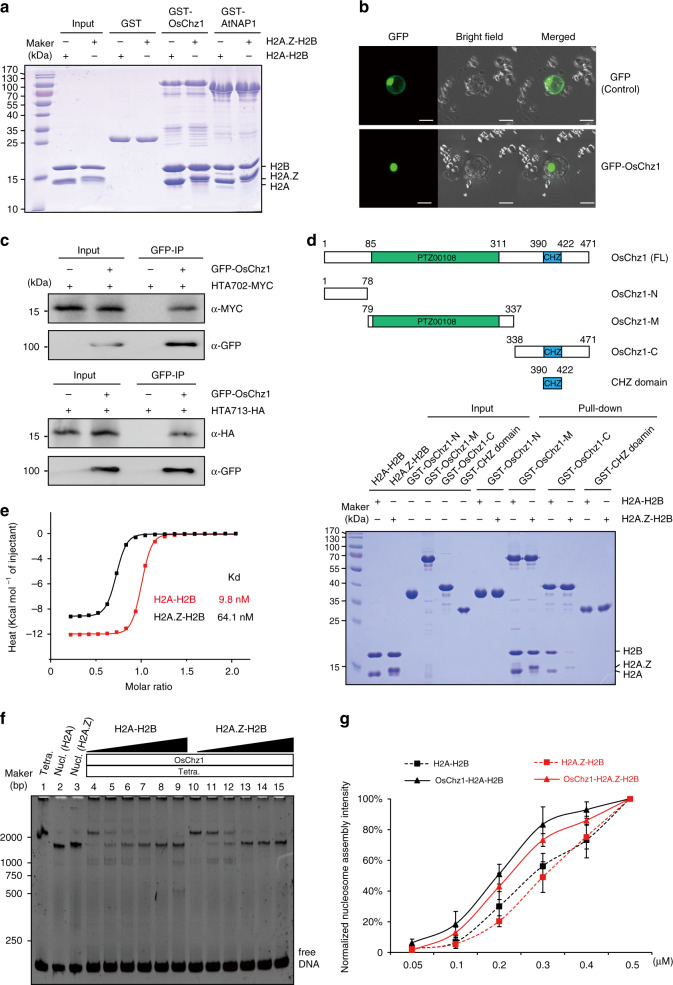
OsChz1 physically interacts with H2A-H2B and H2A.Z-H2B. **a** GST pull-down assay showing interaction of OsChz1 with H2A-H2B and H2A.Z-H2B. Beads coated with GST, GST-OsChz1 or GST-AtNAP1 were used to pull down H2A-H2B or H2A.Z-H2B as indicated (+, presence; −, absence). Image shows gel with Coomassie-blue-staining. **b** Localization of the Green Fluorescent Protein (GFP)-OsChz1 fusion protein in rice protoplast. Scale bar, 10 μm. **c** Co-immunoprecipitation (Co-IP) assay showing the interaction of OsChz1 with HTA702 (H2A) and HTA713 (H2A.Z) in rice protoplast. Protein extracts from transgenic rice protoplasts were immunoprecipitated with anti-GFP affinity beads and the resulting products were analyzed by western blot using the antibodies against GFP, MYC, or HA. **d** Examination of various regions of OsChz1 in binding with H2A-H2B and H2A.Z-H2B in pull-down assay. Top diagrams illustrate different segments of OsChz1: OsChz1-N, N-terminal region; OsChz1-M, middle region; OsChz1-C, C-terminal region; FL, full length. Positions of PTZ00108 (green) and CHZ domain (blue) are indicated. Image shows Coomassie-blue staining gel. **e** ITC (Isothermal Titration Calorimetry) binding curves of H2A-H2B (red) and H2A.Z-H2B (black) titrated by OsChz1-C. **f** Affinity shift assay showing detection of OsChz1 activity in nucleosome assemble. Image shows a native PAGE gel. Lanes 1–3: H3-H4-DNA tetrasomes (tetra.), H2A- and H2A.Z-containing nucleosomes (nucl.); lanes 4–9: reactions of increasing amounts of H2A-H2B with a mixture of H3-H4-DNA tetrasome and OsChz1; lanes 10–15, reactions of increasing amounts of H2A.Z-H2B with the same mixture of H3-H4-DNA tetrasome and OsChz1. **g** Line-chart showing intensity quantifications of nucleosome assembly. The *x*-axis indicates the increasing amounts of H2A-H2B or H2A.Z-H2B. Black and red curves represent H2A-H2B and H2A.Z-H2B depositions, respectively. Solid lines represent histone deposition with OsChz1 (at 0.1 μM) and dashed lines represent histone deposition without OsChz1. Highest values, e.g., lane 9 for H2A-H2B and lane 15 for H2A.Z-H2B in **f**, were set as 100%. The data shown are means ± standard deviation from *n* = 3 independent biological replicates. Experiments were repeated independently three times (**a**, **b**, **f**) or twice (**c**, **e**) with similar results. Source data underlying **a** and **c**–**g** are provided as a Source Data file.

To examine whether or not OsChz1 physically interacts with histone dimers H2A-H2B and H2A.Z-H2B in vitro, a glutathione S-transferase (GST) pull-down assay was performed using a recombinant N-terminal GST-tagged OsChz1 full-length protein (GST-OsChz1). The Arabidopsis histone H2A (HTA1) shares high sequence homologies with the rice H2A (HTA702), and also the Arabidopsis histone variant H2A.Z (HTA9) shares high sequence homologies with the rice H2A.Z (HTA713) (Supplementary Fig. 1a). Thus, we have used in our pull-down assays the Arabidopsis H2A-H2B dimer, which had been used as recombinant proteins in a previous study^35^, and the Arabidopsis H2A.Z-H2B dimer produced similarly. We observed that both the H2A-H2B and the H2A.Z-H2B dimers could be pulled down by GST-OsChz1, but not by GST alone (Fig. 2a). To investigate the subcellular localization of OsChz1, we designed an enhanced green fluorescent protein (GFP)-OsChz1 fusion construct driven by the maize (*Zea mays*) ubiquintin-1 (Ubi) promoter. Transient expression of this construct in rice protoplast and fluorescence microscopy analyses revealed that GFP-OsChz1 was located in the cell nucleus (Fig. 2b). We further co-expressed GFP-OsChz1 together with a MYC-tagged rice histone H2A protein (HTA702-MYC) or a HA-tagged rice histone variant H2A.Z protein (HTA713-HA) in rice protoplasts, and performed co-immunoprecipitation (Co-IP) assays. Consistent with the results of our pull-down analysis, it was observed that both HTA702-MYC and HTA713-HA could be co-immunoprecipitated by GFP-OsChz1 (Fig. 2c).

To gain information about regions of OsChz1 involved in histone binding, we divided OsChz1 into four segments: an N-terminal region (OsChz1 1–78, OsChz1-N), a middle region (OsChz1 79–337, OsChz1-M) containing weak homologies with some DNA-topoisomerase2-like proteins forming the PTZ00180 superfamily (Supplementary Fig. 2), a C-terminal region (OsChz1 338–471, OsChz1-C) containing the evolutionarily conserved CHZ domain (Supplementary Fig. 3), and the CHZ-domain region (OsChz1 390–422). Our in vitro pull-down assays showed that both OsChz1-M and OsChz1-C but not OsChz1-N nor CHZ-domain exhibit binding activities to either H2A-H2B or H2A.Z-H2B (Fig. 2d). Visually, OsChz1-C seemed to pull down more efficiently H2A-H2B than H2A.Z-H2B. To verify this, we examined the binding of OsChz1-C to H2A-H2B and H2A.Z-H2B by isothermal titration calorimetry (ITC) experiments. We found that OsChz1-C achieves an ~6.5-fold higher affinity for H2A-H2B (Kd of 9.8 nM) than for H2A.Z-H2B (Kd of 64.1 nM) (Fig. 2e).

Next, we examined OsChz1 function in nucleosomal deposition of H2A-H2B and H2A.Z-H2B by using in vitro assays^36^. We assembled the (H3-H4)_2_-DNA tetrasome complexes as well as the H2A-containing and H2A.Z-containing nucleosomes, respectively (Supplementary Fig. 4a). In the absence of OsChz1, both the H2A-H2B and H2A.Z-H2B dimers were found readily capable to bind the (H3-H4)_2_-DNA tetrasome complexes to form nucleosomes (Supplementary Fig. 4b). Thus, we examined OsChz1 activity by comparing nucleosome formation efficiency under specified conditions. For this, the (H3-H4)_2_-DNA tetrasome complexes were incubated, in the presence of a fixed concentration of OsChz1, with increasing amounts of H2A-H2B or H2A.Z-H2B for 30 min (Fig. 2f). A concentration-dependent efficient nucleosome assembly was observed, and this occurred significantly more efficiently in the presence of OsChz1 than in the absence of OsChz1 for both H2A-H2B and H2A.Z-H2B (Fig. 2g). This observation is in line with a histone–chaperone activity of OsChz1 that facilitates nucleosome assembly. In this assay again OsChz1 did not show any preference for H2A.Z over H2A, and H2A-H2B displayed more efficient assembly with the (H3-H4)_2_-DNA tetrasome complex than did H2A.Z-H2B in either the presence or the absence of OsChz1 (Fig. 2g).

### Structural basis for H2A-H2B dimer recognition by the C-terminal region of OsChz1

The absence of OsChz1 preference for H2A.Z is in contrast to the preference of yChz1 for H2A.Z reported previously^28^. To understand the molecular basis of interaction between OsChz1 and H2A-H2B, we crystallized OsChz1-C in complex with H2A-H2B, and obtained the crystal structure of the complex at a 2.85 Å resolution by the molecular replacement (MR) method (Supplementary Table 1).

**Fig. 3:**
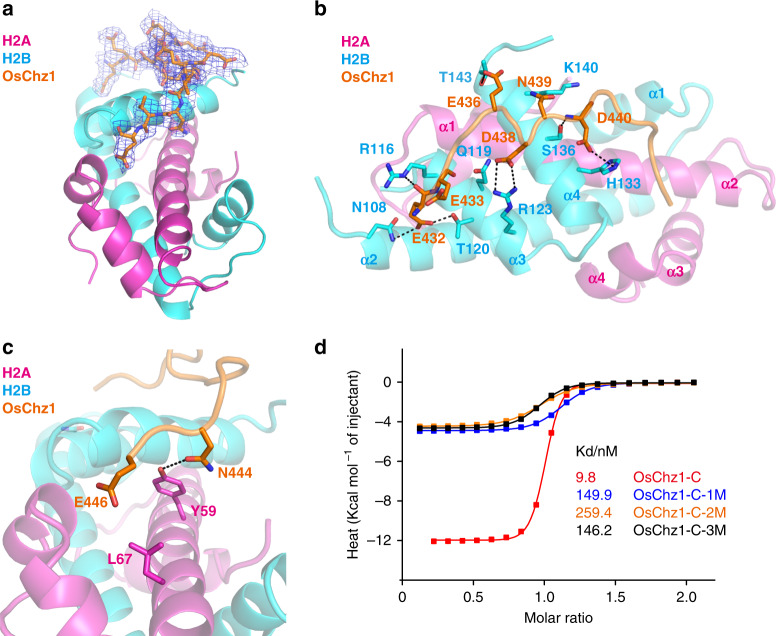
Crystal structure showing OsChz1-C binding with the H2A-H2B heterodimer. **a** Crystal structure of OsChz1-C complexed with H2A-H2B. H2A and H2B are shown as cartoons in purple and cyan, respectively. OsChz1-C is shown as sticks in atomic colors (C, orange; N, blue; O, red). The 2Fo-Fc omit map is contoured at the 0.5σ level. **b**, **c** Detailed interactions between OsChz1-C and H2B and between OsChz1 and H2A, respectively. Dash-lines indicate hydrogen bond (H-bond) interactions. **d** ITC (isothermal titration calorimetry) curves showing the impact of OsChz1-C mutations on binding with H2A-H2B. The wild-type OsChz1-C is shown in red color and its value is the same to that in Fig. 2e, and the mutants 1M, 2M, and 3M are in blue, orange, and black, respectively. The detailed sequences of these OsChz1 mutants are listed in Supplementary Table 2. Source data underlying **d** are provided as a Source Data file.

As supported by the 2Fo-Fc electron density map, residues 432–446aa of OsChz1 are well ordered in the structure (Fig. 3a). The N-terminal half of OsChz1-C forms extensive interactions with H2B, through residues located at the α3 and α4 helix regions (Fig. 3b). In detail, the main chain N atom of OsChz1 Glu432 is in contact with the side chain of H2B Asn108, while the main chain O atom of OsChz1 Glu432 directly interacts with the side chain of H2B Arg116. In addition, the carboxyl of OsChz1 Glu432 forms one hydrogen bond (H-bond) with the side chain of H2B Thr120. Next, the main chain O atom of OsChz1 Glu433 binds to the side chain of H2B Gln119. The side chain of OsChz1 Glu436 binds to H2B Thr143 via H-bond interaction, and OsChz1 Asp438 forms a salt bridge with H2B Arg123. OsChz1 Asn439 makes a contact with H2B Lys140. Furthermore, the main chain N atom of OsChz1 Asp440 is recognized by H2B Ser136 and the carboxyl of the OsChz1 Asp440 interacts with the side chain of H2B His133. Last, the C-terminal half of OsChz1-C inserts into the acidic pocket of H2A, formed by α2–α4 helices. As depicted in Fig. 3c, the side chain of OsChz1 Asn444 forms H-bond interaction with H2A Tyr59 and the side chain of OsChz1 Glu446 is recognized by H2A Leu67 via van der Waals interaction (Fig. 3c). These above histone-binding residues of OsChz1 are different from those of yChz1 involved in binding with H2A.Z-H2B (Supplementary Fig. 5), as defined either based on the NMR structure of yChz1-H2A.Z-H2B^29^ or the crystal structure of yChz1-H2A.Z-H2B^33^. When the H2A-H2B dimer was considered within the nucleosome structure (PDB ID: 1AOI), our predicted structure indicates that OsChz1 binds to the disk face of the nucleosome: the C-terminus extends into acidic pocket of the nucleosome and the N-terminus is incompatible with C-terminus α2 helix of histone H4 (Supplementary Fig. 6).

Next, we mutagenized the key residues of OsChz1 involved in histone binding to examine their function in biochemical assays (Supplementary Table 2). It was observed that all the three OsChz1-C mutants exhibited ~15–26-fold decrease of affinity in binding H2A-H2B as compared to the wild-type OsChz1-C (Fig. 3d). Accordingly, compared to the wild-type OsChz1, the mutant of full-length OsChz1 (OsChz1-Mut) showed a drastic reduction of efficiency in facilitating assembly of either H2A-H2B or H2A.Z-H2B with the (H3-H4)2-DNA tetrasome complex (Supplementary Fig. 7).

### Mutations in *OsChz1* caused multiple plant developmental defects

**Fig. 4:**
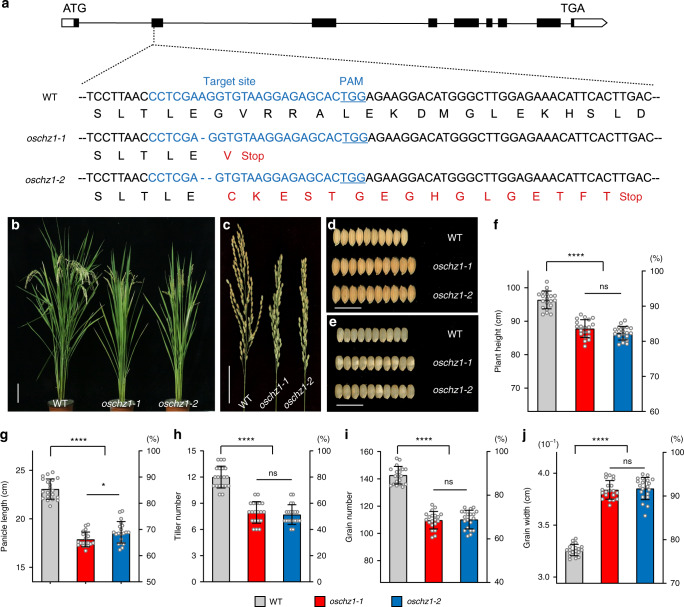
Characterization of *OsChz1* function via CRISPR/Cas9-induced mutagenesis. **a** Schematic diagram of the *OsChz1* gene structure and the mutants generated through CRISPR/Cas9 target. UTRs, exons, and introns are indicated by blank rectangles, black rectangles, and black lines, respectively. The sequence of the targeted site with the underlined protospacer adjacent motif (PAM) and altered amino acid sequence in the *oschz1-1* and *oschz1-2* mutants are highlighted in blue and red, respectively. **b** Representative 95-day-old plants of mutants compared to wild-type (WT) grown under long-day (LD) photoperiod conditions. Scale bar, 10 cm. **c** Comparison of the main panicle morphology between WT and the *oschz1* mutants. Scale bar, 5 cm. **d** Comparison of mature grains between WT and the *oschz1* mutants. Scale bar, 1 cm. **e** Comparison of brown rice grain between WT and the *oschz1* mutants. Scale bar, 1 cm. **f**–**j** Quantitative analysis of agronomic traits for plant height (**f**), panicle length (**g**), tiller number (**h**), grain numbers (**i**), and grain width (**j**), respectively. Values are shown as means ± standard deviation from *n* = 20 individual plants. Statistic significances were determined by two-tailed, paired Student’s *t*-test: *p < 0.05; ****p < 0.001; ns (not significant), *p* > 0.05. The exact *p* values are 1.88 × 10^−12^ (WT vs. *oschz1-1*), 6.67 × 10^−16^ (WT vs. *oschz1-2*), 0.0699 (*oschz1-1* vs. *oschz1-2*) for **f**; 4.08 × 10^−20^ (WT vs. *oschz1-1*), 1.41 × 10^−15^ (WT vs. *oschz1-2*), 0.0204 (*oschz1-1* vs. *oschz1-2*) for **g**; 1.56 × 10^−12^ (WT vs. *oschz1-1*), 8.22 × 10^−14^ (WT vs. *oschz1-2*), 0.5079 (*oschz1-1* vs. *oschz1-2*) for **h**; 1.12 × 10^−18^ (WT vs. *oschz1-1*), 5.48 × 10^−18^ (WT vs. *oschz1-2*), 0.8124 (*oschz1-1* vs. *oschz1-2*) for **i**; 5.63 × 10^−25^ (WT vs. *oschz1-1*), 3.36 × 10^−23^ (WT vs. *oschz1-2*), 0.6914 (*oschz1-1* vs. *oschz1-2*) for **j**. Source data underlying **f**–**j** are provided as a Source Data file.

To investigate the biological function of OsChz1, we used the CRISPR/Cas9-mediated mutagenesis to target the *OsChz1* gene within the rice genome. The designed single-guide RNA (sgRNA) targets a region within the second exon of *OsChz1* (Fig. 4a). The resulted mutation was identified through genotyping and sequencing analysis of transgenic rice plants. Two independent mutations, one with a 1-nt deletion (*oschz1-1*) and the other with a 2-nt deletion (*oschz1-2*) were obtained. Each of these mutations introduces a stop codon that causes premature termination of translation (Fig. 4a), implying both *oschz1-1* and *oschz1-2* as loss-of-function gene mutant alleles. From the transgene-free *oschz1-1* and *oschz1-2* homozygous mutant plants (Supplementary Fig. 8), we further sequenced and conformed that no off-target mutations had been produced, based on analyses of the five top-ranking potential off-target sites predicted by CRISPR-P (Supplementary Table 3).

Next, we analyzed the growth and developmental phenotypes of the *oschz1-1* and *oschz1-2* mutants. Compared to the wild-type plant (WT), both *oschz1-1* and *oschz1-2* mutant plants displayed similarly late-flowering and dwarf phenotypes (Fig. 4b). In addition, the mutants showed less-well developed panicles (Fig. 4c) but bigger kernels (Fig. 4d) and grains (Fig. 4e). Quantitative analyses revealed that the mutants, as compared to WT, exhibited a reduction of about 9% in plant height (Fig. 4f), a reduction of 22% in panicle length (Fig. 4g), a reduction of 30% in tiller number per plant (Fig. 4h), a reduction of 23% in grain number per panicle (Fig. 4i), and an increase of about 18% in grain width (Fig. 4j). These results indicate that *OsChz1* is involved in the regulation of multiple processes of rice growth and development, which is consistent with its broad expression pattern in plants (Fig. 1b–h).

To verify that it is indeed the loss of function of *OsChz1* causing the mutants phenotypes, we performed rescue experiments using a construct containing the full-length coding sequence of *OsChz1* fused to the 4×MYC-tag-coding sequence, together driven by the *OsChz1* native promoter (*pOsChz1::OsChz1-4×MYC*). This transgene was introduced into both the *oschz1-1* and *oschz1-2* mutants, and we obtained 20 independent transgenic lines for each complemented line. All stable transgenic lines showed similarly rescued phenotypes, and subsequently, we analyzed in detail one complemented line for each of the mutants, i.e., COM1 for *oschz1-1* and COM2 for *oschz1-2*. Both COM1 and COM2 are phenotypically similar to WT (Supplementary Fig. 9a and b). They have expression levels of *OsChz1* similar to that of WT (Supplementary Fig. 9c), and the OsChz1-MYC fusion protein was detected in COM1 and COM2 (Supplementary Fig. 9d). They also display WT-levels of heading date, plant height, tiller number, panicle length, grain number, and grain width (Supplementary Fig. 9e–j). Together, these data confirmed that the loss of function of *OsChz1* is responsible for the *oschz1* mutant phenotypes.

### *OsChz1* promotes plant flowering through the *Ehd1-Hd3a/RFT1* activation pathway

**Fig. 5:**
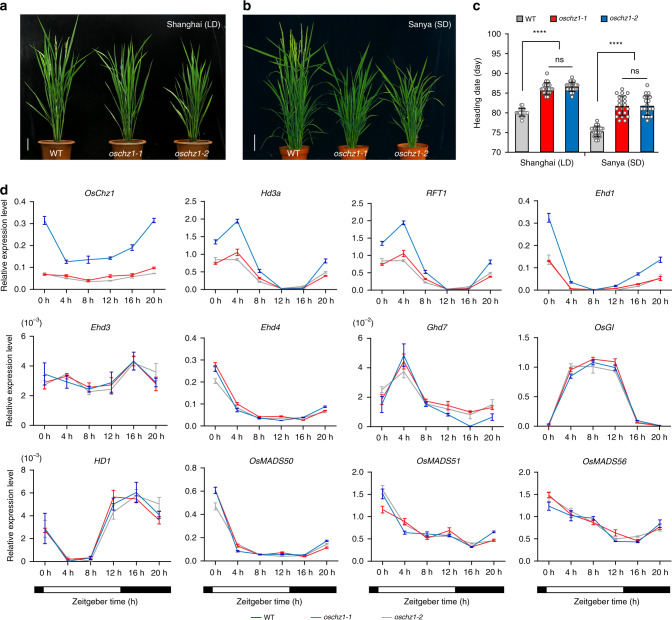
Analysis of the flowering time defects of the *oschz1* mutants. **a**, **b** Representative images of wild-type (WT) and the *oschz1* mutant plants at heading date stage grown under long-day (LD) and short-day (SD) photoperiod conditions, respectively. Scale bar, 10 cm. **c** Heading date measurements of WT and the *oschz1* plants. Values are shown as means ± standard deviation from *n* = 20 individual plants. Statistic significances were determined by two-tailed, paired Student’s *t*-test: ****p < 0.001; ns (not significant), *p* > 0.05. The exact *p* values are 4.93 × 10^−18^ (WT vs. *oschz1-1*), 9.74 × 10^−21^ (WT vs. *oschz1-2*), 0.3432 (*oschz1-1* vs. *oschz1-2*) under LD condition; 2.82 × 10^−12^ (WT vs. *oschz1-1*), 1.50 × 10^−12^ (WT vs. *oschz1-2*), 0.9501 (*oschz1-1* vs. *oschz1-2*) under SD condition. **d** Relative expression levels of various rice flowering time regulatory genes in WT and the *oschz1* mutants. RNA was isolated from leaves of plants grown under LD photoperiods and was analyzed by quantitative RT-PCR. Zeitgeber time is indicated by a white bar for light period and a black bar for dark period. Values are shown as means ± standard deviation from *n* = 3 independent biological replicates, after normalization using rice *Ubiqutin* 5 as internal control. Source data underlying **c** and **d** are provided as a Source Data file.

Flowering represents a key developmental transition during the plant life cycle. To further investigate the flowering phenotype of the *oschz1* mutants in detail, we analyzed the heading date of rice plants grown under either natural long-day (LD; at Shanghai in China) or short-day (SD; at Sanya in China) photoperiod conditions. Both the *oschz1-1* and *oschz1-2* mutants showed a late-flowering phenotype under either LD (Fig. 5a) or SD (Fig. 5b) photoperiods, with significantly delayed heading dates (Fig. 5c), indicating that *OsChz1* promotes flowering in a photoperiod-independent manner. To gain insight into gene regulatory network underlying the mutant flowering phenotype, we analyzed the expression levels of *OsChz1* and some key rice flowering-regulatory genes at 4 h time-intervals over a total of 20 h. Plants in heading period grown under natural LD photoperiod conditions were used in the analysis. As shown in Fig. 5d, *OsChz1* exhibited a diurnal expression pattern in WT, with a decrease by dawn and an increase by dusk during the circadian rhythm. As expected, transcripts of *OsChz1* were barely detected in the *oschz1-1* and *oschz1-2* mutants. *Rice Flowering Locus T1* (*RFT1*) and *Heading date 3a* (*Hd3a*) are known as rice florigen genes encoding small FT-like proteins to promote flowering^37,38^. Interestingly, *RFT1* and *Hd3a* transcription levels exhibited a strong diurnal rhythm in WT, whereas both of them were drastically reduced in *oschz1-1* and *oschz1-2*. Similarly, the expression of *Early heading date 1* (*Ehd1*), which encodes a B-type response regulator acting as an inducer upstream of *Hd3a/RFT1*^39–41^, was also reduced in the mutants. In contrast to *Ehd1*, *Hd3a*, and *RFT1*, the other examined genes showed roughly similar expression levels and patterns in the *oschz1-1* and *oschz1-2* mutants as compared to WT (Fig. 5d). Based on these results, we conclude that *OsChz1* promotes rice flowering time through the *Ehd1-Hd3a/RFT1* pathway.

### Loss of OsChz1 affects transcription and nucleosome distribution of the rice genome

**Fig. 6:**
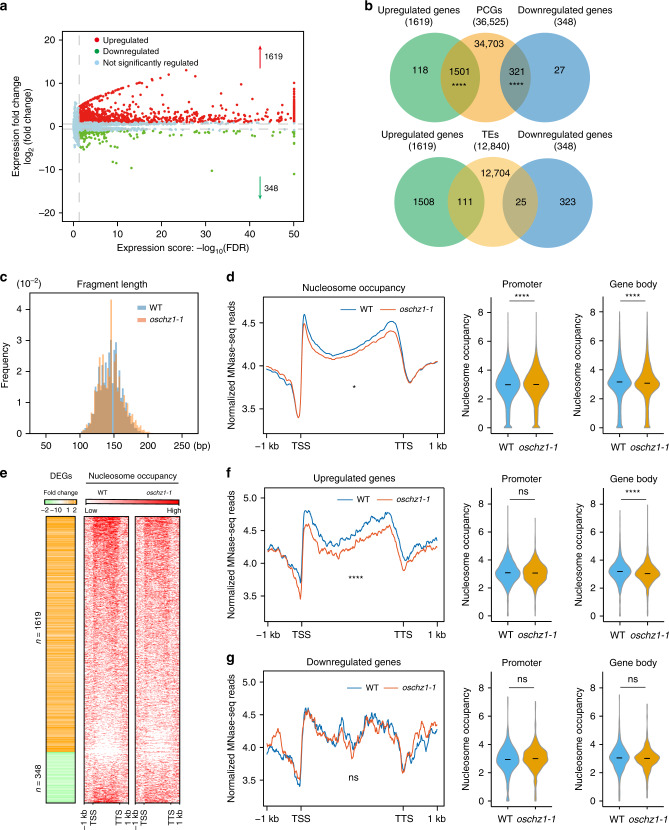
Impact of the OsChz1 loss on gene expression and nucleosome occupancy at the genome-wide level. **a** Comparison of gene expression in wild-type (WT) and *oschz1-1* analyzed by RNA-seq. The *x*-axis indicates the magnitude of expression (expression score = −log 10 FDR) and the *y*-axis indicates fold change in expression. Up- and downregulated genes (red and green dotss, respectively) were identified as those with expression level and change above thresholds (above noise and >1.5-fold change, shown with dashed lines) and with the *p* < 0.05. The adjusted *p* value was calculated with the Benjamini–Hochberg correction. **b** Venn diagram presenting overlap of misregulated genes (green for upregulated genes, blue for downregulated genes) with protein-coding genes (PCGs) or transposable elements (TEs). The number of genes in each cluster is indicated. The adjusted *p* value was calculated with the single-end Fisher’s exact test: ****p < 0.0001. **c** Frequency distribution of nucleosomal DNA size of WT (blue) and *oschz1-1* (orange) obtained in MNase-seq. **d** Genome-wide nucleosome-occupancy profiles in WT and *oschz1-1* (left) and violin plots showing nucleosome-occupancy changes between WT and *oschz1-1* at promoter and gene-body region (middle and right). The plots were generated from 1 kb upstream of the TSS for promoter and from TSS-to-TTS for gene body. Signal for nucleosome is a merge from two biological replicates. Statistic significances were determined by two-tailed Welch two-sample *t*-test: *p < 0.05; ****p < 0.0001; ns (not significant), *p* > 0.05. **e** Heatmap representing the differentially expressed genes (DEGs) and nucleosome occupancy between WT and *oschz1-1*. **f**, **g** Density profiles (left) and violin plots (middle and right) showing nucleosome occupancy of the upregulated and downregulated genes identified in the *oschz1* mutant, respectively. Analyses and statistic assessments were as described in **d**. The exact *p* values are 1.41 × 10^−154^, 1.33 × 10^−32^ for **b**; 0.0102, 1.28 × 10^−5^, 3.61 × 10^−8^ for **d**; 3.48 × 10^−24^, 0.0818, 1.84 × 10^−6^ for **f**; 0.3229, 0.1851, 0.6521 for **g**. Source data underlying **a**, **d**, **f**, and **g** are provided as a Source Data file.

To investigate the effects of loss of OsChz1 on gene transcription at genome-wide level, we performed RNA-seq analysis of the *oschz1-1* mutant and WT rice plants grown under our laboratory LD photoperiods. The analysis was performed in three biological replicates. We identified the differentially expressed genes in mutant compared to WT by using DEseq2^42^. A total of 1967 genes were detected as significantly (fold change ≥ 1.5 and *p*adj < 0.05) misregulated, with 1619 of them upregulated and 348 of them downregulated (Fig. 6a), in the *oschz1-1* mutant as compared to the control WT. Among the downregulated genes in *oschz1-1*, we detected *Ehd1*, *Hd3a*, *RFT1*, and *OsChz1*, conforming our RT-qPCR results (Supplementary Fig. 10a). A statistically significant degree of overlap was observed between these misregulated genes and the total protein-coding genes (PCGs) of the rice genome (Fig. 6b). In contrast, such significant overlap was not detected for transposable elements (TEs; Fig. 6b). Gene ontology (GO) analysis indicated that genes involved in response to stress, defense, and DNA replication were significantly over-represented in the upregulated genes of the *oschz1-1* mutant, and that genes involved in protein phosphorylation and biotic stimulus were significantly over-represented in the downregulated genes of the *oschz1-1* mutant (Supplementary Fig. 10b). The findings of a large number of misregulated protein-coding genes involved in these different key processes are consistent with the pleiotropic growth and developmental phenotypes of the *oschz1* mutants.

To gain insight into the mechanisms underlying misregulation of genome transcription, we performed an MNase digestion assay on chromatin (Supplementary Fig. 10c) and investigated nucleosome distribution in WT and *oschz1-1* by MNase-seq analysis. Paired-end sequencing was performed from two independent MNase-seq libraries with two biological replicates of WT and *oschz1-1*, and then paired-end reads were mapped to unique positions of the rice genome. The nucleosome peaks in WT (Supplementary Fig. 10d) significantly overlapped with the previously published data^43^. The mono-nucleosomal DNA length was roughly 146 bp in size for both WT and *oschz1-1* (Fig. 6c). Detailed mapping at the whole genome level detected in total 25,345 nucleosome-position shifts (12,540 forward shift and 12,805 reverse shift) and 37,608 nucleosome-density alterations (19,673 increased and 17,935 decreased) in *oschz1-1* as compared to WT (Supplementary Table 4). The nucleosome shift was detected majorly within intergenic regions (Supplementary Table 4). In contrast, the nucleosome-density reduction occurred primarily within the gene-body region while increased nucleosome density was observed at the promoter and intergenic regions (Fig. 6d, Supplementary Table 4). These alterations of nucleosome distribution are unlikely to be associated with histone levels because histone genes are expressed at similar levels in *oschz1-1* as in WT (Supplementary Fig. 10e).

Next, we investigated the nucleosome occupancy at the misregulated genes in *oschz1-1* (Fig. 6e). The nucleosome occupancy within the genic regions of upregulated genes was found decreased significantly (Fig. 6f), whereas that of downregulated genes barely changed (Fig. 6g). Similar conclusions were obtained when nucleosome occupancy just adjacent to TSS was analyzed (Supplementary Fig. 11). We further confirmed the correlation of nucleosome-occupancy decrease with upregulated genes by quantitative PCR analysis on several random-selected genes (Supplementary Fig. 12). This association between the decrease of nucleosome occupancy and the upregulation of gene expression is in agreement with the general inhibitory role of nucleosome on transcription, as reported in the previous studies in Arabidopsis, rice, and maize^43–45^. In contrast, for downregulated genes, a statistically significant correlation with nucleosome-occupancy changes could not be established, albeit some slight increase of nucleosome occupancy distantly upstream of TSS (Fig. 6g, Supplementary Fig. 11). Examination of the downregulated flowering genes *Ehd1*, *Hd3a*, and *RFT1* also did not show a significant change of nucleosome occupancy in *oschz1-1* (Supplementary Fig. 13).

### OsChz1 affects H2A.Z levels within the rice genome

**Fig. 7:**
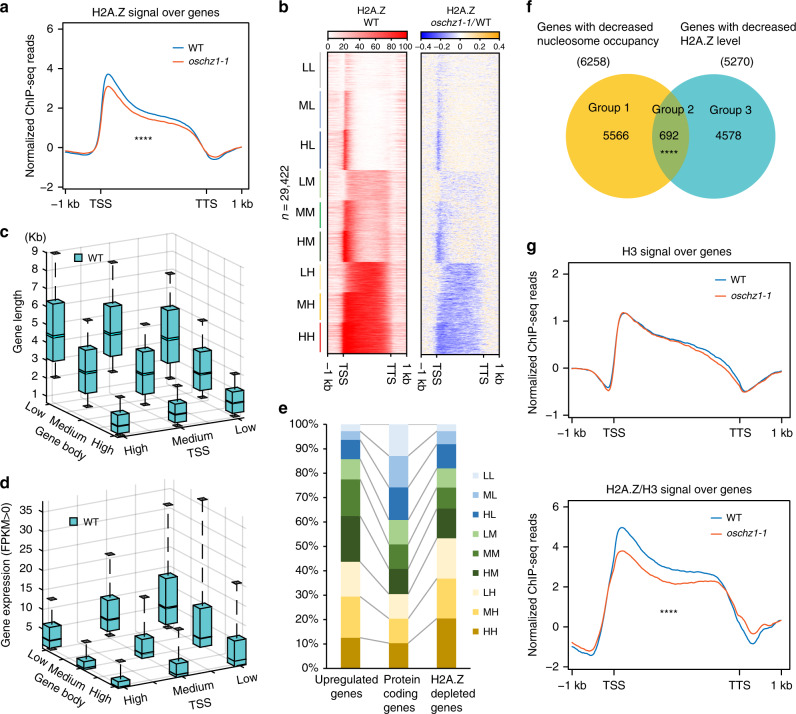
Impact of the OsChz1 loss on H2A.Z enrichment at the genome-wide level. **a** Genome-wide H2A.Z occupancy profiles in wild-type (WT) and *oschz1-1*. The plots were generated from 1 kb upstream of the TSS to 1 kb downstream of the TTS of genes analyzed by ChIP-seq. Signal for H2A.Z is a merge from two biological replicates. Statistic significances were determined by single-end Welch two-sample *t*-test: ****p = 1.01 × 10^−5^. **b** Heatmap showing H2A.Z levels upstream TSS, along gene body and downstream TTS of genes in WT and *oschz1-1*. Genes were divided into nine classes according to the levels of H2A.Z in WT at TSS (low, medium, and high) and gene body (low, medium, and high). Shown are corresponding H2A.Z ratio plots for WT and WT over *oschz1-1*. **c**, **d** Boxplots showing correlation between gene length and H2A.Z levels (**c**), gene expression in FPKM, and H2A.Z levels (**d**) at TSS and within gene body in WT (*n* = 29,422 H2A.Z-enrichment genes). Center line indicates the median, upper and lower bounds represent the 75th and the 25th percentile, respectively, whiskers indicate the minimum and the maximum. **e** Intersection comparisons of H2A.Z-depleted genes (right) and upregulated genes (left) in *oschz1-1* with the nine classes of genes characterized by their distribution of H2A.Z in WT (middle). Labels H, M, and L indicate for high, medium, and low levels of H2A.Z at TSS (first letter) and gene body (second letter), respectively. **f** Venn diagram for the overlap of genes with reduced nucleosome occupancy and genes with decreased H2A.Z in *oschz1-1*. Significance level of the intersection was examined by single-end Fisher’s exact test: ****p = 2.01 × 10^−6^. **g** Genome-wide H3 occupancy profiles in wild-type (WT) and *oschz1-1* (top graph) and that of H2A.Z normalized with H3 (bottom graph). The profiles were generated from 1 kb upstream of TSS to 1 kb downstream of TTS along genes. Statistic significances were determined by single-end Welch two-sample *t*-test: ****p = 4.09 × 10^−15^. Source data underlying **b**, **c**, **d**, and **f** are provided as a Source Data file.

While the alteration of nucleosome distribution observed in *oschz1-1* is consistent with the histone–chaperone function of OsChz1, they cannot specify whether or not OsChz1 plays a role in the regulation of chromatin content of histone variant H2A.Z within the rice genome. Therefore, we analyzed genome-wide H2A.Z enrichment in WT and *oschz1-1* by ChIP-seq experiments. As compared to WT, in *oschz1-1* we detected a total of 5310 decreased H2A.Z peaks (corresponding to 5270 genes), with a consecutive decrease of H2A.Z over the entire gene-body region (Fig. 7a). Examination of all the misregulated genes revealed that both upregulated and downregulated genes exhibit lower levels of H2A.Z in *oschz1-1* as compared to WT (Supplementary Fig. 14). To characterize in more details about the H2A.Z decrease, we categorized genes into nine classes based on H2A.Z levels (low, medium, or high) at TSS and at gene body in all of their different combinations, as described in Coleman-Derr and Zilberman^20^. A decrease of H2A.Z in *oschz1-1* compared to WT was found ubiquitous for all of these nine classes of genes (Fig. 7b), indicating that OsChz1 promotes H2A.Z deposition without any specific class-preference of genes marked by different H2A.Z-enrichment patterns.

Next, we analyzed gene length in relation with H2A.Z enrichment. It was found that shorter genes (<2 kb) contain significantly higher levels of H2A.Z than longer genes (>3 kb) within the gene body (Fig. 7c). We further compared gene expression in relation to H2A.Z enrichment. In WT, gene expression levels were found negatively correlated with H2A.Z levels at either TSS or gene body or both (Fig. 7d). This observation is consistent with previous studies^20,46,47^, together supporting a static repressive effect of H2A.Z on gene transcription. We intersected these classes with genes that lost H2A.Z in *oschz1-1* and misregulated, and then plotted their distribution. This revealed that loss of H2A.Z in *oschz1-1* tends to occur over genes containing high level of H2A.Z, whereas the upregulated genes tend to belong to gene classes containing medium and high H2A.Z levels over gene bodies (Fig. 7e).

A significant loss of H2A.Z was observed in a subset of genes exhibiting reduced nucleosome occupancy in *oschz1-1* (Group 2, Fig. 7f). Meanwhile, a higher number of genes showed separately either a loss of H2A.Z (Group 3, Fig. 7f) or a reduced nucleosome occupancy (Group 1, Fig. 7f). To further investigate H2A.Z enrichment independently from nucleosome occupation changes, we analyzed H2A.Z levels after normalization with the nucleosome core histone H3. It was found that the H3 levels were largely similar and the normalized H2A.Z levels remained significantly reduced across the gene body in *oschz1-1* as compared to WT (Fig. 7g), supporting that OsChz1 plays indeed a specific role in chaperoning H2A.Z in vivo.

### OsChz1 binds chromatin regions containing repressive histone marks

**Fig. 8:**
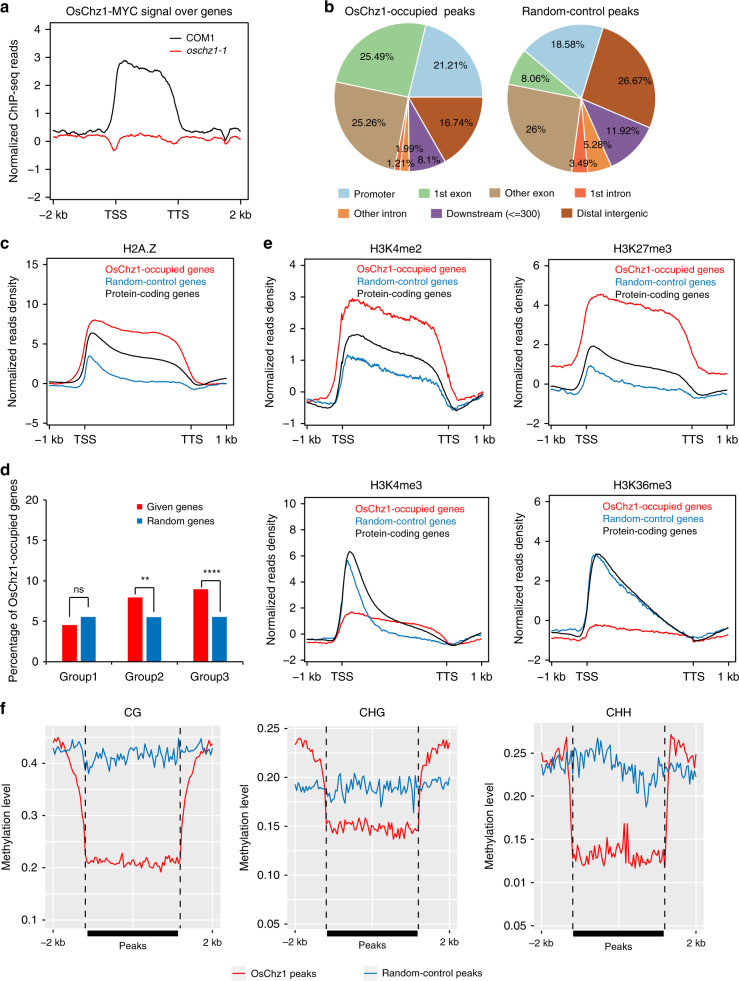
Chromatin-binding characterization of OsChz1. **a** Distribution of normalized signal for OsChz1-MYC ChIP-seq in COM1 and *oschz1-1*. **b** Genomic distribution of OsChz1-enriched peaks compared to a control set of peaks randomly selected from the genome based on number and length. Different genic regions are displayed in different colors as indicated. **c** Average density plots showing the distribution of normalized levels for H2A.Z ChIP-seq in OsChz1-occupied genes (*n* = 3099) compared to protein-coding genes and a random control set of genes. The plots were generated from 1 kb upstream TSS to 1 kb downstream TTS. **d** Enrichment analysis of the OsChz1 binding at genes belonging to the three different groups classified in Fig. 7f (Group 1, *n* = 5566; Group 2, *n* = 692; Group 3, *n* = 4578). Statistic significances were determined by single-end Fisher’s exact test: **p = 0.0049; ****p = 5.86 × 10^−23^, ns (not significant), *p* = 0.9998. **e** Average density plots showing the distribution of normalized levels for H2A.Z, H3K27me3, H3K36me3, H3K4me1, H3K4me2, and H3K4me3 ChIP-seq in OsChz1-occupied genes (*n* = 3099) compared to protein-coding genes and a random control set of genes. The plots were generated from 1 kb upstream TSS to 1 kb downstream TTS. **f** Average distribution of DNA-methylation levels in CG, CHG, and CHH contexts in WT over OsChz1-occupied peaks (red, *n* = 4865 peaks) compared to a control set of peaks (blue) randomly selected from the genome based on number and length. Source data underlying **a** are provided as a Source Data file.

To investigate the localization of OsChz1 in chromatin, we performed ChIP-seq analysis by using the *pOsChz1:OsChz1-4×MYC* transgenic line (COM1) to detect OsChz1-MYC and using *oschz1-1* to eliminate background signal (Fig. 8a). Data analysis with MACS2^48^ allowed us to define 4865 OsChz1-occupied peaks, corresponding to 3099 genes. The OsChz1-occupied peaks showed a preference for promotor and first exon compared to a control set of peaks randomly selected from the genome (Fig. 8b). Consistently, OsChz1 peaks contained higher levels of histone H2A.Z when compared to a random set of control genes or all protein-coding genes (Fig. 8c). A significant overlap was observed for the OsChz1 binding with genes showing loss of H2A.Z (alone or together with reduced nucleosome occupancy) but not with those genes exhibiting reduced nucleosome occupancy alone (Fig. 8d). It is likely that some nucleosome-occupancy changes may not require a steady binding of OsChz1 on chromatin. We further investigated the preferences of OsChz1-occupied genes for histone modifications using recently published data from similar stage of rice plant development^49,50^. Obviously, OsChz1-occupied genes containing higher levels of H3K27me3 over the whole gene region when compared to a random control set of genes or genes averages, especially in gene body (Fig. 8e). Similarly, OsChz1-occupied genes tend to show higher enrichment of H3K4me2 (Fig. 8e), a histone mark recently defined as associated with transcription repression in higher plants^49^. By contrast, the lower levels of activating histone modifications H3K36me3 and H3K4me3 were detected in those genes when compared to a random control set of genes (Fig. 8e). Altogether, these results indicate that OsChz1 preferentially localizes to the regions that contained high levels of repressive histone marks.

Last, since some previous studies documented anti-correlation between H2A.Z and DNA methylation^16,20,51,52^, we performed bisulfite sequencing (BS-seq) and generate single-nucleotide resolution maps of cytosine methylation. It was found that the DNA-methylation levels in all of the three nucleotide sequence contexts (CG, CHG, and CHH; with H being A, C, or T) are lower at OsChz1-binding peaks as compared to the control set of peaks over the genome (Fig. 8f), suggesting that OsChz1 binds with a preference for low DNA-methylation regions. The CG, CHG, and CHH methylation in WT was found at 43.8%, 20.3%, and 2.4%, respectively; and that in *oschz1-1* was found at 42.6%, 20.1%, and 2.5%, respectively. These comparable levels between WT and *oschz1* indicate that the loss of *OsChz1* barely affects the methylation landscape of the rice genome (Supplementary Fig. 15a). To further investigate the relationship between histone H2A.Z and DNA methylation, we plotted the levels of DNA methylation over the 5,270 genes with reduced H2A.Z deposition in *oschz1-1*. At these regions, the level of methylation in all cytosine contexts was unaffected in *oschz1-1* (Supplementary Fig. 15b). This revealed that the methylation state of these genes did not change with the decrease of H2A.Z in *oschz1-1*.

## Discussion

In this study, we identified OsChz1 as a histone–chaperone for both the canonical H2A and the variant H2A.Z. The OsChz1 protein binds in vitro both the H2A-H2B and H2A.Z-H2B dimers, with a similar or higher affinity for the canonical H2A than for the variant H2A.Z. This differs from yChz1, which was shown to bind preferentially the H2A.Z-H2B dimer over the H2A-H2B dimer^28^. Two structural domains were reported as responsible for the binding preference of yChz1 to H2A.Z over H2A: a middle region, embracing the CHZ-motif, binds the highly conserved H2A.Z-specific residues Gly98 and Ala57 and dictates a modest preference for H2A.Z-H2B; and the C-terminal region harboring the DEF/Y motif engages an arginine finger and a hydrophobic pocket in H2A.Z-H2B and enhances majorly the binding preference for H2A.Z-H2B^33^. Both the sequence involved in H2A.Z Gly98-binding and the DEF/Y motif of yChz1 are poorly conserved in CHZ-domain proteins from higher eukaryotes (Supplementary Fig. 5).

Our crystal structure analysis of the OsChz1-H2A-H2B complex showed that OsChz1 binds H2A-H2B via an acidic binding surface. Mutagenesis unraveled that the OsChz1 residues Glu432 + Glu433, Glu436 + Glu 438 + Asn439 + Asp440, or Asn444 + Val445 + Glu446 all are functionally involved in the OsChz1 binding with H2A-H2B as well as with H2A.Z-H2B. In vitro nucleosome assembly assay also indicated that OsChz1 could enhance the assembly of both H2A-H2B and H2A.Z-H2B with a (H3-H4)_2_-DNA tetrasome. The H2A residues Tyr59, and Leu67 involved in binding with OsChz1 in the OsChz1-H2A-H2B complex are conserved in H2A.Z, likely providing an explanation for OsChz1 in chaperoning both H2A-H2B and H2A.Z-H2B. These residues are also conserved in the variant H2A.W and H2A.X (Supplementary Fig. 1b), which marks constitutive heterochromatin and can be phosphorylated upon DNA damage, respectively^53^. Whether or not can OsChz1 bind to H2A.W and H2A.X remains unknown. Moreover, the N-terminus-shortened OsChz1-C showed higher binding-affinity to H2A-H2B over H2A.Z-H2B, but both the molecular basis and the functional significance of this preference remains unclear. Last, the OsChz1-M fragment also displayed binding activity to H2A-H2B and H2A.Z-H2B, raising the question of whether one OsChz1 molecule binds two molecules of H2A-H2B/H2A.Z-H2B. Future investigation about these different issues will extend our knowledge on the function and molecular mechanisms of OsChz1 in histone chaperoning.

At nucleosome level, the OsChz1-H2A-H2B complex structure predicts that the binding is located at the disk face of the nucleosome where the C-terminus of OsChz1 prolongs into the acidic pocket of nucleosome and the N-terminus counteracts with the C-terminus α2 helix of histone H4. This differs from the NAP1 histone–chaperone that shields the DNA-binding surface of H2A-H2B. Analyses of the yNAP1-H2A-H2B complex structure showed that a yNAP1 homodimer forms an acidic binding surface and engages two copies of H2A-H2B^54^ or a single H2A-H2B heterodimer^55^. The yNAP1-H2A-H2B inhibitory conformation was proposed to serve in avoiding inappropriate binding of H2A-H2B with DNA and/or in facilitating H2A-H2B transport and storage in the cell. OsChz1 is localized exclusively to the nucleus, which is in agreement with the idea that Chz1 acts primarily in the nucleus in nucleosome assembly and/or in the replacement of H2A-H2B by H2A.Z-H2B from the nucleosome^32^. The fact that deprivation of Chz1 caused some specific mutant phenotypes in both yeast^28^ and rice (this study) further underlined some distinct biological functions of Chz1 versus NAP1. Distinct from the sole OsChz1, NAP1 is represented by a family of 5–6 members in rice or Arabidopsis^25^. Interestingly, a recent study showed that the Arabidopsis NRP1 and NRP2 negatively regulate H2A.Z abundance in chromatin and their loss causes over-accumulation of H2A.Z genome-wide, especially at heterochromatin regions normally depleted for H2A.Z in wild-type plants^34^. In parallel to specific functions, it is worth to note that redundant functions also exist between Chz1 and NAP1. Analyses of the yeast mutants revealed that yChz1 and yNAP1 can reciprocally substitute for the binding to H2A.Z-H2B and simultaneously loss of both yChz1 and yNAP1 aggravates defects in H2A.Z deposition onto nucleosomes^28,31^.

In rice, loss of OsChz1 caused genome-wide modifications of nucleosome occupancy, including nucleosome-position shifts and local nucleosome-density alterations. It was found that genes with decreased nucleosome occupancy are upregulated and vice versa genes with increased nucleosome occupancy are downregulated in expression in the *oschz1* mutant. This result is consistent with the previous knowledge that nucleosome compaction is inhibitory to DNA access by transcription factors. In spite of a diminished binding preference of OsChz1-C for H2A.Z-H2B in vitro, decreased levels of H2A.Z were majorly detected within gene-body chromatin in *oschz1-1*. Thus, similar to yChz1, OsChz1 also plays a role in H2A.Z deposition into chromatin. H2A.Z incorporation into chromatin is catalyzed by SWR1-c, and coordinatively yChz1 acts in delivering H2A.Z to SWR1-c^27,28^. Components of SWR1-c are conserved in eukaryotes and studies of some SWR1-c components in Arabidopsis mutants have also demonstrated their roles in H2A.Z incorporation into chromatin^56^. Future studies will be necessary to investigate precise roles of Chz1 and SWR1-c during H2A.Z deposition in plants as well as in other higher eukaryotes.

A genome-wide anti-correlation between DNA methylation and H2A.Z was reported previously in Arabidopsis^16^ and rice^23^ as well as animals^52,57^. In Arabidopsis, against this general anti-correlation rule, some specific chromatin regions are characterized by co-existence of DNA methylation and H2A.Z. Nonetheless, in some of these specific regions, H2A.Z interacts with the DNA-demethylase ROS1 to prevent hypermethylation of DNA^51^. In contrast to the prediction by the anti-correlation rule, the decreased level of H2A.Z was observed without any detectable increase of DNA methylation in the rice *oschz1* mutant (this study). Also, loss of H2A.Z in Arabidopsis has only a minor effect on DNA methylation^20^, and the *nrp1-1nrp2-2* mutant displays H2A.Z accumulation but largely unaltered DNA methylation or even slight increases of CG/CHG methylation at some TEs^34^. Together, these studies support the proposition that the global anti-correlation between DNA methylation and H2A.Z is primarily caused by the exclusion of H2A.Z from methylated DNA^20^. In our study, we detected OsChz1 binding with a preference to chromatin regions enriched by the repressive histone marks H3K27me3 and H3K4me2. H2A.Z and H3K27me3 are functionally linked and both are involved in transcription repression^58^. While H3K4me3 marks active transcription, H3K4me2 is also associated with transcription repression in rice and in Arabidopsis^49^. Consistent with the association of OsChz1 with these repressive histone marks, loss of OsChz1 caused massive upregulation of genes in the *oschz1* mutant.

Many of the misregulated genes in *oschz1* are predicted as functionally involved in response to diverse stimuli. This is in line with H2A.Z as a major player in plant response to phytohormones and environmental cues^22,59^. Plant flowering time is tightly controlled by complex gene networks that integrate endogenous and environmental cues^39,60^. Loss of OsChz1 caused downregulation of the rice florigens *Hd3a* and *RFT1* as well as their upstream activator *Ehd1*. Nucleosome density and distribution were found not significantly changed at chromatin of these flowering genes. Slight increase of H2A.Z was noticed at 5′-end of *Ehd1* and 3′-end of *RFT1* (Supplementary Fig. 13), which is in line with the repressive role of H2A.Z on transcription but is in contrast to the global function of OsChz1 in H2A.Z deposition. So far, it is unclear whether OsChz1 has regulated the expression of these flowering genes directly or indirectly. Transcription of *Hd3a*/*RFT1*/*Ehd1* is regulated, positively or negatively, by several histone modifications^39,61,62^. As a histone–chaperone, OsChz1 may affects these modifications. While its precise function remains to be determined, the human and vertebrate CHZ-domain protein HIRIP3 had been identified as an interacting-protein of the H3.3-chaperone HIRA and had been shown to also bind H3 and H2B in vitro^63^. In the same line of assumptions, sequences in addition to CHZ-domain (e.g., OsChz1-M) may also contribute to OsChz1 binding with histones and/or with other regulatory proteins. Last, in addition to flowering time, several other agronomic traits (e.g., plant architecture, reproductivity, and grain size) are affected by loss of OsChz1. Molecular mechanisms underlying these effects are still obscure. Future studies will be necessary to address these different issues, which will undoubtedly shed further light on molecular mechanisms of CHZ-domain proteins in multilayer regulations of chromatin remodeling, genome function, gene transcription, and organism growth and development.

## Methods

### Phylogenetic analysis

The full-length amino acid sequences of CHZ-domain proteins were used for the phylogenetic tree analysis. Homology sequence searches were carried out in the database of UniProt (https://www.uniprot.org/). The phylogenetic analysis was conducted by using MEGA version 7.0.26 with bootstrapping set at 1000 replicates and illustrated by using FigTree version 1.4.3. The conserved protein domains were identified by conducting searches in NCBI (https://www.ncbi.nlm.nih.gov/Structure/cdd/wrpsb.cgi).

### RT-PCR

Total RNA was extracted from different tissues of rice (*Oryza sativa* ssp. *japonica* cv Nipponbare) using an RNAprep pure Plant Kit (Tiangen Biotech, Beijing, China) and RNA was reverse-transcribed using PrimeScript^TM^ RT reagent Kit (TaKaRa, RR047A). qRT-PCR reactions were carried out on CFX_Connect^TM^ (BIO-RAD) with TB Green^TM^ Premix Ex Taq^TM^ II (TaKaRa, RR820A). Gene-specific primers are listed in Supplementary Table 5. Each group data comprised three biological replicates and *Ubiquitin5* was used as a reference gene to normalize the gene expression data.

### GUS reporter analysis

For tissue-specific expression analysis, a 2234 bp fragment upstream from the TSS of *OsChz1* was cloned into the pCAMBIA1391Z vector to generate *pOsChz1:GUS* transgenic plants. β-Glucuronidase activity was analyzed using *pOsChz1:GUS* transgenic rice plant tissues by histochemical staining assay. Briefly, plant samples were fixed by incubation in 90% acetone on ice for 30 min, then rinsed in the washing buffer (30.75 mM K_2_HPO_4_, 19.25 mM KH_2_PO_4_, 0.1% Triton X-100, 0.5 mM K_3_Fe[CN]_6_, 0.5 mM K_4_Fe[CN]_6_, and 5 mM EDTA), and placed in the staining buffer (30.75 mM K_2_HPO_4_, 19.25 mM KH_2_PO_4_, 0.1% Triton X-100, 0.5 mM K_3_Fe[CN]_6_, 0.5 mM K_4_Fe[CN]_6_, 0.5 mg/ml X-Gluc, and 5 mM EDTA). After vacuum infiltration for 15 min, the staining reaction was kept by incubation at 37 °C overnight. Chlorophylls were removed by incubation in a solution of 70% ethanol.

### Pull-down assay

Full-length and truncated versions of *OsChz1* were PCR-amplified and cloned into the pGEX-6P-1 (GE Healthcare, Milwaukee, WI, USA). The PCR primers are listed in Supplementary Table 5. Recombinant GST-fusion proteins were produced in *Escherichia coli* BL21 (DE3) and purified^64^. The Arabidopsis histone H2A (HTA1; AT5G54640) and H2B (HTB1; AT1G07790) dimer was obtained from a previous study^35^, and the co-expressed H2A.Z (HTA9; AT1G52740)-H2B (HTB1) dimer was produced similarly. Briefly, bacteria cells expressing the histone dimers were disrupted by high-pressure cell disruptor in the lysis buffer containing 20 mM Tris-HCl pH 8.0 and 250 mM NaCl. After centrifugation at 34,000 × *g* for 60 min, the supernatant was loaded onto the SP column (GE Healthcare), and eluted by running a linear gradient of 10 column volumes of the elution buffer containing 20 mM Tris-HCl pH 8.0 and 1 M NaCl. Fractions were collected and diluted to 20 mM Tris-HCl pH 8.0 and 500 mM NaCl by adding 20 mM Tris-HCl pH 8.0. The sample was then loaded onto the Heparin column (GE Healthcare), and eluted by running a linear gradient of 20 column volumes of the elution buffer containing 20 mM Tris-HCl pH 8.0 and 2 M NaCl. Fractions were collected, concentrated, and then loaded into the pre-equilibrated column (Superose^TM^ Increase 200 10/300, GE Healthcare) with the buffer containing 20 mM Tris-HCl pH 8.0 and 1 M NaCl. For the GST pull-down assay, beads (GE Healthcare) coated with GST or GST-fusion proteins were incubated at 4 °C for 2 h with the histone H2A-H2B or H2A.Z-H2B dimers in the binding buffer containing 137 mM NaCl, 2.7 mM KCl, 10 mM Na_2_HPO_4_, 2 mM KH_2_PO_4_, pH 7.4, 0.2% Nonidet P-40, and 1 mM DTT. After washing three times with a buffer containing 20 mM Tris-HCl pH 8.0, 200 mM NaCl, 0.2% Nonidet P-40, and 1 mM DTT, the samples were analyzed on SDS-PAGE followed by Coomassie Brilliant Blue staining.

### Protein expression in protoplasts and Co-IP assay

Protoplast isolation and transformation were performed using 10-day-old rice leaf sheaths and stems grown at ∼28 °C in the dark, based on established procedure^65^. The full-length cDNA of rice *H2A* (*HTA702*), *H2A.Z* (*HTA713*), and *OsChz1* were amplified and cloned into the pRTVcHA, pRTVcMYC, and pRTVnGFP vectors^66^, respectively. The leaf sheaths and stems were cut into 0.5-mm strips and incubated in enzyme solution (1.5% cellulose RS, 0.3% macerozyme, 0.4 M mannitol, 2 mM MES, 1 mM CaCl_2_, 0.1% BSA, pH 5.7) for 4 h in the dark. After removing the enzyme solution, the tissues were suspended in W5 buffer (154 mM NaCl, 125 mM CaC1_2_, 5 mM KCl, 2 mM MES, pH 5.7) for 1 h to release the protoplasts. After flow through filter, the protoplasts were collected and resuspended in the suspension medium (0.4 M mannitol, 20 mM CaCl_2_, 5 mM MES, pH 5.7). For transformation, 10 μg plasmid DNA was added to 100 μl protoplast suspension and then mixed with 40% PEG buffer (40% (W/V) PEG4000, 0.4 M mannitol, 100 mM CaCl_2_, pH 5.7) for 20 min. After adding W5 buffer to dilute the mixture and removing the supernatant, the protoplasts were incubated 12−16 h in W5 buffer for protein expression. Expression of GFP-OsChz1 in the protoplasts was detected by fluorescence and imaged under a confocal microscope.

For Co-IP, GFP-OsChz1 was co-expressed together with HTA702-HA or HTA713-MYC in rice protoplasts. The protoplasts were harvested in lysis buffer (50  mM Tris-HCl, pH 8.0, 150 mM NaCl, 0.5% Triton X-100, 1 mM EDTA, 2 mM DTT, protease inhibitor cocktail) and sonicated at 4 °C for five times, 20 s each. Total protoplast extract was immunoprecipitated by anti-GFP antibody (M20004, Abmart, 1:100 dilution) in combination with pre-cleared magnetic protein A beads (10002D, Invitrogen) at 4 °C for 2 h. The immunoprecipitates were washed two or three times with the buffer containing 2 mM KH_2_PO_4_, 10 mM Na_2_HPO_4_, 140 mM NaCl, 2.7 mM KCl, 0.05% (v/v) Tween 20, and analyzed by western immunoblot using anti-HA (ab9110, Abcam, 1:2000 dilution), anti-MYC (M20002L, Abmart, 1:5000 dilution), or anti-GFP antibodies (M20004L, Abmart, 1:5000 dilution). Signals were detected using Chemiluminescence Imaging System (ChemiScope 3600 Mini, ClINX).

### ITC and protein crystal structure determination

DNA fragments encoding 6×His-SUMO-tagged wild-type or mutated OsChz1-C were cloned into the pET28a vector (Novagen). Primers used in cloning are listed in Supplementary Table 5. Recombinant protein expression and purification were performed following the method in Zhu et al.^35^. Briefly, protein expression was induced by 0.2 mM β-D-1-thiogalacto-pyranoside (IPTG), and the induced cultures were grown at 16 °C for 18 h. The final cells were harvested by centrifugation (4000 × *g*) at 16 °C for 15 min and lysed by high-pressure disruptor. Then, the homogenate was clarified by centrifugation (34,000 × *g*) at 4 °C for 1 h. The supernatant was loaded onto Ni-NTA column. The eluted sample was dialyzed and treated with Ulp1 protease to remove the 6×His-SUMO-tagged. Finally, the proteins were purified by size-exclusion chromatography (Superdex 75 16/600, GE Healthcare), concentrated, and stored in the buffer containing 150 mM NaCl, and 20 mM Tris-HCl, pH 8.0.

ITC experiments were performed on a MicroCal iTC200 (GE Healthcare) microcalorimeter by injecting OsChz1-C or its mutants into histone H2A-H2B and H2A.Z-H2B solutions. Titrations were carried out using an initial injection volume of 0.5 μl (omitted from the analysis) followed by 19 injections (each 2 μl) spaced 120 s apart. Data were analyzed using Origin software v7.0, and the heat of dilution was subtracted from the raw values. Dissociation constant (Kd) and stoichiometry (N) values were calculated by fitting the isotherm.

Crystals were obtained by incubation at 18 °C of sitting drops of 0.2 μl protein mixture containing 20 mg/ml H2A(14–106)-H2B(51–148) mixed with OsChz1(338–471) at equal molar ratio in a solution containing 0.2 M Sodium citrate, 0.1 M Tris-HCl pH 8.5, and 30% (w/v) PEG4000. The crystals were transferred to the well solution with 25% (v/v) glycerol before flash freezing in liquid nitrogen. Data collection was performed at Shanghai Synchrotron Radiation Facility beamlines 17U and images were processed with HKL2000 packing (http://www.hkl-xray.com/). The structure was solved by the molecular replacement method using the Phaser MR program in CCP4 v7.0.076^67^. Model improvement was used Coot v0.8.71^68^ and refined using the Refmac program in CCP4. All structural pictures were generated using PyMOL v2.0 (https://pymol.org).

### Nucleosome reconstitution and histone deposition assay

The 187 bp DNA template containing a single Widom 601 sequence was prepared by PCR from plasmid using an unlabeled primer (Supplementary Table 5). The histone octamers, H3-H4 tetramers, H2A-H2B, and H2A.Z-H2B dimers were reconstituted based on established procedure^69^. Briefly, DNA fragments encoding H3 and H4 were cloned into the pETDuet-1 vector (Novagen) for co-expression. The co-expressed H3-H4 tetramer was purified successively by S column (GE Healthcare), Butyl column (GE Healthcare), and size-exclusion chromatography (Superdex 200 16/600, GE Healthcare). For octamer formation, one molar of the H3-H4 tetramer together with two molars of the H2A-H2B dimer was incubated in the refolding buffer containing 2 M NaCl, 10 mM Tris-HCl at pH 7.5, 1 mM EDTA, and 5 mM 2-mercaptoethanol. The resulting octamer was purified through Superdex 200 10/60 (GE Healthcare). The reconstitution reaction mixture with the histone octamers/tetramers and the Widom 601-based DNA template was dialyzed for 16 h at 4 °C in the TEN buffer containing 10  mM Tris-HCl, pH 8.0, 1 mM EDTA, 2 M NaCl. The mixture was then diluted by slow pumping in TE buffer (10 mM Tris-HCl, pH 8.0, 1 mM EDTA) to gradually descending the concentration of NaCl from 2 to 0.6 M. Samples were collected after the final dialysis for 4 h in the HEN buffer containing 10 mM HEPES, pH 8.0, 1 mM EDTA, 150 mM NaCl.

For deposition assay, increasing amounts of H2A-H2B or H2A.Z-H2B dimers mixed with stoichiometric amount of OsChz1, were incubated with H3-H4 tetrasomes in 10 mM HEPES, pH 8.0, 1 mM EDTA, 150 mM NaCl, 1 mM DTT for 30 min on ice. The reaction products were analyzed on 7.5% native polyacrylamide gel in the 1×TG buffer containing 0.025 M Tris-HCl and 0.192 M glycine, run at 4 °C for 3–4 h at 100 V.

### Generation of *oschz1* mutants and phenotype analysis

Mutant plants were generated by using CRISPR/Cas9 system via the custom service of BIOGLE GeneTech (Zhejiang, China). The *OsChz1*-specific oligonucleotide (CCTCGAAGGTGTAAGGAGAGCAC) was designed using CRISPR-P (http://crispr.hzau.edu.cn/CRISPR2/). Rice plant transformation was performed using the *Agrobacterium tumefaciens* strain EHA105 through callus inoculation and plant regeneration. The transgene-free mutant plants were obtained from T3 generation plants. For genotyping and identification of potential off-target sites in *oschz1* mutant lines and of transgene-free mutants, genomic DNA was extracted from young leaves. Genomic regions neighboring the target sites were amplified by PCR and DNA sequencing with specific primers listed in Supplementary Table 5.

For mutant rescue test, the MYC-tagged full-length *OsChz1* sequences were cloned into the pCAMBIA1300 plant expression vector, and the resulting construct *pOsChz1:OsChz1-4×MYC* was introduced into *Agrobacterium tumefaciens* EHA105. The *Agrobacterium* strain was used to transform WT and *oschz1-1* through callus inoculation, plant regeneration, and selection^70^. Next-generation plants were obtained through self-pollination of parents, and plant genotypes were verified through genomic PCR analysis.

Rice plants were grown in the paddies under natural conditions at two locations characterized by different latitudes: Shanghai under LD conditions and Sanya under SD conditions. Plant flowering time was measured by heading date when the first panicle grows out of the flag leaf, and the panicle trails were measured during maturation stage. Each measurement of agronomic characteristics was performed based on an average of over 20 individual plants per sample.

### Genome-wide profiling analyses

Thirty-day-old rice seedlings grown in artificial growth chambers under an LD photoperiod (14 h light at 30 °C and 10 h dark at 28 °C) were used for RNA-seq, ChIP-seq, MNase-seq, and BS-seq analyses. For RNA-seq, total RNA was extracted from over 5 seedlings using an RNAprep pure Plant Kit (Tiangen Biotech, Beijing, China). The strand-specific RNA-seq libraries were constructed by following the KAPA stranded mRNA-seq Kit instructions (Illumina® Platforms, KR0960-v5.17; www.kapabiosystems.com). Briefly, mRNA was prepared based on 10 µg of total RNA by using RNA clean XP beads (BECKMAN COULTER, USA). The poly(A)-enriched RNA was then fragmented to the desired size by heating. Single-stranded cDNA was synthesized with random primers and double-strand cDNA synthesis converted cDNA:RNA hybrid to dscDNA. 3′ Adenine and sequencing adaptors were added, and then the adapter-ligated library DNA was amplified by PCR to enrich the segments to obtain the cDNA library. There were three replicates for each genotype. The library was sequenced on an Illumina HiSeq3000 instrument via the custom service of GENERGY BIO (Shanghai, China).

For MNase-seq, rice seedlings were cross-linked in the fixation buffer, containing 0.4 M sucrose, 10 mM Tris-HCl pH 8.0, 1 mM EDTA, 1 mM PMSF, 1% formaldehyde, under vacuum for 15 min. Cross-linking was stopped by incubation in 0.125 M glycine under vacuum treatment for 10 min. About 1 g powdered leaf material was resuspended in Nuclei Extraction Buffer (50 mM HEPES pH 7.5, 1 mM EDTA pH 8.0, 150 mM NaCl, 1% Triton X-100, 5 mM β-mercaptoethanol and protease inhibitor cocktail (Roche)) under shaking for 30 min at 4 °C. The suspension was then filtered through a Miracloth (Millipore), and nuclei were collected through centrifugation at 3000 × *g* for 20 min at 4 °C. The pellets were washed twice and resuspended in the MNase buffer (50 mm Tris-HCl pH 7.5, 25 mM MgCl_2_, 1 mM CaCl_2_). Chromatin was digested with 2 units of micrococcal nuclease (Sigma) for 10 min at 37 °C. The reaction was stopped with 10 mM EDTA. After centrifugation at 1500 × *g* for 5 min at 4 °C, the supernatant was collected. After treatment with 1 μl of RNase A (10 mg/ml) for 30 min at 37 °C and followed by de-proteinization (0.02 M EDTA, 0.1 M Tris-HCl pH 6.5, 1 μl Protease K (Sigma)) at 45 °C for 3 h, nucleosomal DNA fragments were recovered. Mononucleosome-sized DNA fragments were gel-purified and used to construct a MNase-seq library following the manufacturer’s instructions of VAHTSTM Universal DNA Library Prep Kit (Vazyme). Two replicates for each genotype were obtained and sequenced.

For ChIP-seq, about 5 g rice seedlings were harvested and fixed in the buffer containing 0.4 M sucrose, 10 mM Tris-HCl pH 8.0, 1 mM EDTA, 1% formaldehyde, and 1 mM PMSF. Nuclei were obtained upon lysis in the buffer containing 50 mM HEPES pH 7.5, 150 mM NaCl, 1 mM EDTA, 1% Triton X-100, 5 mM β-mercaptoethano, 10% glycerol, and Protease Inhibitor cocktail. Chromatin was sonicated into DNA fragments below 500-bp in size using the lysis buffer with 1% SDS, and immunoprecipitation was performed using anti-MYC (M20002L, Abmart) at a 1:300 dilution, anti-H2A.Z^60^ at a 1:100 dilution or anti-H3 (ab1791, Abcam) at a 1:300 dilution. After gradual washes using the low-salt buffer (50 mM HEPES pH 7.5, 1 mM EDTA pH 8.0, 150 mM NaCl, and 0.1% Triton X-100), the high-salt buffer (50 mM HEPES pH 7.5, 1 mM EDTA pH 8.0, 500 mM NaCl, and 0.1% Triton X-100), the LiCl buffer (10 mM Tris-HCl pH 8.0, 1 mM EDTA pH 8.0, 0.5% NP-40, 0.25 M LiCl) and the TE buffer (10 mM Tris-HCl pH 8.0, 1 mM EDTA pH 8.0), the immunoprecipitation complex was eluted from the beads twice using elution buffer (1% SDS, 0.1 M NaHCO_3_). The samples were incubated overnight at 65 °C for reverse cross-linking, and followed by RNase A and proteinase K treatments. DNA fragments were then extracted for ChIP-seq library construction following the manufacturer’s instructions of VAHTSTM Universal DNA Library Prep Kit (Vazyme). Two replicates for each genotype were obtained and sequenced.

For BS-seq, total genomic DNA was prepared using the DNeasy plant maxi kit (QIAGEN). Sodium bisulfite treatment, library construction, and sequencing were performed via the custom service of GENERGY BIO (Shanghai, China). Two replicates for each genotype were obtained.

### Seq-data analyses

FastQC v0.11.7 was used to check read quality and size. Raw reads were trimmed to remove bases with low quality score and from adapters by following with filtering short reads by CUTADAPT v1.10^71^. Then, the cleaned reads were aligned to the reference genome of ‘Nipponbare’ (*japonica*) rice (MSU7; http://rice.plantbiology.msu.edu).

For RNA-seq, over 30 million of cleaned stranded paired-end reads were aligned to the rice reference genome by HISAT2 v2.1.0^72^. Samtools v1.9^73^ was used to obtain unique reads. FeatureCounts v1.6.2^74^ was used to assign aligned reads to genes. Differentially expressed genes (DEGs) were identified by the DEseq2 v1.22.2^42^ based on the combined thresholds set as fold change ≥1.5 and padj (the adjusted *p* value) ≤ 0.05. Genome ontology analysis of DEGs was conducted in CARMO online (http://bioinfo.sibs.ac.cn/carmo/Gene_Annotation.php)^75^ and significantly enriched biological functions were identified as a *p* value ≤ 0.05.

For MNase-seq and ChIP-seq, cleaned paired reads were mapped to the rice reference genome by using Bowtie2 v2.3.4.1^76^. Samtools v1.9 was used to remove reads with low mapping quality (*Q* < 20) and potential PCR duplicates. The normalized reads (RPKM) for profiles were calculated with deepTools v3.0.2^77^. Enrichments along the gene body and around TSS and TTS were examined using computeMatrix tool in deepTools. Average density profile was plotted in R and a heatmap was generated using the plotHeatmap tool in deepTools. Bed format files were generated using BED Tools v2.25.0^78^. DANPOS v2.1.3^79^ was used to position nucleosome occupancy and perform statistic test for significant changes. H2A.Z and OsChz1-MYC enrichment regions (peaks) were identified by comparing the ChIP library with input library (parameters: W = 200, G = 200, FDR = 0.05) using SICER.sh from SICER v1.1 software^80^. ChIPpeakAnno v3.16.1^81^ package in R was used to intersect peaks in two replicates to produce the final set of enriched peaks for each sample. DiffBind v2.1.0^82^ was used to compare two different samples to identify differentially enrichment peaks (FDR ≤ 0.05). The peaks were annotated to genes by using ChIPpeakAnno package in R software. Distribution of peaks was obtained by using ChIPseeker v1.18.0^83^. For BS-seq, Bismark v0.22.1^84^ was used to map trimmed paired-end reads to the rice reference genome and to extract methylation regions (DMRs). The methylation levels for profiles and distribution were conducted by using ViewBS v0.1.9^85^.

### Reporting summary

Further information on research design is available in the Nature Research Reporting Summary linked to this article.

## Supplementary information

Supplementary information

Peer Review

Reporting Summary

## Data Availability

Data supporting the findings of this work are available within the paper and its Supplementary Information files. The datasets and plant materials generated and analyzed during the current study are available from the corresponding authors upon request. The RNA-seq, ChIP-seq, MNase-seq, and BS-seq data that support the findings of this study have been deposited to NCBI GEO with the accession number GSE155269. Structural factors and coordinates have been deposited in the Protein Data Bank (PDB) with accession code 6M2M for the OsChz1-H2A-H2B complex. Source data are provided with this paper.

## Source data

Source Data
