# Supplementary material for: OsChz1 acts as a histone chaperone in modulating chromatin organization and genome function in rice
Source: Nat Commun. 2020 Nov 11;11:5717. doi: 10.1038/s41467-020-19586-z (PMC7658359; doi:10.1038/s41467-020-19586-z)
Supplement: Supplementary file 1 — Supplementary information [file 41467_2020_19586_MOESM1_ESM.pdf]

OsChz1 acts as a histone chaperone in modulating chromatin organization and genome function in rice

Du *et al.*

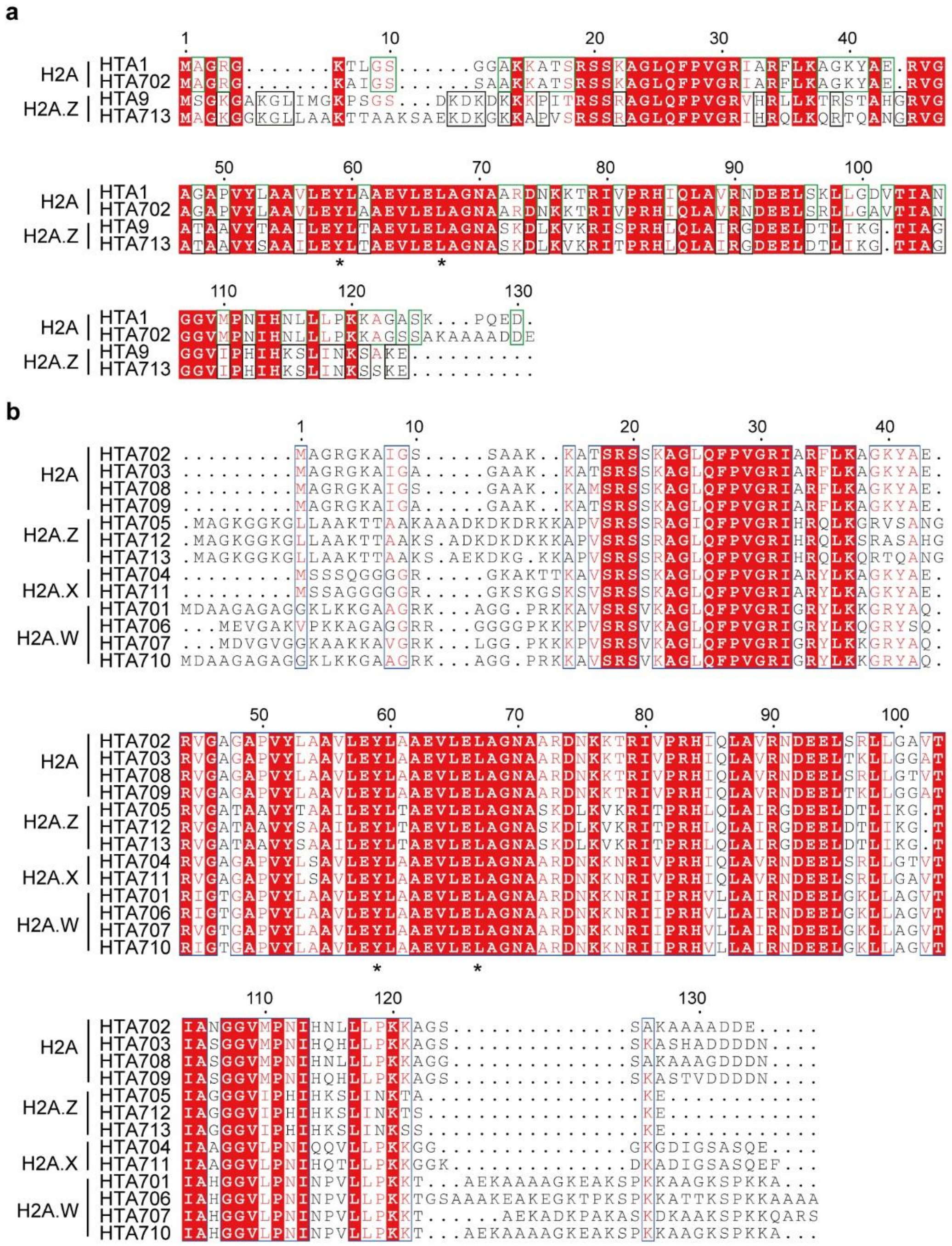

Supplementary Figure 1. Sequence alignments of histone H2A and H2A variants. a Sequence alignments of a canonical histone H2A and a variant histone H2A.Z from rice (HTA702 and HTA713, respectively) and from Arabidopsis (HTA1 and HTA9, respectively). The identical residues in all four histones are highlighted by red background, the residues specifically conserved in H2A are highlighted by green box, and those in H2A.Z by black box. **b** Sequence alignments of all rice canonical histone H2A and H2A variants (H2A.Z, H2A.X and H2A.W). The identical residues in all histones are highlighted by red background. Asterisks indicate amino acids involved in the interaction with OsChz1-C, as determined by the crystal structure of OsChz1-H2A-H2B in this study.

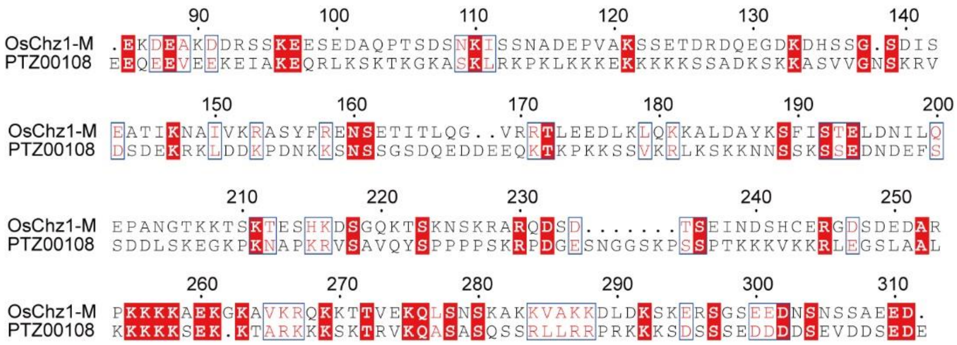

Supplementary Figure 2. Sequence alignments of OsChz1-M together with the conserved protein domain PTZ00108. The identical residues are highlighted by red background, and the similar residues by blue box.

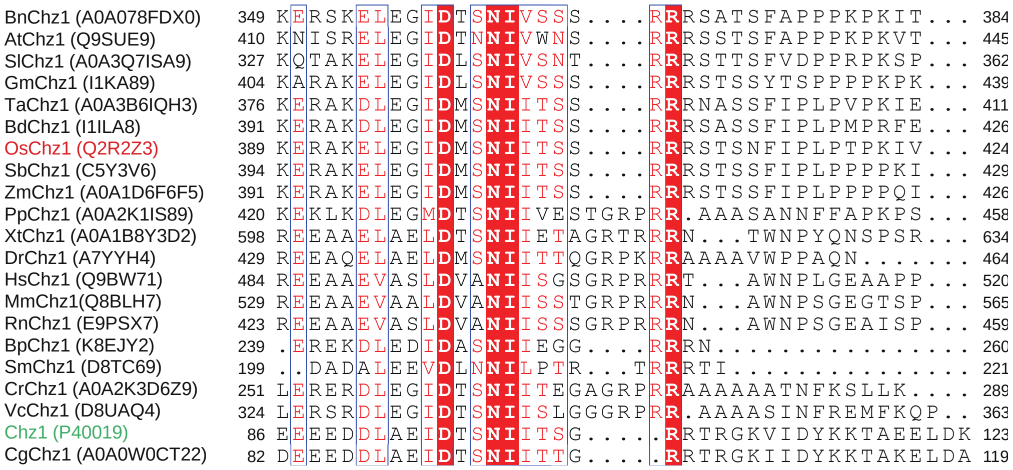

Supplementary Figure 3. Sequence alignments of CHZ domain of proteins identified in different organisms. The identical residues are highlighted by red background, and the similar residues by blue box. Bn, *Brassica napus*; At, *Arabidopsis thaliana*; Sl, *Solanum lycopersicum*; Gm, *Glycine max*; Ta, *Triticum aestivum*; Bd, *Brachypodium distachyon*; Os, *Oryza sativa*; Sb, *Sorghum bicolor*; Zm, *Zea mays*; Pp, *Physcomitrella patens*; Xt, *Xenopus tropicalis*; Dr, *Danio rerio*; Hs, *Homo sapiens*; Mm, *Mus musculus*; Rn, *Rattus norvegicus*; Bp, *Bathycoccus prasinos*; Sm, *Selaginella moellendorffii*; Cr, *Chlamydomonas reinhardtii*; Vc, *Volvox carteri*; Cg, *Candida glabrata*. The green highlights the founding member of the family Chz1 from yeast (*Saccharomyces cerevisiae*).

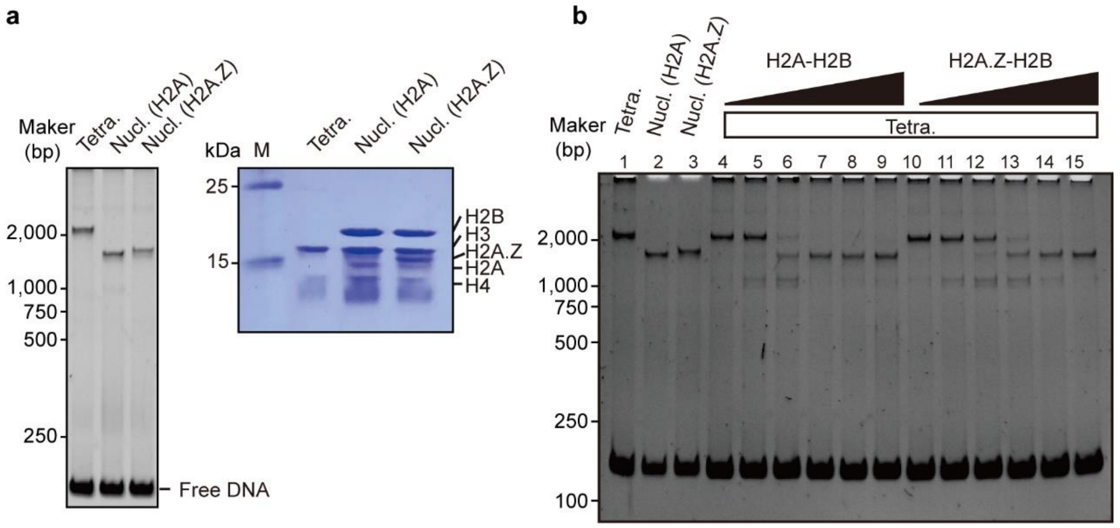

Supplementary Figure 4. Nucleosome assembly *in vitro*. a Verification of migration properties and compositions of the H3-H4-DNA tetrasome (Tetra.) and the mono-nucleosome (Nucl.) containing H2A or H2A.Z. The same samples were analyzed on native PAGE gel stained with GelRed (left panel) and SDS-PAGE gel stained with Coomassie blue (right panel). M, size marker. **b** Native PAGE gel showing assembly of the tetrasome into nucleosome upon reaction with increasing amounts of H2A-H2B or H2A.Z-H2B, as indicated. Experiments were repeated independently three times with similar results. Source data are provided as a Source Data file.

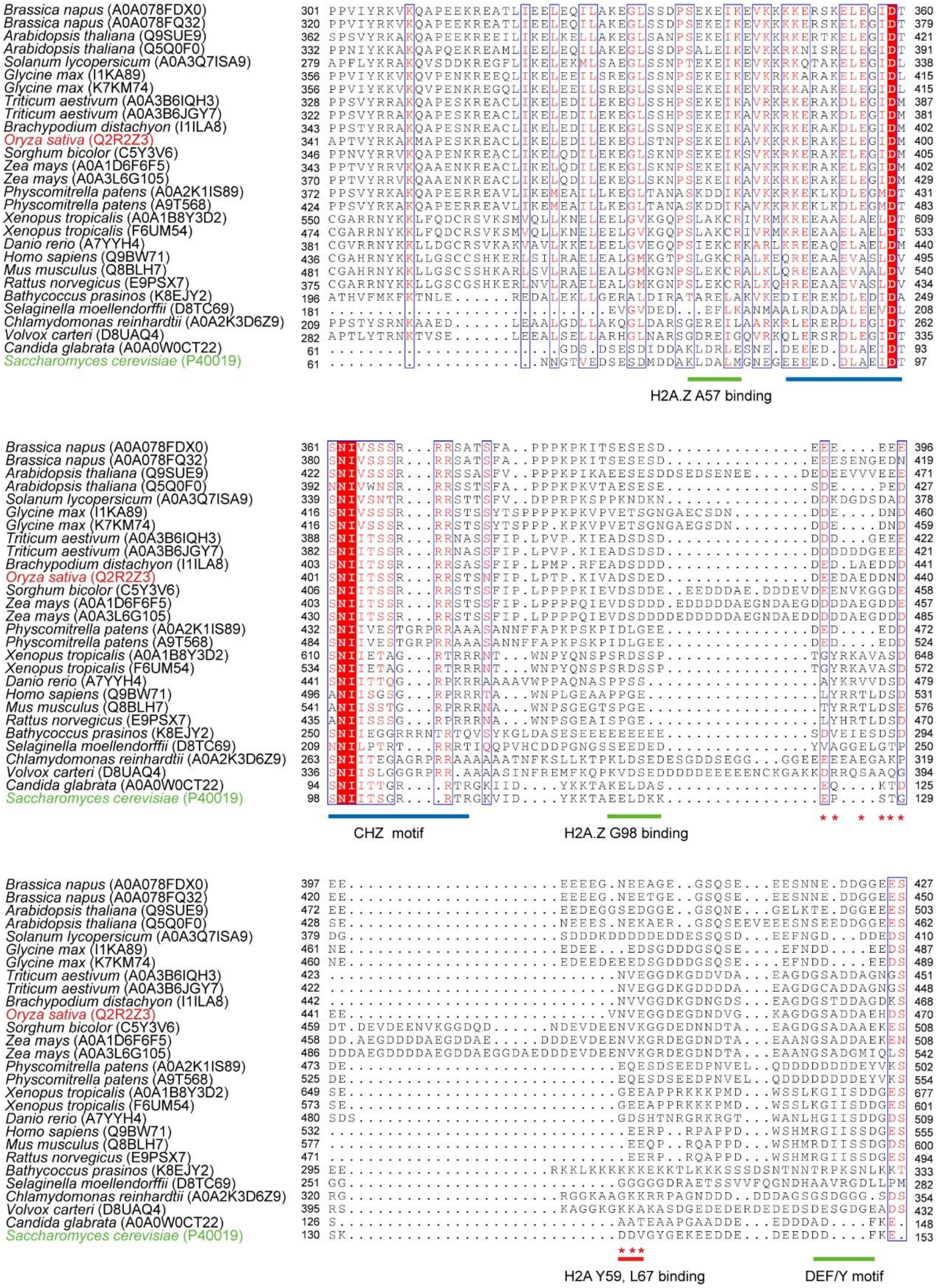

Supplementary Figure 5. Sequence alignments of the C-termini of the Chz1-family proteins from various organisms. The identical residues are highlighted by red background, and the similar residues by blue box. CHZ motif (blue), yChz1 regions involved in H2A.Z-binding (green), and OsChz1 region involved in H2A-binding (red) are underlined. Asterisks in red indicate the residues involved in the interaction with H2A-H2B, as determined by the crystal structure of OsChz1-H2A-H2B in this study.

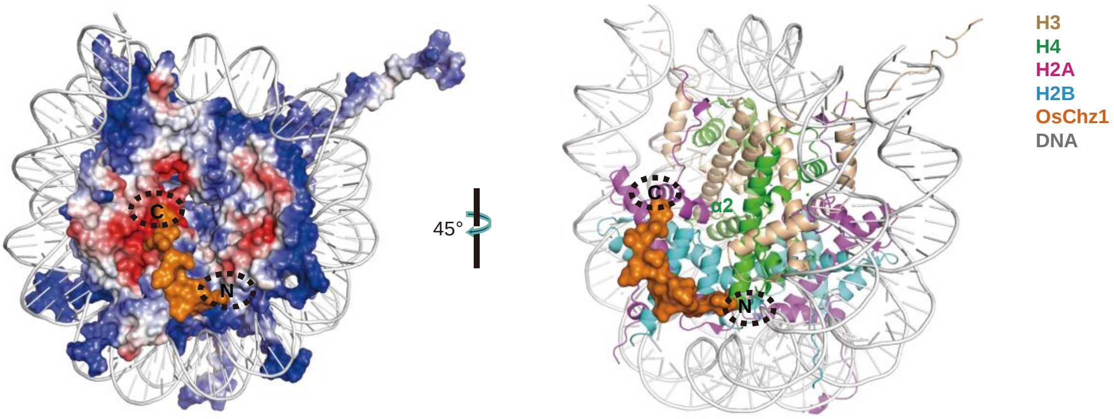

Supplementary Figure 6. Surface (left) and Ribbon (right) representations of nucleosome and globular diagram of OsChz1 (432-446) in a predicted complex. Red and blue in the surface representation denote negative and positive charges, respectively. H3 is colored in brown, H4 in green, H2A in purple, H2B in blue, OsChz1 in orange and DNA in gray. The dash-line-circled N and C indicate the N-terminus and C-terminus of OsChz1 (432-446), respectively. The helix α2 of histone H4 is indicated in the Ribbon representation.

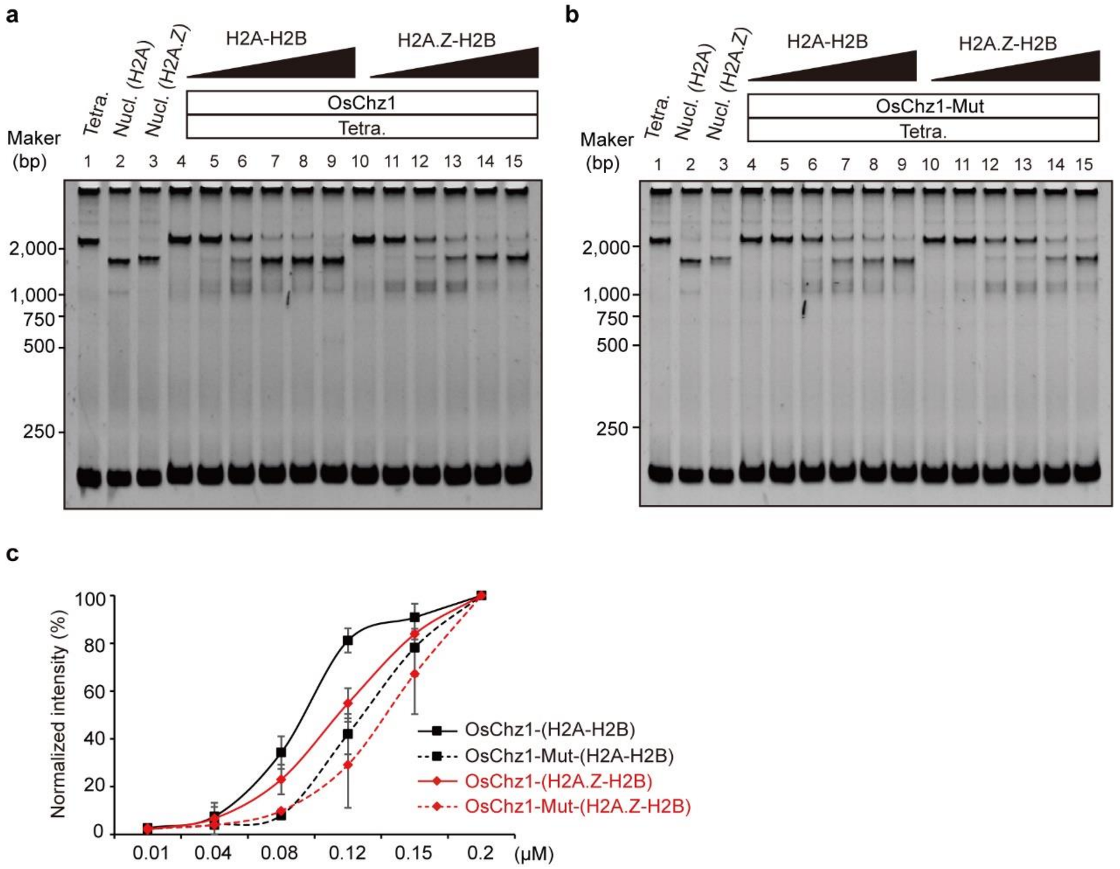

Supplementary Figure 7. Nucleosome assembly assay for histone chaperone activity of wild-type and mutated (Mut) OsChz1 recombinant proteins. a and **b** Native PAGE gels showing assembly of the H3-H4-DNA tetrasome (Tetra.) into nucleosome (Nucl.) upon reaction with increasing amounts of either H2A-H2B or H2A.Z-H2B, as indicated, in the presence of OsChz1 and OsChz1-Mut, respectively. **c** Line chart showing intensity quantifications of nucleosome assembly. The x-axis indicates the increasing amounts of the H2A-H2B dimer or the H2A.Z-H2B dimer. Black curves represent H2A-H2B deposition and red curves represent H2A.Z-H2B deposition. Solid lines represent histone deposition with OsChz1 (0.2 μM) and dashed lines represent histone deposition with OsChz1-Mut (0.2 μM). Highest values, e.g. lane 9 for H2A-H2B and lane 15 for H2A.Z-H2B shown in (a, b), were set as 100%. The data shown are means ± standard deviation (SD) from n = 3 independent biological replicates. Experiments were repeated independently three times with similar results. Source data are provided as a Source Data file.

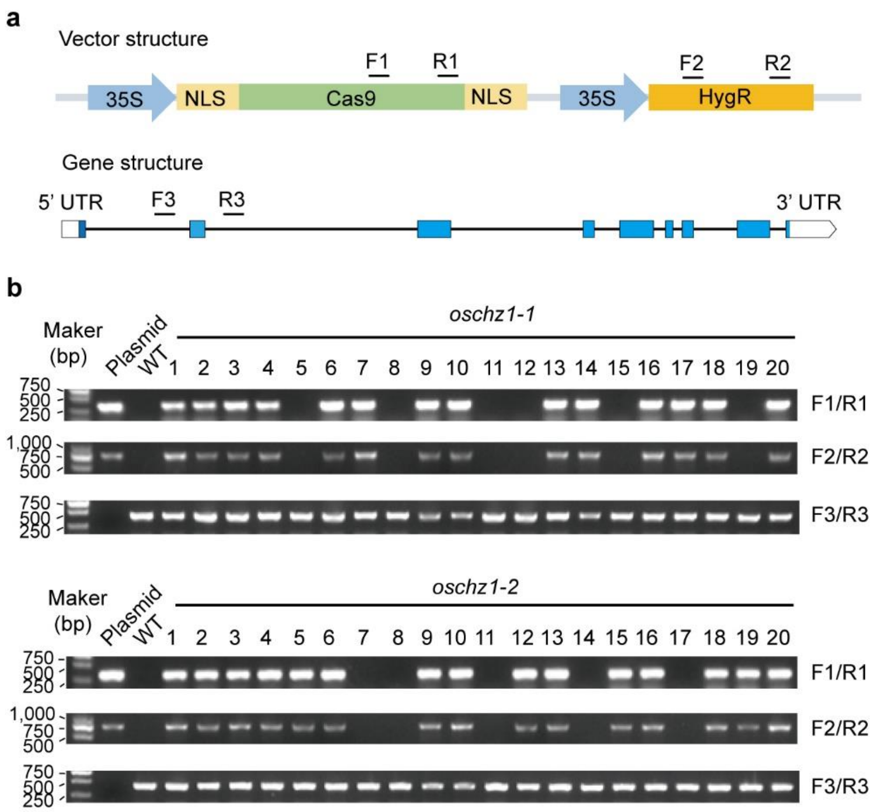

Supplementary Figure 8. Identification of transgene-free *oschz1-1* and *oschz1-2* mutants generated by using CRISPR/Cas9 technology. a Schematic diagram of the vector and the *OsChz1* gene structure. Two pairs of the forward and reverse PCR primers (F1/R1 and F2/R2) were used to detect transgene, one pair (F3/R3) to detect *OsChz1* serving as a DNA quality control. **b** Agarose gel analyses of PCR products using as templates: plasmid (positive control for F1/R1 and F2/R2), wild-type rice genomic DNA (WT, positive control for F3/R3), and genomic DNA prepared from 20 individual F3-generation plants of the *oschz1-1* and *oschz1-2* mutant lines (lanes 1 to 20). Note for the absence of F1/R1 and F2/R2 products in lanes 5, 8, 11, 12, 15, 19 for *oschz1-1* and lanes 7, 8, 11, 14, 17 for *oschz1-2*, which correspond to purified transgene-free mutant plants. Experiments were repeated independently twice with similar results. Source data underlying Supplementary Figure 8b are provided as a Source Data file.

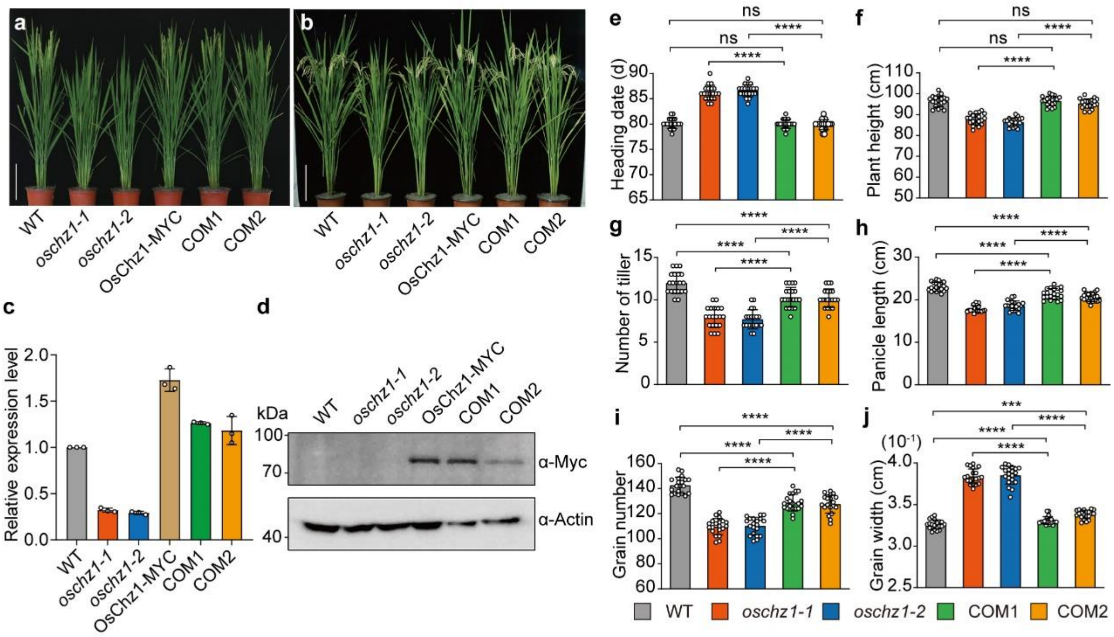

Supplementary Figure 9. Rescue of the *oschz1* mutants by complementation with an *OsChz1* transgene. a and **b** Representative images of plants at heading date stage grown under long-day (LD) and short-day (SD) photoperiods, respectively. The wild-type (WT), the *oschz1-1* and *oschz1-2* mutants, the transgenic OsChz1-MYC, and the mutants complemented with the *pOsChz1::OsChz1-4×MYC* transgene (COM1 and COM2) were grown side-by-side under the same condition. Scale bar = 20 cm. **c** Relative expression levels of *OsChz1* in WT, *oschz1-1*, *oschz1-2*, OsChz1-MYC, COM1 and COM2. Values shown are the mean ± standard deviation from three independent biological replicates after normalization with the internal control *Ubiquitin5*. **d** Western blot detection of the OsChz1-MYC protein in the different plant lines. Actin served as an internal loading control. Experiments were repeated independently twice with similar results. **e-j** Quantitative assessments of agronomic traits for heading date, plant height, number of tillers, panicle length, grain number and grain width, respectively. Values are shown as means ± SD of 20 individual plants. Statistical significance was determined by two-tailed, paired Student's *t*-test: ****, $p < 0.0001$; ns (not significant), $p > 0.05$. The exact *p*-values are 0.7381 (WT vs. COM1), 9.86×10^{-19} (*oschz1-1* vs. COM1), 0.4239 (WT vs. COM2), 1.97×10^{-19} (*oschz1-2* vs. COM2) for **e**; 0.8499 (WT vs. COM1), 1.49×10^{-13} (*oschz1-1* vs. COM1), 0.1849 (WT vs. COM2), 9.37×10^{-16} (*oschz1-2* vs. COM2) for **f**; 7.69×10^{-5} (WT vs. COM1), 3.34×10^{-7} (*oschz1-1* vs. COM1), 2.39×10^{-5} (WT vs. COM2), 1.93×10^{-8} (*oschz1-2* vs. COM2) for **g**; 3.75×10^{-5} (WT vs. COM1), 5.40×10^{-7} (*oschz1-1* vs. COM1), 8.15×10^{-5} (WT vs. COM2), 4.56×10^{-7} (*oschz1-2* vs. COM2) for **h**; 5.87×10^{-8} (WT vs. COM1), 3.12×10^{-11} (*oschz1-1* vs. COM1), 3.23×10^{-8} (WT vs. COM2), 1.71×10^{-9} (*oschz1-2* vs. COM2) for **i**; 0.0049 (WT vs. COM1), 3.55×10^{-24} (*oschz1-1* vs. COM1), 1.33×10^{-8} (WT vs. COM2), 5.31×10^{-20} (*oschz1-2* vs. COM2) for **j**. Source data underlying Supplementary Figure 9c-j are provided as a Source Data file.

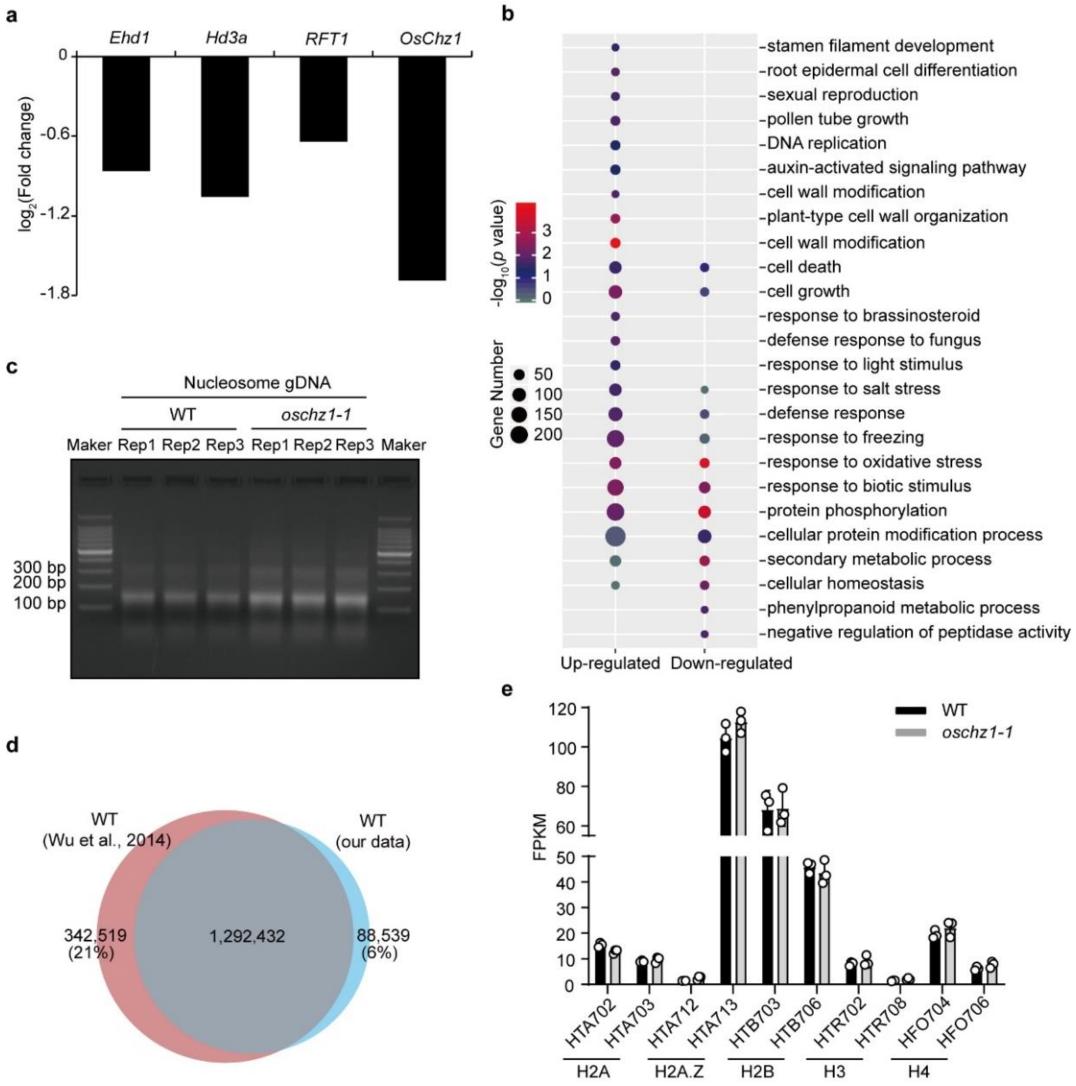

Supplementary Figure 10. Effects of loss-of-OsChz1 on transcription and quality control of MNase-seq analysis. a Down-regulation of *Ehd1*, *Hd3a*, *RFT1* and *OsChz1* in the *oschz1-1* mutant revealed from RNA-seq. **b** GO analysis of the up- and down-regulated genes identified by RNA-seq in *oschz1-1*. Increasing sizes per point represent higher numbers of genes belonging to the indicated functional categories, and varied colors depict *p*-values for the statistical significance of enrichment. **c** Agarose gel electrophoresis analysis of MNase-digested genomic DNA fragments from WT and *oschz1-1*, each with three independent biological replicates (Rep1, Rep2 and Rep3). Experiments were repeated independently three times with similar results. **d** Overlap of the nucleosome peaks detected in this study (green circle) and those published previously (red circle) in WT. **e** Comparison of expression levels (FPKM) of histone genes in WT (black) and in *oschz1-1* (gray) from RNA-seq analysis. Values shown are the mean ± SD from n = 3 independent biological replicates. Differences between WT and *oschz1-1* were not significant statistically, as determined by two-tailed, paired Student's *t*-test ($p > 0.05$). The exact *p*-values are 0.0207, 0.3728, 0.0544, 0.1901, 0.9483, 0.4852, 0.4829, 0.0551, 0.2817, 0.1329 for the genes listed from the left to the right of x-axis. Source data underlying Figure 10a, 10c, and 10e are provided as a Source Data file.

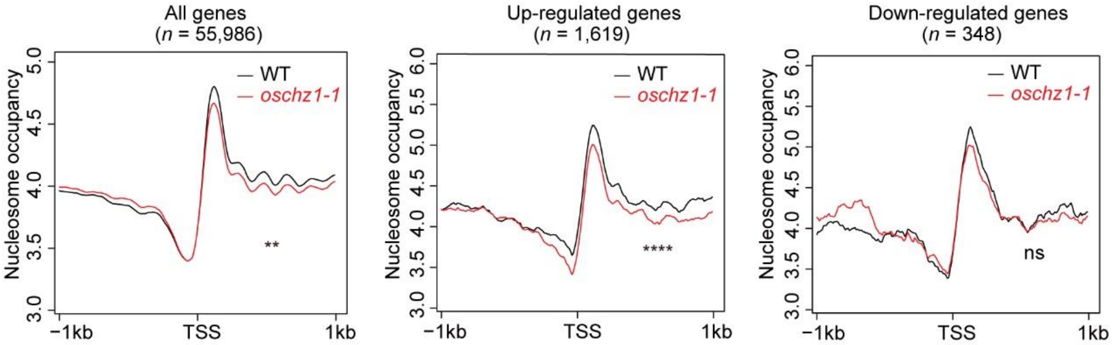

Supplementary Figure 11. Density plots showing nucleosome occupancy of all genes and DEGs identified in the *oschz1-1* mutant. The plots were generated from 1 kb upstream to 1 kb downstream of TSS, as determined by MNase-seq. Each profile represents a merge from two biological replicates. The statistically significant differences between wild-type (WT) and *oschz1-1* were determined by two-tailed Welch Two Sample *t*-test: **, $p = 0.0097$; ****, $p = 6.83 \times 10^{-23}$; ns (not significant), $p = 0.1938$.

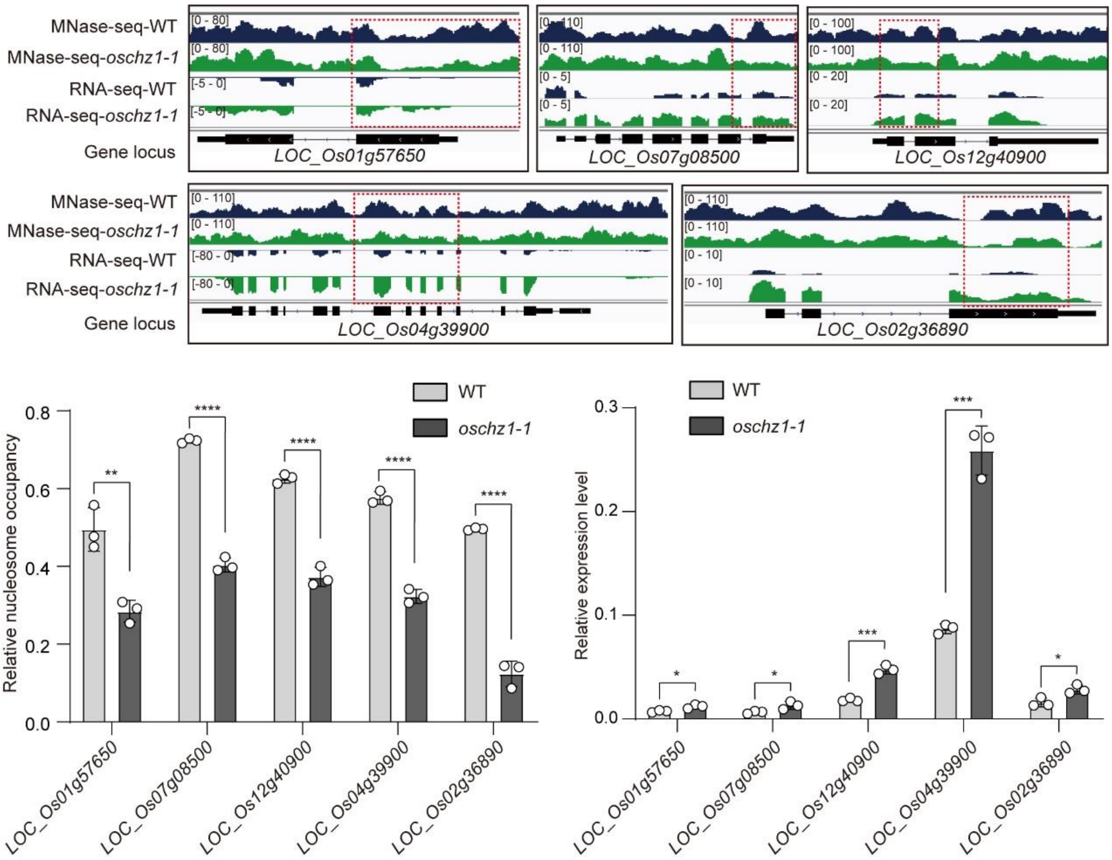

Supplementary Figure 12. Validation by quantitative PCR of randomly selected genes, which displayed a reduction of nucleosome occupancy and an increase of transcription in *oschz1-1* in genome-wide profiling analyses. The top panels show plots from profiling data, with red-dot-boxes pointing to the regions analyzed by qPCR. The bottom-left graph shows qPCR results on nucleosome occupancy, and the bottom-right graph shows qPCR results on gene expression. Value represents mean ± SD from n = 3 independent biological replicates. The statistically significant differences between wild-type (WT) and *oschz1-1* were determined by two-tailed, paired Student's *t*-test: *, $p < 0.05$; **, $p < 0.01$; ***, $p < 0.001$; ****, $p < 0.0001$. The exact *p*-values for the genes listed from the left to the right of x-axis are 0.0044, 9.73×10^{-8}, 9.99×10^{-5}, 5.08×10^{-5}, 3.97×10^{-5} (bottom-left graph); 0.0187, 0.0457, 0.0004, 0.0002, 0.0231(bottom-right graph). Source data are provided as a Source Data file.

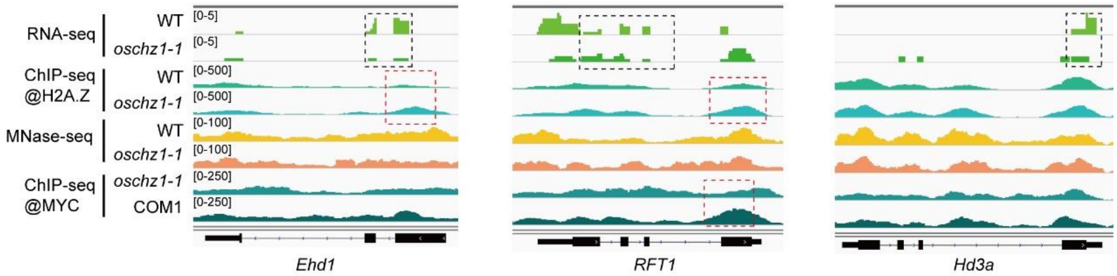

Supplementary Figure 13. Profile plots of the flowering genes *Ehd1*, *RFT1* and *Hd3a* from RNA-seq, MNase-seq and ChIP-seq analyses. Obvious differences observed between *oschz1* mutant and wild-type (WT) or COM1 are highlighted with dot boxes in different colors, dark for down and red for up.

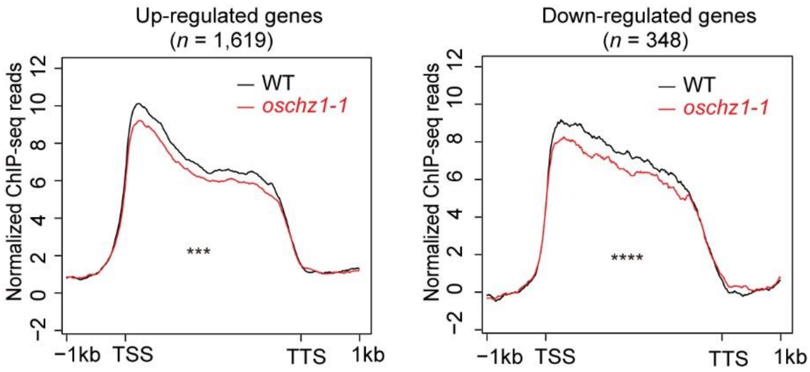

Supplementary Figure 14. Comparison of H2A.Z occupancy in wild-type (WT) and in the *oschz1-1* mutant, at the up-regulated and down-regulated genes identified in the *oschz1-1* mutant. The plots were generated from 1 kb upstream of TSS to 1 kb downstream of TTS determined by ChIP-seq. Signal for H2A.Z is a merge of two biological replicates. The statistically significant differences between wild-type (WT) and *oschz1-1* were determined by single-end Welch Two Sample t-test: ***, $p = 0.0003$; ****, $p = 1.97 \times 10^{-5}$.

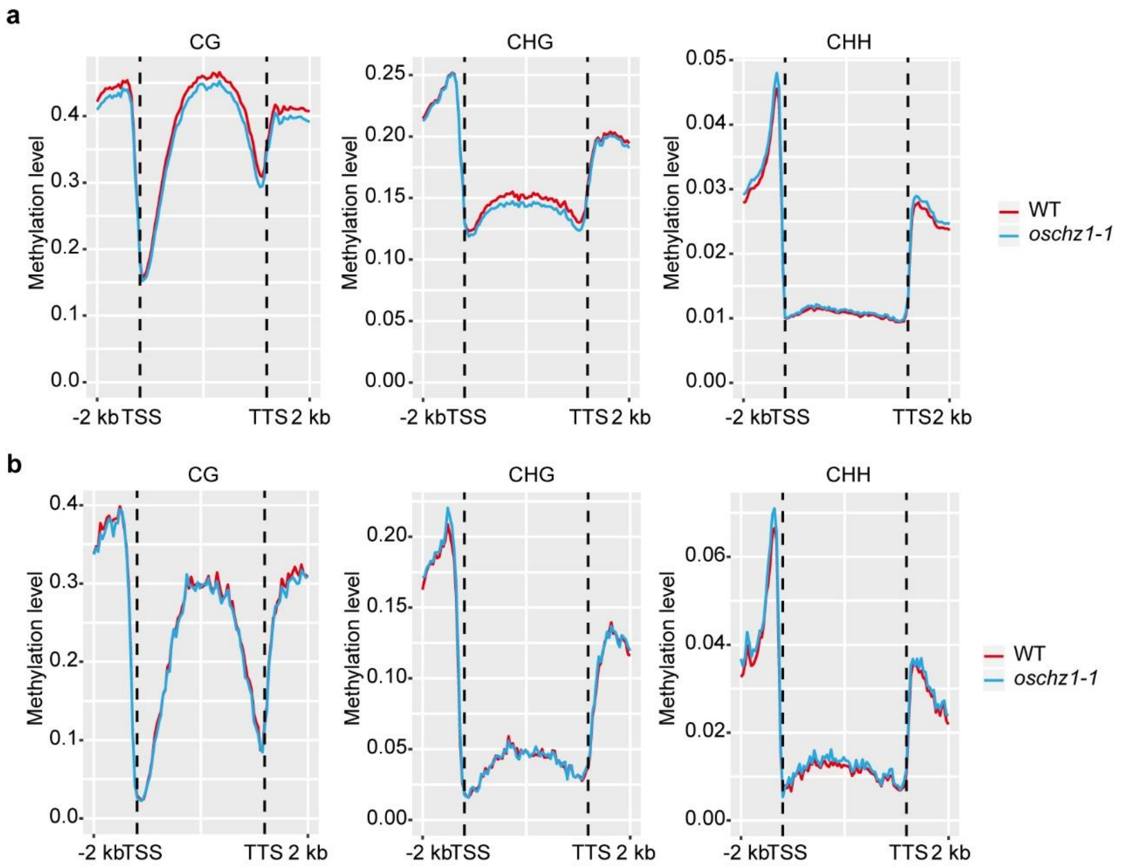

Supplementary Figure 15. Comparison of DNA methylation patterns in wild-type (WT) and in the *oschz1-1* mutant. a Average distribution of DNA methylation levels at all genes, in the CG, CHG and CHH contexts as indicated. **b** Average distribution of DNA methylation levels at genes containing decreased H2A.Z levels in the mutant, in the CG, CHG, and CHH contexts as indicated. Genome-wide DNA methylation data were obtained by BS-seq in this study.

Supplementary Table 1. Data collection and refinement statistics in crystal structure analysis.

OsChz1-H2A-H2B	
Data collection	
Space group	C121
Cell parameter	
a (Å)	167.3
b (Å)	116.3
c (Å)	101.9
α (°)	90.0
β (°)	114.9
γ (°)	90.0
Wavelength(Å)	0.97915
Resolution (Å)	30.0-2.85
Last shell (Å)	2.95-2.85
Completeness (%)	98.3(99.2)
Redundancy	2.8(3.2)
I/□ (I)	10.1(2.0)
Rmerge (%)	7.3(46.7)
Refinement	
Resolution (Å)	30.0-2.85
R_{work} (%) / R_{free} (%)	19.4/25.3
No. of atoms	
Protein	8000
Ligand/H_2O	15/14
R.m.s. deviations	
Bond length (Å)	0.014
Bond angle (°)	1.738
Ramachandran plot (%)	
Most favored	97.11
Additional allowed	2.89
PDB number	6M2M

Values in parentheses are for the last resolution shell.

Supplementary Table 2. Mutagenesis of recombinant OsChz1 protein.

Name	Amino acid substitution
OsChz1-C-1M	E432A, E433A
OsChz1-C-2M	E436A, D438A, N439A, D440A
OsChz1-C-3M	N444A, V445A, E446A
OsChz1-Mut	E432A, E433A, E436A, D438A, N439A, D440A, N444A, V445A, E446A

Supplementary Table 3. Analysis of potential off-target sites of *OsChz1*- CRISPR-P in the rice genome.

Off-target	Sequence (5'-3')	Score	Genomic locus (Chromosome:start)	Region (gene)	Mutation
1	CCAAGGTACAAGAA GAGCACAGG	0.590	6:-7548279	Intergenic	N.D.
2	AGAGGGGGCAAGGA GAGCACCGG	0.339	8:+5462300	Intergenic	N.D.
3	TTTAGGTGGAAGGA GAGCACCGG	0.278	2:-20140416	Os02g0542200	N.D.
4	AGAAGCTTTAAGGA GAGTACTGG	0.274	4:+6250491	Os04g0191600	N.D.
5	CGGAGGTGTAGGAA GAGAACGGG	0.197	6:-10803160	Intergenic	N.D.

N.D., not detected.

Supplementary Table 4. Analysis of nucleosome distribution in wild-type (WT) and *oschz1-1*.

		Gene body	promoter	Intergenic region
WT	Nucleosome count	631058	193209	592483
	%[a]	45.18%	13.83%	42.42%
Increased occupancy in *oschz1-1*	Nucleosome count	6551	2482	8050
	%[b]	38.74%	14.68%	47.60%
	p-value (Increased freq.)	1	0.0008	9.25E-43
Decreased occupancy in *oschz1-1*	Nucleosome count	7135	2076	6410
	%[b]	46.26%	13.46%	41.56%
	p-value (Increased freq.)	0.0035	0.9137	0.9854
Forward shift in *oschz1-1*	Nucleosome count	2565	768	2627
	%[b]	43.50%	13.03%	44.56%
	p-value (Increased freq.)	0.9954	0.9662	0.0005
Reverse shift in *oschz1-1*	Nucleosome count	2568	777	2599
	%[b]	43.75%	13.24%	44.28%
	p-value (Increased freq.)	0.9869	0.9111	0.0021

[a] percentage of detected nucleosomes

[b] percentage of dynamic nucleosomes. Significance level of the intersection was calculated by single-end Fisher's exact test.

Supplementary Table 5. Primers used in this study.

Primer name	Forward primer 5'-3'	Reverse primer 5'-3'	Usage
OsChz1-KO	ATTTTCACGCAGGCAGGAGA	CTCCCAGGTTCATACAGCAA	genotyping
Hygromycin	ACTATCGGCGAGTACTTCTACAC	GTGCTTGACATTGGGGAGTTTAG	genotyping
Cas9	ACGGAAAGTAAGGATCTTCTCGA	CTGAGATCACTAAGGCTCCTCTT	genotyping
UBQ	TATCATCGAGCCGTCGCTTC	TGGTTGCTGTGACCACACTT	qRT-PCR
OsChz1	GATGAGCCGGTGGCAAAATC	GTCCGGCGAACTCCTTGTAA	qRT-PCR
Os01g57650	TGAGCAGTTCAGCAAGTGGG	CTTGAACACACGCTCGATCC	qRT-PCR
Os07g08500	GTGGACCTGATCCCTTGGTG	CTGAGATCGCGCACATTCAC	qRT-PCR
Os12g40900	GGACGGAGCTCCATACCTGA	GTCCTTGTCCTCGTAGGTGAC	qRT-PCR
Os04g39900	AGGAGTACGCTGAAACCTGC	CTCCCTTCCTGAATCGCCAG	qRT-PCR
Os02g36890	CCGGTGGACGAACTACCTTC	CCTCTTCTTCAGGTGCGTGT	qRT-PCR
Hd3a	AGCCCAAGTGACCCTAACCT	GTTGTAGAGCTCGGCGAAGT	qRT-PCR
RFT1	ACCCTAACCTTAGGGAGTATCTACAC	GCCTGCATGCATATACAGCTAGGCAG	qRT-PCR
Ehd1	GTTGCCAGTCATCTGCAGAA	GGATGTGGATCATGAGACAT	qRT-PCR
Ehd3	AAGACAAGGATGATGACCAG	TACCATCACCTTCAGAATCC	qRT-PCR
Ehd4	GATGGAGCAAAGTTGTGGAA	GCATGTGGATAAAGCAATGG	qRT-PCR
Ghd7	CCGATGATGGGGAGAGCTTG	CCTCATCTCGGCATAGGCTT	qRT-PCR
OsGI	GTGGATGCGCTTTGTGACAT	GGCCTGCAGAACGATAGCA	qRT-PCR
Hd1	AACCAAGATCGGCAGTATGG	GATTGATTGCTCCAGCAGGT	qRT-PCR
OsMADS50	GTCGCGCTCATCGTCTTCTC	TGCCAAACCATCAGCGTCAG	qRT-PCR
OsMADS51	GCTTAGGGACATAACGGCCT	CTGGAGTTTGCTCTGCTCCT	qRT-PCR
OsMADS56	TGGAGCAGCAGATAGCCAAG	GTCGTCGTCATGTGGTTAGC	qRT-PCR
Os01g57650	TGAGCAGTTCAGCAAGTGGG	CTTGAACACACGCTCGATCC	qRT-PCR
Os07g08500	GTGGACCTGATCCCTTGGTG	CTGAGATCGCGCACATTCAC	qRT-PCR
Os12g40900	GGACGGAGCTCCATACCTGA	GTCCTTGTCCTCGTAGGTGAC	qRT-PCR
Os04g39900	AGGAGTACGCTGAAACCTGC	CTCCCTTCCTGAATCGCCAG	qRT-PCR
Os02g36890	CCGGTGGACGAACTACCTTC	CCTCTTCTTCAGGTGCGTGT	qRT-PCR
off-target-1	ATGATGACGAGGAGGTTGCT	TTCATGTGAGACAGTGGCCA	gPCR
off-target-2	TCCAACACCGACCAAATTGT	AGTTCCATCCTCGTCGATCC	gPCR
off-target-3	GAGAGTGGCATATCGTCCCT	TAGGGCCGTACAAAGCGAAT	gPCR
off-target-4	CAGCAACTTGGAACCAGCAT	GTCCATGACTTCAGCCTGTG	gPCR
off-target-5	ACGAACGATCAAACATGTGCT	CAGAGACATTTGCACCTCGG	gPCR
OsChz1-CDS	CGGAATTCATGGAGCCCGACGCCGCAGC	CCGTCGACTCAATCACTGTCGTGCTCAGCATC	construction
OsChz1-C-1M	AAGATGACGCAGCAGACGCAGAAGATGACAATG	TGCGTCTGCTGCGTCATCTTCATCACTATCA	construction
OsChz1-C-2M	ACGCAGCAGATGCCGCTGCTGAGGAGGTGAATGTGGAGG	CTCAGCAGCGGCATCTGCTGCGTCTTCTTCGTCATC	construction
OsChz1-C-3	GAGGTGGCTGCGGCGGGTGGAGATG	CTCCACCCGCCGCAGCCACCTCCTCATC	construction

M	AAGGTGACAAT	ATTGTCAT	
OsChz1(1-78)	CGGAATTCATGGAGCCCGACGCCGCAGC	GCGTCGACCTAATTGTCATCATCAGAACCAG	construction
OsChz1(79-337)	GCGAATTCATGACGAATAATAATGCACCAGAG	GCGTCGACCTATCCACAAGATTTGATTATTGAC	construction
OsChz1(338-471)	GCGAATTCATGAGTATCGCACCTACTGTGT	CCGTCGACTCAATCACTGTCGTGCTCAGCATC	construction
OsChz1(390-422)	CGGAATTCATGGAAAGAGCAAAGGAACTCGAG	CGGTCGACCTATTTAGGCGTTGGCAGGGGTA	construction
p1391z-OsChz1	TTGGCTGCAGGTCGACCGTTGCAGAGTAGCACGGTCT	CCGGGGATCCGTCGACGCGGATGCGGCGGCGGCG	construction
HTA702-MYC	CGGGATCCATGGCTGGTAGGGGCAAGGC	CGGGTACCCTCGTCGTCGGCGGCGGC	construction
HTA713-HA	CGGGATCCATGGCGGGAAAAGGAGGTAAG	CCCAAGCTTCTCTTTGGAGGACTTGTTGAT	construction
GFP-OsChz1	CGGGATCCATGGAGCCCGACGCCGCAGC	CGGGTACCTCAATCACTGTCGTGCTCAGCATC	construction
601DNA	CTCGGGTGATGCCGGATCCCCT	CAAGCGACACCGGCACTGGAAC	PCR
Os01g57650	CTTCGGATGCCGCTATTGCT	GCGCGGTGCTAGCCACTA	validation
Os07g08500	AGCAAGCACAGGCAGATTGG	AGGACTCTGAGCACCTACTGA	validation
Os12g40900	CATGGATGGCAGAGCTAGCT	CGGCTTGCTCTTCGTCGT	validation
Os04g39900	CAGGAAGGGAGCCTTACACC	AACGGATTCTTTTGGTGACCTG	validation
Os02g36890	CGTGCAGTCATCATACGGGG	GACGGAGCAGTCAAGAAGGA	validation
