# Supplementary material for: OsChz1 acts as a histone chaperone in modulating chromatin organization and genome function in rice
Source: Nat Commun. 2020 Nov 11;11:5717. doi: 10.1038/s41467-020-19586-z (PMC7658359; doi:10.1038/s41467-020-19586-z)
Supplement: Supplementary file 3 — Reporting Summary [file 41467_2020_19586_MOESM3_ESM.pdf]

nature research

Corresponding author(s): Wen-Hui Shen

Last updated by author(s): Oct 20, 2020

Reporting Summary

Nature Research wishes to improve the reproducibility of the work that we publish. This form provides structure for consistency and transparency in reporting. For further information on Nature Research policies, see Authors & Referees and the Editorial Policy Checklist .

Statistics

For all statistical analyses, confirm that the following items are present in the figure legend, table legend, main text, or Methods section.

n/a	Confirmed	
☐	☒	The exact sample size (*n*) for each experimental group/condition, given as a discrete number and unit of measurement
☐	☒	A statement on whether measurements were taken from distinct samples or whether the same sample was measured repeatedly
☐	☒	The statistical test(s) used AND whether they are one- or two-sided<br>*Only common tests should be described solely by name; describe more complex techniques in the Methods section.*
☒	☐	A description of all covariates tested
☒	☐	A description of any assumptions or corrections, such as tests of normality and adjustment for multiple comparisons
☐	☒	A full description of the statistical parameters including central tendency (e.g. means) or other basic estimates (e.g. regression coefficient) AND variation (e.g. standard deviation) or associated estimates of uncertainty (e.g. confidence intervals)
☐	☒	For null hypothesis testing, the test statistic (e.g. *F*, *t*, *r*) with confidence intervals, effect sizes, degrees of freedom and *P* value noted<br>*Give P values as exact values whenever suitable.*
☒	☐	For Bayesian analysis, information on the choice of priors and Markov chain Monte Carlo settings
☒	☐	For hierarchical and complex designs, identification of the appropriate level for tests and full reporting of outcomes
☒	☐	Estimates of effect sizes (e.g. Cohen's *d*, Pearson's *r*), indicating how they were calculated

Our web collection on statistics for biologists contains articles on many of the points above.

Software and code

Policy information about availability of computer code

Data collection	For RT-qPCR and ChIP-qPCR, data were collected with CFX ConnectTM Real-Time System (BIO-RAD); RNA-seq, ChIP-seq and MNase-seq data were collected using Illlumina HiSeq3000 instrument via the custom service of GENERGY BIO (Shanghai, China). Images of gels or blots were collected by Clinx Science Instruments (Shanghai, China). Confocal images were acquired with ZEN 2010 software (version 1.0.3846.26637). ITC data were collected by MicroCal iTC200 (GE Healthcare). X-ray diffraction data were collected on beamline BL17U1 and BL19U1 at Shanghai Synchrotron Radiation Facility.
Data analysis	Neighbor-Joining phylogeny based on full-length amino acid sequence alignment was calculated using MEGA (v10.1.8) and illustrated using FigTree v1.4.3; Publicly available computational tools for analyzing RNA-seq, ChIP-seq and BS-seq data were used as described in the Methods section and they include: FastQC (v0.11.7), CUTADAPT (v1.10), HISAT2 (v2.1.0), Samtools (v1.9), FeatureCounts (v1.6.2), DEseq2 (1.22.2), Bowtie2 (v2.3.4.1), v2.25.0, deepTools (v3.0.2), DiffBind (v2.1.0), ChIPpeakAnno (v3.16.1), ChIPseeker (v1.18.0), DANPOS (v2.1.3), SICER (v1.1), Bismark (v0.22.1), ViewBS (v0.1.9). Further information including commands parameters used are provided in Methods section. For protein structure analysis, Phaser (v7.0.076), COOT (v0.8.71), PHENIX (v1.12-2829-000), Refmac5 (v7.0.076) and PyMOL (v2.0) were used. ITC data were analyzed using Origin software v7.0. Results (RT-qPCR, ChIP-qPCR, statistics for agronomic traits) presented as mean ± SD were analyzed by Microsoft Excel 2019 and GraphPad Prism 8.

For manuscripts utilizing custom algorithms or software that are central to the research but not yet described in published literature, software must be made available to editors/reviewers. We strongly encourage code deposition in a community repository (e.g. GitHub). See the Nature Research guidelines for submitting code & software for further information.

Data

Policy information about availability of data

All manuscripts must include a data availability statement. This statement should provide the following information, where applicable:

- Accession codes, unique identifiers, or web links for publicly available datasets
- A list of figures that have associated raw data
- A description of any restrictions on data availability

The authors declare that all data supporting the findings of this work are available within the paper and its Supplementary Information files, and that they are available from the corresponding author upon reasonable request. The RNA-seq, ChIP-seq, MNase-seq and BS-seq data that support the findings of this study have been deposited to NCBI GEO with the accession number GSE155269 (https://www.ncbi.nlm.nih.gov/geo/query/acc.cgi?acc=GSE155269). Structural factors and coordinates have been deposited in the Protein Data Bank (PDB) with accession code 6M2M (https://www.rcsb.org/structure/6M2M) for the OsChz1-H2A-H2B complex. The source data underlying Figures 1b, 2a, c-g, 3d, 4f-j, 5c, d, 6a, d, f, g, 7b, c, d, f, 8a, as well as Supplementary Figures 4, 7, 8b, 9c-j, 10a, c, e, 12 are provided as a Source Data file.

Field-specific reporting

Please select the one below that is the best fit for your research. If you are not sure, read the appropriate sections before making your selection.

[x] Life sciences [ ] Behavioural & social sciences [ ] Ecological, evolutionary & environmental sciences

For a reference copy of the document with all sections, see nature.com/documents/nr-reporting-summary-flat.pdf

Life sciences study design

All studies must disclose on these points even when the disclosure is negative.

Sample size	No statistical methods were used to predetermine sample size for plant experiments. A minimum of 20 individual plants for each genotype were counted based on the previous publication (Liu et al., 2019) describing the sample size for agronomic-trait measurement. The sample sizes for RNA extraction and ChIP assays were selected following the description in Liu et al., 2017. The sample sizes for protoplast isolation and transformation were selected based on established procedure (He et al., 2018). A detailed description of sample sizes for each experiment is given in the Figure legends or Methods section.
Data exclusions	No data was excluded from analysis in this study.
Replication	For H2A.Z, H3 and OsChz1-Myc ChIP-seq experiments, 2 replicates were performed along with DNA input as ChIP-seq control. For RNA-seq, RT-PCR and ChIP-PCR, three replicates were performed as indicated in the figure legends. For MNase-seq and BS-seq, 2 replicates were performed. Flowering time experiments, GST pull-down assay, Co-IP and ITC experiments were replicated independently at least twice with similar results. All statistical experiments were performed in at least three biological replicates to allow for calculation of statistical significance.
Randomization	All normal growing samples were selected randomly.
Blinding	Blinding is not relevant to this study. All experiments were assigned into groups including relevant controls and analysis was done objectively and without bias.

Reporting for specific materials, systems and methods

We require information from authors about some types of materials, experimental systems and methods used in many studies. Here, indicate whether each material, system or method listed is relevant to your study. If you are not sure if a list item applies to your research, read the appropriate section before selecting a response.

Materials & experimental systems

n/a	Involved in the study	
[ ]	[x]	Antibodies
[x]	[ ]	Eukaryotic cell lines
[x]	[ ]	Palaeontology
[x]	[ ]	Animals and other organisms
[x]	[ ]	Human research participants
[x]	[ ]	Clinical data

Methods

n/a	Involved in the study	
[ ]	[x]	ChIP-seq
[x]	[ ]	Flow cytometry
[x]	[ ]	MRI-based neuroimaging

Antibodies

Antibodies used	The immunoprecipitated fractions were detected by western blotting with anti-HA (ab9110, Abcam, 1:2000 dilution), anti-MYC

(M20002L, Abmart, 1:5000 dilution) or anti-GFP antibodies (M20004L Abmart, 1:5000 dilution for WB and 1:100 dilution for IP); anti-Actin (M20009L, Abmart, 1:5000 dilution); Secondary antibodies were goat anti-rabbit IgG-HRP (M21002L, Abmart, 1:5000 dilution) and goat anti-mouse IgG-HRP (M21001L, Abmart, 1:5000 dilution) ; Antibodies used in ChIP were as follows: anti-MYC (M20002L, Abmart, 1:300 dilution), anti-H3 (ab1791, Abcam, 1:300 dilution) and anti-H2A.Z (Li et al., J. Integr. Plant Biol, 2018, 1:100 dilution).

Validation

All validation statements, including citation for commercial antibodies can be found on the manufacturers' websites:
Anti-HA: (http://www.abcam.cn/ha-tag-antibody-chip-grade-ab9110.html)
Anti-MYC: (http://www.ab-mart.com.cn/upload/20170614093526xz.pdf)
Anti-GFP: (http://www.ab-mart.com.cn/upload/20170614093556xz.pdf)
Anti-Actin: (http://www.ab-mart.com.cn/upload/20170614135558xz.pdf)
Anti-H3: (http://www.abcam.cn/histone-h3-antibody-nuclear-marker-and-chip-grade-ab1791.html)
The antibody against H2A.Z was validated in Li et al., J. Integr. Plant Biol, 2018.

ChIP-seq

Data deposition

- [x] Confirm that both raw and final processed data have been deposited in a public database such as GEO.
- [x] Confirm that you have deposited or provided access to graph files (e.g. BED files) for the called peaks.

Data access links
May remain private before publication.

All generated high-throughput sequencing data that support the findings of this study have been deposited to NCBI GEO with the accession number GSE155269 (https://www.ncbi.nlm.nih.gov/geo/query/acc.cgi?acc=GSE155269). The following secure token has been created to allow review of record GSE155269 while it remains in private status: iejwdoqgohtylfmb.

Files in database submission

NIP_H2AZ_rep1_R1.fastq.gz,NIP_H2AZ_rep2_R1.fastq.gz,NIP_H3_rep1_R1.fastq.gz,NIP_H3_rep2_R1.fastq.gz,NIP_input_rep1_R1.fastq.gz,NIP_input_rep2_R1.fastq.gz,oschz1_H2AZ_rep1_R1.fastq.gz,oschz1_H2AZ_rep2_R1.fastq.gz,oschz1_H3_rep1_R1.fastq.gz,oschz1_H3_rep2_R1.fastq.gz,oschz1_input_rep1_R1.fastq.gz,oschz1_input_rep2_R1.fastq.gz,NIP_H2AZ_rep1_R2.fastq.gz,NIP_H2AZ_rep2_R2.fastq.gz,NIP_H3_rep1_R2.fastq.gz,NIP_H3_rep2_R2.fastq.gz,NIP_input_rep1_R2.fastq.gz,NIP_input_rep2_R2.fastq.gz,oschz1_H2AZ_rep1_R2.fastq.gz,oschz1_H2AZ_rep2_R2.fastq.gz,oschz1_H3_rep1_R2.fastq.gz,oschz1_H3_rep2_R2.fastq.gz,oschz1_input_rep1_R2.fastq.gz,oschz1_input_rep2_R2.fastq.gz,NIP_H2AZ_rep1_RPKM.bigwig,NIP_H2AZ_rep2_RPKM.bigwig,NIP_H3_rep1_RPKM.bigwig,NIP_H3_rep2_RPKM.bigwig,NIP_input_rep1_RPKM.bigwig,NIP_input_rep2_RPKM.bigwig,oschz1_H2AZ_rep1_RPKM.bigwig,oschz1_H2AZ_rep2_RPKM.bigwig,oschz1_H3_rep1_RPKM.bigwig,oschz1_H3_rep2_RPKM.bigwig,oschz1_input_rep1_RPKM.bigwig,oschz1_input_rep2_RPKM.bigwig,com_MYC_rep1_R1.fastq.gz,com_MYC_rep2_R1.fastq.gz,com_input_rep1_R1.fastq.gz,com_input_rep2_R1.fastq.gz,oschz1_MYC_rep1_R1.fastq.gz,oschz1_MYC_rep2_R1.fastq.gz,oschz1_input_rep1_R1.fastq.gz,oschz1_input_rep2_R1.fastq.gz,,com_MYC_rep1_R2.fastq.gz,com_MYC_rep2_R2.fastq.gz,com_input_rep1_R2.fastq.gz,com_input_rep2_R2.fastq.gz,oschz1_MYC_rep1_R2.fastq.gz,oschz1_MYC_rep2_R2.fastq.gz,oschz1_input_rep1_R2.fastq.gz,oschz1_input_rep2_R2.fastq.gz,com_MYC_rep1_RPKM.bigwig,com_MYC_rep2_RPKM.bigwig,com_input_rep1_RPKM.bigwig,com_input_rep2_RPKM.bigwig,oschz1_MYC_rep1_RPKM.bigwig,oschz1_MYC_rep2_RPKM.bigwig,oschz1_input_rep1_RPKM.bigwig,oschz1_input_rep2_RPKM.bigwig

Genome browser session
(e.g. UCSC)

no longer appliable

Methodology

Replicates

We generated two biological replicates for all ChIP-seq experiments.

Sequencing depth

NIP_H2AZ_rep1,44268102,35866055,150bp,paired-end,NIP_H2AZ_rep2,33395234,27343549,150bp,paired end,NIP_H3_rep1,51465160,37432101,150bp,paired-end,NIP_H3_rep2,38824594,28353103,150bp,paired-end,NIP_input_rep1,20823272,13922072,150bp,paired-end,NIP_input_rep2,17037222,11204557,150bp,paired-end,oschz1_H2AZ_rep1,40795182,33221195,150bp,paired-end,oschz1_H2AZ_rep2,37657092,30855427,150bp,paired-end,oschz1_H3_rep1,55735706,40447730,150bp,paired-end,oschz1_H3_rep2,37157136,27060907,150bp,paired-end,oschz1_input_rep1,21137236,13922475,150bp,paired-end,oschz1_input_rep2,19511296,12847805,150bp,paired-end,
com_MYC_rep1,38285274,24885313,150bp,paired-end,com_MYC_rep2,43069622,27866035,150bp,paired-end,com_input_rep1,42540212,27393100,150bp,paired-end,com_input_rep2,61033754,38745975,150bp,paired-end,oschz1_MYC_rep1,29910976,18322280,150bp,paired-end,oschz1_MYC_rep2,37589230,23470682,150bp,paired-end,oschz1_input_rep1,53536484,33699592,150bp,paired-end,oschz1_input_rep2,55492290,34977645,150bp,paired-end,

Antibodies

anti-MYC (M20002L, Abmart, 1:300 dilution), anti-H3 (ab1791, Abcam, 1:300 dilution) and anti-H2A.Z (Li et al., J. Integr. Plant Biol, 2018, 1:100 dilution).

Peak calling parameters

H2A.Z and OsChz1-MYC enrichment regions (peaks) were identified by comparing the ChIP library with input library (parameters: W = 200, G = 200, FDR = 0.05) using SICER.sh from SICER v1.1 software. ChIPpeakAnno package in R was used to intersect peaks in two replicates to produce the final set of enriched peaks for each sample. DiffBind was used to compare two different samples to identify differentially enrichment peaks (FDR ≤ 0.05).

Data quality	We used fastqc to check the sequencing quality of ChIP-seq sequencing data, then use cutadapt to remove reads with adapter and low-quality sequnces.
Software	Bowtie v1.2.2; Samtools v1.9; deepTools v3.0.2; SICER v1.1; MACS14; BED Tools v2.25.0; ChIPseeker v1.18.0; DiffBind v2.1.0.
