# Supplementary material for: OsChz1 acts as a histone chaperone in modulating chromatin organization and genome function in rice
Source: Nat Commun. 2020 Nov 11;11:5717. doi: 10.1038/s41467-020-19586-z (PMC7658359; doi:10.1038/s41467-020-19586-z)
Supplement: Supplementary file 2 — Peer Review [file 41467_2020_19586_MOESM2_ESM.pdf]

Reviewer #1 (Remarks to the Author):

This is an interesting and comprehensive study characterizing the rice homologue of yeast Chz involved in H2A.Z deposition. This study combines structural data on OsChz binding to H2A-H2B dimers with biochemical studies, analysis of a rice mutant for the unique Chz gene in the rice genome and genome wide studies of nucleosomal occupancy, H2A.Z and OsChz enrichment and gene expression. This study is therefore original and rather complete and brings novel insight into the function of a CHZ-domain protein in plants.

Several points need to be further considered:

In Figure 2 the authors have divided OsChz in 3 non-overlapping protein regions to study interaction with H2A-H2B and H2A.Z-H2B (N 1-78, M 79-337 and C 338-471, the latter comprising the Chz domain). SFig4 shows that both M and C regions of OsChz are able to bind histones with Chz-C preferentially binding H2A over H2A.Z, while this is not the case for the middle region. This suggests that Chz may interact with histones in different ways, implicating different regions of the Chz protein. The interaction of the middle region with histones has not been further taken into account in the manuscript, e.g. the authors perform the ITC assay only with Chz-C as well as the crystal structure. While not everything can be experimentally tested the fact that OsChz can make additional contacts with histones via the middle region should at least have been more extensively discussed.

The crystal of OsChz in complex with H2A-H2B revealed several amino acids interacting with H2A/H2A.Z-H2B. The residues are conserved between H2A and H2A.Z. How do the authors then interpret the differential affinity for H2A versus H2A.Z containing dimers observed in Figure 2D?

A closer look at the alignment in SFig 6 shows that the residues (red asterix) involved in the interaction with H2A-H2B are not necessarily conserved in other plant species. How do the authors interpret this observation? Do they imply different modes of interaction?

In Figure 6 & 7 nucleosome occupancy is considered on the one hand and H2A.Z occupancy on the other but the two are not compared. Do the same regions that show a reduction or an increase in nucleosome density also show differences in H2A.Z enrichment? Does the global loss of nucleosome occupancy explain the loss of H2A.Z?

Are there also regions that show an increase in H2A.Z occupancy?

In the introduction (line 69), the authors state that H2A.Z enrichment at the TSS associates with transcriptional activation. However, in their own observations (Figure 7, line 374) they find a general negative correlation between H2A.Z enrichment and expression, which seems in contradiction. Their data (Figure 7d) show however at least some indication that genes with high gene body H2A.Z are expressed to lower levels as those with low H2A.Z at gene bodies. Indeed the role of H2A.Z in gene regulation is complex and is partly also regulated through different post-translational modifications (including acetylation and ubiquitinylation, e.g. Gómez-Zambrano et al). This should be discussed.

Concerning the enrichment of OsChz1 along the genome (Figure 8): Are the mis-regulated genes specifically enriched among the genes with OsChz1 peaks? Similarly, are the genes showing OsChz1 peaks particularly enriched among those with differential H2A.Z enrichment or nucleosomal density changes?

The discussion should be revised; nearly half a page is dedicated to NAP1 histone chaperone. This could be shortened. It may be more interesting to discuss the fact that Chz binds both to H2A-H2B and H2A.Z-H2B and whether it could interact with the other H2A variants; the impact on OsChz loss

on nucleosomal occupancy, the way that the middle region of Chz interacts with histones. It may also be useful to discuss Chz function in other plants, e.g. the model organism Arabidopsis that expresses two Chz homologues. Are the two likely to function in the same way?

Minor comments:

Material and Methods should be carefully revised. It is partly written as protocol style e.g. line 654 "Grind leaves in liquid nitrogen to fine powder".

How have the proteins shown in Figure 1B been selected from the species in A (e.g. only one of the two Ath proteins is shown). It would be useful to make a more careful phylogenetic analysis. The authors state that most organisms have two Chz copies. Do they result from recent duplications or is a copy lost in the rice genome?

The description of the crystal structure is difficult to follow for a non-expert and could be improved.

Are the H2A/H2A.Z residues that interact with OsChz conserved in H2A.X and H2A.W?

I am not convinced that Figure 6b is very useful. One can simply state in the text that compared to the number of genes and TEs in the genome, genes are significantly differentially expressed compared to TEs. Instead it might be useful to move some of the supplementary data into the main figure (e.g. Figure 12 a,b and an example of e).

short-length genes correct to shorter genes
big-length genes correct to longer genes

Reviewer #2 (Remarks to the Author):

In this work the authors perform functional and structural analyses of the rice CHZ domain-containing protein OsChz1. The structure of OsChz1 residue 432-446 in the complex of H2A-H2B dimer unravels a histone binding mechanism that is distinct from those revealed in other known structures. Structural data are further supported by nice biochemical analysis. OsChz1 depletion results in genome-wide alternation of nucleosome occupancy and reduction of H2A.Z, leading to multiple developmental defects. Overall, the work is interesting and, although not consistent with previous observations for yeast Chz1, shows mechanistic details underlying plant Chz1 protein. This study is of high interest and importance for histone chaperone researchers. There are only few comments that need to be addressed before the publication of the manuscript.

1)The authors should label the interactions described in lines 198-209 in Fig 3b in order to increase the readability. The dashed line drawn between E446 and L67 is misleading as the side chains of these two residues could not form hydrogen bonds (Fig 3c).

2) The crystal structure and refinement statistics of OsChz1 432-446aa/H2A-H2B dimer complex are technically sound. However, it will be helpful if the authors could discuss the interaction between EEVNVE(440-446aa) and H2A-H2B dimer. These acidic residues locate atop of the so-called nucleosome acidic patch, which is typically anchored by positively-charged residues.

3) The authors show that OsChz1 facilitate nucleosome assembly. The curve of OsChz1-mediated histone dimer incorporation presented in Fig S8C and Fig 2F seems different. The authors may need to explain the discrepancy between these results, in which the concentration of OsChz1-bound H2A-H2B dimer required for achieving 100% nucleosome assembly varies.

4) The results of ITC and nucleosome assembly assay suggest that OsChz1 presents a modest preference for H2A-H2B over H2A.Z-H2B. Moreover, the GST pull-down assay reveals that OsChz1-C (338-471aa) shows H2A-H2B preference. Although it is not necessary, the authors could speculate, based on the complex structure, whether the OsChz1 390-422aa confers the H2A-H2B preference to OsChz1.

Reviewer #3 (Remarks to the Author):

The Authors present the functional and structural characterization of the rice potential homolog of the Chz1 in yeast. Chz1 is a major chaperone for H2A.Z in yeast involved in nuclear import of H2A.Z . Chz1 forms a trimeric complex with Z-B dimer and cooperates with SWR1 in histone exchange. Compared with Arabidopsis, the rice genome contains a single gene encoding Chz1, which facilitates its functional characterization using genetics.
The Authors report that in contrast with Chz1 from yeast, OsChz1 binds both to H2A.Z-H2B and H2A-H2B with higher affinity for the latter. In vitro assays also support that OsChz1 assists nucleosome assembly equally well with H2A or H2A.Z as substrate. The Authors obtain a crystal structure and identify residues involved in binding H2A but these residues differ from those reported in crystal structure from yeast Chz. The Authors obtain two mutant alleles of OsChz1 and report various developmental defects in line with degrees of transcription defects. They show that loss of Chz1 causes a general loss of H2A.Z and use a tagged Chz1 that genetically complements the loss of function mutant to localize Chz1 on chromatin. They report a global co-localization of Chz1 with region occupied by H2A.Z, which are enriched in H3K27me3 and with lower levels of DNA methylation. However, they show no change in DNA methylation levels in Chz1 KO mutants.

Basically, this is an interesting report. The first half of the report confirms to some extent previous finding reported in 2019 (https://doi.org/10.1371/journal.pbio.3000277). The functional analysis of OsChz1in rice of interest but to some degree at odds with the biochemical characterization that confirms the lack of specific binding of OsChz1 to H2A.Z/H2A. The Authors argue a preferential role of OsChz1 in the deposition of H2A.Z, when they declare the opposite concerning its affinity to H2A/H2A.Z.

Additional major concerns that should be addressed are listed below.

Major points

1) Histone deposition assay (and related to histone binding)
Based on in vitro assays the Authors argue the preferential activity of histone deposition and histone binding for H2A in OsChz1. However, the affinity constant reported differ from that reported in Wang et al., 2019 for Arabidopsis Chz1 and this is not really taken into account. There appears to be less incorporation of H2A.Z-H2B dimers into tetrasome to form nucleosome compared to H2A-H2B even in the absence of OsChz1 (supplementary figure 5b), suggesting that H2A.Z-H2B dimer used in this report might contain homotypic dimers of H2B or H2A.Z. The ratio of Chz1-H2A.Z is very odd compared to H2A.Z in figure 2d, further suggesting misfolding of H2A.Z. It is unclear how heterodimers were prepared and the Authors should provide evidence that they used gel filtration after reconstitution of histone dimers, which is very important to remove such misfolded histone dimers.

2) crystal structure of OsChz1-C and H2A-H2B dimer
it is not clear how the Authors use the crystal structure to see the difference between OsChz1 and yeast Chz1. They report that Y59 and L67 in H2A (these residues are common with H2A.Z) interact

with N444 and E446 of OsChz1, but it is not clear if this binding mode is conserved in yeast. The Authors should superimpose crystal structures obtained from OsChz1 and yeast Chz1 to see the overall structure. Then they will be able to conclude if the binding mode of OsChz1 to histone is different from yeast Chz1. It would be helpful to focus on the H2A residues (P and N in H2A whereas A and G in H2A.Z) that were defined as important residues for specific bind to Chz1 in the previous report (Wang et al., 2019)

3) Impact of KO of OsChz1 on gene expression.
The analysis is rather limited to Venn diagram, and would require in depth clustering to clarify if there are distinct group of genes affected by the loss of OsChz1 and if there is a link with nucleosome occupancy. The difference shown in Fig 6d is really not convincing and the MNase data required analysis in further depth using clustering together with the expression data.

4) Impact on Chromatin composition in H2A/H2A.Z.
It is quite unclear why, whereas the Authors emphasize the fact the absence of specific binding of OsChz1 to H2A.Z, they profile only H2A.Z. Profiles of H2A should be provided in WT and the KO mutant. In addition, normalization should not use "input" but H3, because nucleosome occupation changes in the mutant versus WT and here the Authors might rather report the effect of nucleosome occupation. The specificity of the anti-H2A.Z used is unclear and should be demonstrated.

5) OsChz1 localization.
In Yeast, Chz1 delivers H2A.Z-H2B heterodimers to the SWR1 complex, in charge of the deposition of H2A.Z. Hence, Chz1 is not expected to contact chromatin in a specific manner. ChIP is performed after crosslinking, which is liable to generate artifacts – Here the localization of OsChz using ChIpseq lacks a proper control, which is the localization of OsChz1-MYC in the KO mutant background versus the KO mutant without the construct to eliminate background signal. Also, the Authors should compare the expression levels of the OsChZ1MYC fusion protein with WT at least using gene expression levels as a proxy if they are unable to do this using Mass-spec. Without this control it is not possible to interpret this dataset.

6) In the end it is unclear whether OsChz1 is homolog to Chz1 or not. The Authors should discuss this point in detail.

Minor points
1) histone binding activity of OsChz1-M
The Authors report that OsChz1-M (containing PTZ001908 motif whereas yeast does not have this domain) and OsChz1-C (containing the conserved CHZ domain) bind to histone with a preference with H2A in Chz1-C (supplementary figure 4). The fact that the CHZ domain can bind to histone is not emphasized and should be described in further detail.

AUTHOR RESPONSES to REVIEWER COMMENTS

Reviewer #1 (Remarks to the Author):

This is an interesting and comprehensive study characterizing the rice homologue of yeast Chz involved in H2A.Z deposition. This study combines structural data on OsChz binding to H2A-H2B dimers with biochemical studies, analysis of a rice mutant for the unique Chz gene in the rice genome and genome wide studies of nucleosomal occupancy, H2A.Z and OsChz enrichment and gene expression. This study is therefore original and rather complete and brings novel insight into the function of a CHZ-domain protein in plants.
Response: We thank Reviewer #1 for positive consideration of this work.

Several points need to be further considered:

In Figure 2 the authors have divided OsChz in 3 non-overlapping protein regions to study interaction with H2A-H2B and H2A.Z-H2B (N 1-78, M 79-337 and C 338-471, the latter comprising the Chz domain). SFig4 shows that both M and C regions of OsChz are able to bind histones with Chz-C preferentially binding H2A over H2A.Z, while this is not the case for the middle region. This suggests that Chz may interact with histones in different ways, implicating different regions of the Chz protein. The interaction of the middle region with histones has not been further taken into account in the manuscript, e.g. the authors perform the ITC assay only with Chz-C as well as the crystal structure. While not everything can be experimentally tested the fact that OsChz can make additional contacts with histones via the middle region should at least have been more extensively discussed.
Response: The M-region fragment OsChz1-M is less stable at room temperature and unsuitable in ITC analysis. In our pull-down assays, differing from OsChz1-M that displayed almost similar binding to H2A-H2B and H2A.Z-H2B, OsChz1-C showed higher binding to H2A-H2B than to H2A.Z-H2B. We used ITC to quantitatively compare the binding of OsChz1-C to H2A-H2B and H2A.Z-H2B. We have now clarified this by the statement ‘Visually, OsChz1-C seemed to pulldown more efficiently H2A-H2B than H2A.Z-H2B. To verify this, we examined the binding of OsChz1-C to H2A-H2B and H2A.Z-H2B by isothermal titration calorimetry (ITC) experiments’ in the Results section, and added ‘the OsChz1-M fragment also displayed binding activity to H2A-H2B and H2A.Z-H2B, raising

the question whether one OsChz1 molecule binds two molecules of H2A-H2B/H2A.Z-H2B' in the Discussion section of our revised manuscript.

The crystal of OsChz in complex with H2A-H2B revealed several amino acids interacting with H2A/H2A.Z-H2B. The residues are conserved between H2A and H2A.Z. How do the authors then interpret the differential affinity for H2A versus H2A.Z containing dimers observed in Figure 2D?
Response: The OsChz1-C sequences are rich in acidic amino acids and some regions are highly flexible to be captured in crystal structure. Our crystal structure of OsChz1 in complex with H2A-H2B provided insight into the molecular basis of binding but could not explain the preferential binding of OsChz1-C with H2A-H2B over H2A.Z-H2B. We have now added a statement as 'the N-terminus-shortened OsChz1-C showed higher binding-affinity to H2A-H2B over H2A.Z-H2B, but both the molecular basis and the functional significance of this preference remains unclear' in the Discussion section of our revised manuscript.

A closer look at the alignment in SFig 6 shows that the residues (red asterix) involved in the interaction with H2A-H2B are not necessarily conserved in other plant species. How do the authors interpret this observation? Do they imply different modes of interaction?
Response: As histones are highly basic proteins, numerous histone chaperones have long acidic stretches but these amino acids are not necessarily conserved in sequence alignments, as an example one can refer to the alignment of the NAP1 family proteins (Dong *et al*., 2003, Planta 216: 561-570). Chz1 follows this general knowledge, and we believe that we cannot speculate different modes of interaction by simple residue conservation in sequence alignment.

In Figure 6 & 7 nucleosome occupancy is considered on the one hand and H2A.Z occupancy on the other but the two are not compared. Do the same regions that show a reduction or an increase in nucleosome density also show differences in H2A.Z enrichment? Does the global loss of nucleosome occupancy explain the loss of H2A.Z?
Response: We have now added the comparison between H2A.Z enrichment and nucleosome occupancy changes in *oschz1* as Fig 7f in our revised manuscript. It was found that there is an overlap at a subset of genes but a higher number of genes showed separately either a loss of H2A.Z or a nucleosome down-occupancy, indicating that the global loss of nucleosome occupancy cannot fully explain the loss of H2A.Z.

Are there also regions that show an increase in H2A.Z occupancy?

Response: During our manuscript revision, Wang *et al*. (Nat. Commun. 2020 Jun 8;11(1):2887) published an interesting work showing that the Arabidopsis NAP1-RELATED PROTEIN1 (NRP1) and NRP2 negatively regulate H2A.Z abundance in chromatin and their loss causes over-accumulation of H2A.Z genome-wide. Differing from NRP1/2, loss of OsChz1 resulted in loss of H2A.Z genome-wide. We have now cited and discussed the NRP1/2 work in our revised manuscript (Ref 55).

In the introduction (line 69), the authors state that H2A.Z enrichment at the TSS associates with transcriptional activation. However, in their own observations (Figure 7, line 374) they find a general negative correlation between H2A.Z enrichment and expression, which seems in contradiction. Their data (Figure 7d) show however at least some indication that genes with high gene body H2A.Z are expressed to lower levels as those with low H2A.Z at gene bodies. Indeed the role of H2A.Z in gene regulation is complex and is partly also regulated through different post-translational modifications (including acetylation and ubiquitinylation, e.g. Gómez-Zambrano et al). This should be discussed.

Response: We apologize for the missed reference. We have now corrected the sentence in our introduction as '…indicate that the presence of H2A.Z at TSS associates with transcriptional activation or repression' and cited the work of Gómez-Zambrano *et al*. in our revised manuscript (Ref 24).

Concerning the enrichment of OsChz1 along the genome (Figure 8): Are the mis-regulated genes specifically enriched among the genes with OsChz1 peaks? Similarly, are the genes showing OsChz1 peaks particularly enriched among those with differential H2A.Z enrichment or nucleosomal density changes?

Response: We have improved the OsChz1 enrichment analysis by using *oschz1-1* to eliminate background signal. Cross-comparisons revealed that both up-regulated and down-regulated genes exhibit lower levels of H2A.Z in *oschz1-1* (Supplementary Fig 14). A significant overlap was observed for OsChz1-binding with genes showing loss of H2A.Z but not with those genes exhibiting only nucleosome down-occupancy (new Fig 8d added in our revised manuscript). We believe that some nucleosome occupancy changes may not require a steady binding of OsChz1 on chromatin.

The discussion should be revised; nearly half a page is dedicated to NAP1 histone chaperone. This could be shortened. It may be more interesting to discuss the fact that Chz binds both to H2A-H2B and H2A.Z-H2B and whether it could interact with the other H2A variants; the impact on OsChz loss on nucleosomal occupancy, the way that the middle region of Chz interacts with histones. It may also be useful to discuss Chz function in other plants, e.g. the model organism Arabidopsis that expresses two Chz homologues. Are the two likely to function in the same way?

Response: We thank the reviewer for this nice suggestion. We have followed the reviewer's suggestion and revised our discussion accordingly, excepting the point regarding Chz1 homologues in Arabidopsis. To our knowledge, the function of the Arabidopsis Chz1 two homologues had not been characterized so far. We have some unpublished data but it is too early to discuss in this current manuscript.

Minor comments:

Material and Methods should be carefully revised. It is partly written as protocol style e.g. line 654 "Grind leaves in liquid nitrogen to fine powder".

Response: We have reorganized and carefully revised Materials and Methods. We believe that their quality has been significantly improved.

How have the proteins shown in Figure 1B been selected from the species in A (e.g. only one of the two Ath proteins is shown). It would be useful to make a more careful phylogenetic analysis. The authors state that most organisms have two Chz copies. Do they result from recent duplications or is a copy lost in the rice genome?

Response: We had chosen for a given species one homologue appeared on top of the phylogenetic tree. We have now combined Fig 1b together with Fig 1a and showed protein domain organization for all members correspondingly with the tree (now Fig 1a in our revised manuscript). For most organisms that have two Chz1 copies, the two homologues are closely associated on a branch of the phylogenetic tree, suggesting that they might be derived from a recent gene duplication during evolution. Nevertheless, because homologies are limited essentially to the CHZ-domain, more detailed analyses of the genomes are necessary to inspect into duplication or deletion events, but this is out of the scope of our current manuscript.

The description of the crystal structure is difficult to follow for a non-expert and could be improved.

Response: We have revised the text to make it easier to follow.

Are the H2A/H2A.Z residues that interact with OsChz conserved in H2A.X and H2A.W?

Response: We have performed the alignment and included it in Supplementary Fig 1. The interacting residues are conserved in H2A.X and H2A.W. We have added a statement as ‘These residues are also conserved in the variant H2A.W and H2A.X (Supplementary Fig 1b), which marks constitutive heterochromatin and responsive DNA-damage sites in euchromatin, respectively (Ref 52). Whether or not can OsChz1 bind to H2A.W and H2A.X remains unknown’ in the Discussion section of our revised manuscript.

I am not convinced that Figure 6b is very useful. One can simply state in the text that compared to the number of genes and TEs in the genome, genes are significantly differentially expressed compared to TEs. Instead it might be useful to move some of the supplementary data into the main figure (e.g. Figure 12 a,b and an example of e).

Response: Fig 6b shows that loss of OsChz1 significantly affected expression of PCGs (mostly at euchromatin) but not TEs (mostly at heterochromatin). We have followed the reviewer’s suggestion and moved Supplementary Fig 12a and 12b in the previous version into the main Fig 6 in our revised manuscript.

short-length genes correct to shorter genes

big-length genes correct to longer genes

Response: done

Reviewer #2 (Remarks to the Author):

In this work the authors perform functional and structural analyses of the rice CHZ domain-containing protein OsChz1. The structure of OsChz1 residue 432-446 in the complex of H2A-H2B dimer unravels a histone binding mechanism that is distinct from those revealed in other known structures. Structural data are further supported by nice biochemical analysis. OsChz1 depletion results in genome-wide alternation of nucleosome occupancy and reduction of

H2A.Z, leading to multiple developmental defects. Overall, the work is interesting and, although not consistent with previous observations for yeast Chz1, shows mechanistic details underlying plant Chz1 protein. This study is of high interest and importance for histone chaperone researchers. There are only few comments that need to be addressed before the publication of the manuscript.

Response: We thank Reviewer #2 for positive consideration of this work. We have addressed his/her comments as outlined below.

1)The authors should label the interactions described in lines 198-209 in Fig 3b in order to increase the readability. The dashed line drawn between E446 and L67 is misleading as the side chains of these two residues could not form hydrogen bonds (Fig 3c).

Response: We have followed the reviewer's suggestion for labeling in Fig 3b and corrected our mistake by removing the misleading dashed line in Fig 3c.

2) The crystal structure and refinement statistics of OsChz1 432-446aa/H2A-H2B dimer complex are technically sound. However, it will be helpful if the authors could discuss the interaction between EEVNVE(440-446aa) and H2A-H2B dimer. These acidic residues locate atop of the so-called nucleosome acidic patch, which is typically anchored by positively-charged residues.

Response: Within the crystal structure, OsChz1 D440 showed interactions with H2B and OsChz1 N444 and E446 showed interactions with H2A. The location of EEVNVE atop of the nucleosome acidic patch may counteract a strong binding of OsChz1 to the nucleosome, which would be in agreement with our observation of the absence of steady OsChz1 binding with nucleosome-occupancy changes. However, we feel this assumption is too far to be discussed in our current manuscript, future functional analyses of mutated OsChz1 sequences in rescue of *oschz1* together with their binding assessment at chromatin will provide substantial information to discuss this issue.

3) The authors show that OsChz1 facilitate nucleosome assembly. The curve of OsChz1-mediated histone dimer incorporation presented in Fig S8C and Fig 2F seems different. The authors may need to explain the discrepancy between these results, in which the concentration of OsChz1-bound H2A-H2B dimer required for achieving 100% nucleosome assembly varies.

Response: We apologize for the confusion. The concentration of OsChz1 used in Fig 2f was 0.1 μM whereas that in Fig S8C was 0.2 μM. It is also worth to note that the maximum value

of nucleosome band rather than the value of achieving complete nucleosome assembly was set to 100%. We have now clarified these issues in the legends of Fig 2f and Supplementary Fig 7 (Fig S8 in previous version) in our revised manuscript.

4) The results of ITC and nucleosome assembly assay suggest that OsChz1 presents a modest preference for H2A-H2B over H2A.Z-H2B. Moreover, the GST pull-down assay reveals that OsChz1-C (338-471aa) shows H2A-H2B preference. Although it is not necessary, the authors could speculate, based on the complex structure, whether the OsChz1 390-422aa confers the H2A-H2B preference to OsChz1.

Response: Our crystal structure of OsChz1 in complex with H2A-H2B provided insight into molecular basis of binding but could not explain the preferential binding of OsChz1-C with H2A-H2B over H2A.Z-H2B. The OsChz1-C sequences are rich in acidic amino acids and some regions are highly flexible to be captured in crystal structure. We have now added a statement as 'the N-terminus-shortened OsChz1-C showed higher binding-affinity to H2A-H2B over H2A.Z-H2B, but both the molecular basis and the functional significance of this preference remains unclear' in the discussion of our revised manuscript.

Reviewer #3 (Remarks to the Author):

The Authors present the functional and structural characterization of the rice potential homolog of the Chz1 in yeast. Chz1 is a major chaperone for H2A.Z in yeast involved in nuclear import of H2A.Z . Chz1 forms a trimeric complex with Z-B dimer and cooperates with SWR1 in histone exchange. Compared with Arabidopsis, the rice genome contains a single gene encoding Chz1, which facilitates its functional characterization using genetics. The Authors report that in contrast with Chz1 from yeast, OsChz1 binds both to H2A.Z-H2B and H2A-H2B with higher affinity for the latter. In vitro assays also support that OsChz1 assists nucleosome assembly equally well with H2A or H2A.Z as substrate. The Authors obtain a crystal structure and identify residues involved in binding H2A but these residues differ from those reported in crystal structure from yeast Chz. The Authors obtain two mutant alleles of OsChz1 and report various developmental defects in line with degrees of transcription defects. They show that loss of Chz1 causes a general loss of H2A.Z and use a tagged Chz1 that genetically complements the loss of function mutant to localize Chz1 on chromatin. They report a global co-localization of Chz1 with region occupied by H2A.Z, which are enriched in H3K27me3 and with lower levels of DNA methylation. However, they

show no change in DNA methylation levels in Chz1 KO mutants.

Basically, this is an interesting report. The first half of the report confirms to some extent previous finding reported in 2019 (https://doi.org/10.1371/journal.pbio.3000277). The functional analysis of OsChz1in rice of interest but to some degree at odds with the biochemical characterization that confirms the lack of specific binding of OsChz1 to H2A.Z/H2A. The Authors argue a preferential role of OsChz1 in the deposition of H2A.Z, when they declare the opposite concerning its affinity to H2A/H2A.Z.

Response: We thank Reviewer #3 for appreciations and interests of this work. However, we cannot agree with his/her latter comments relating our report to the previous publication by Wang *et al*. in 2019 (https://doi.org/10.1371/journal.pbio.3000277; Ref 32, now as Ref 33 in our revised manuscript). Wang *et al*. (2019) made an excellent work providing important insight into the molecular basis of preferential binding of the yeast yChz1 with H2A.Z. In the Supplementary Fig 4 of their publication, they showed that the Arabidopsis AtChz1b (residues 253–463) binds both H2A-H2B and H2A-H2A.Z in ITC experiments. We believe that our report on the rice OsChz1 is much more beyond. Our biochemical characterization includes multiple independent *in vitro* and *in vivo* experiments, we tested not only the C-terminal but also other regions as well as full-length of OsChz1 in different assays, and our crystal structure provided key insight into the molecular basis of OsChz1 interaction with H2A-H2B. To our knowledge, neither Arabidopsis AtChz1 nor Chz1 from any other plant species had been studied for their biological functions previously. Our loss-of-function of *OsChz1* demonstrated essential roles of this gene in flowering time control as well as in several other processes. These findings are important, at least for the plant research field. Lastly, our *in vitro* assays showed that OsChz1-C displayed a higher affinity to H2A-H2B over H2A.Z-H2B, but OsChz1-M or full-length OsChz1 displayed almost similar affinity to H2A-H2B and H2A.Z-H2B. Our *in vivo* analysis revealed that loss of OsChz1 caused nucleosome occupancy changes as well as reductions of H2A.Z enrichment genome-wide. We consider these *in vitro* and *in vivo* data consistent but not opposite. For clarity, we have revised our text by constantly specifying that the higher affinity to H2A-H2B over H2A.Z-H2B was observed with OsChz1-C, and by adding a statement as 'the N-terminus-shortened OsChz1-C showed higher binding-affinity to H2A-H2B over H2A.Z-H2B, but both the molecular basis and the functional significance of this preference remains unclear' in the discussion of our revised manuscript.

Additional major concerns that should be addressed are listed below.

Major points

1) Histone deposition assay (and related to histone binding)

Based on in vitro assays the Authors argue the preferential activity of histone deposition and histone binding for H2A in OsChz1. However, the affinity constant reported differ from that reported in Wang et al., 2019 for Arabidopsis Chz1 and this is not really taken into account. There appears to be less incorporation of H2A.Z-H2B dimers into tetrasome to form nucleosome compared to H2A-H2B even in the absence of OsChz1 (supplementary figure 5b), suggesting that H2A.Z-H2B dimer used in this report might contain homotypic dimers of H2B or H2A.Z. The ratio of Chz1-H2A.Z is very odd compared to H2A.Z in figure 2d, further suggesting misfolding of H2A.Z. It is unclear how heterodimers were prepared and the Authors should provide evidence that they used gel filtration after reconstitution of histone dimers, which is very important to remove such misfolded histone dimers.

Response: Wang *et al*. (2019) showed that the affinity constant obtained in ITC depends on salt concentration in the binding assay, e.g. for AtChz1b (residues 253–463) binding with H2A-H2B: Kd = 30 nM at 0.1M NaCl but Kd = 10600 nM at 0.25M Nacl, and for AtChz1b (residues 253–463) binding with H2A.Z-H2B: Kd = 50 nM at 0.1M NaCl but Kd = 19900 nM at 0.25M Nacl (Fig S4B in Ref 33). Our ITC was performed at 0.1M NaCl, and OsChz1-C showed binding to H2A-H2B and H2A-H2A.Z with Kd = 9.8 nM and 64.1 nM, respectively (Fig 2d; now Fig 2e in our revised manuscript). The Kd values for H2A.Z-H2B binding of the Arabidopsis AtChz1b (residues 253–463) and the rice OsChz1-C are closely similar (50 nM compared to 64.1 nM). We don't see any 'odd' and/or any reason to speculate 'misfolding' from our analyses. Furthermore, the chromatography of H2A.Z-H2B elution shows symmetric and shape peak indicating for high pure heterodimer quality, and SDS-PAGE gel analysis confirms this conclusion (graph and gel showing below for information).

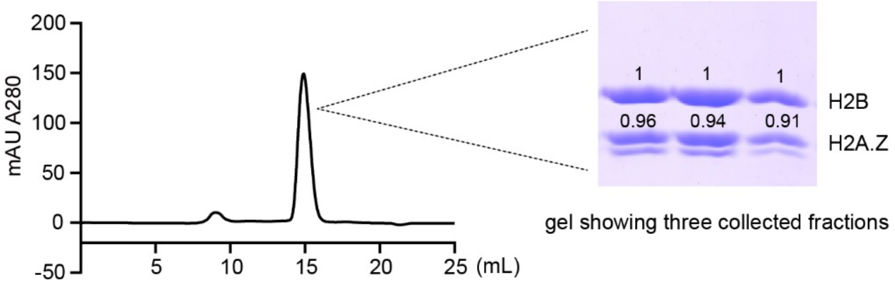

2) crystal structure of OsChz1-C and H2A-H2B dimer

it is not clear how the Authors use the crystal structure to see the difference between OsChz1 and yeast Chz1. They report that Y59 and L67 in H2A (these residues are common with H2A.Z) interact with N444 and E446 of OsChz1, but it is not clear if this binding mode is conserved in yeast. The Authors should superimpose crystal structures obtained from OsChz1 and yeast Chz1 to see the overall structure. Then they will be able to conclude if the binding mode of OsChz1 to histone is different from yeast Chz1. It would be helpful to focus on the H2A residues (P and N in H2A whereas A and G in H2A.Z) that were defined as important residues for specific bind to Chz1 in the previous report (Wang et al., 2019)

Response: The residues A57 and G98 in H2A.Z are buried by yChz1 in the yChz1-H2A.Z-H2B complex, but in the OsChz1-H2A-H2B complex we did not observe any direct interaction between the corresponding residues P50 and N91 in H2A with OsChz1. The differences of the two crystal structures were evident that does not require a superimposition, and the superimposition failed to provide more information. The α-helical structures of yChz1 involved in interactions with H2A.Z A57 and G98, as described in Wang *et al*. (2019), are not conserved in OsChz1.

3) Impact of KO of OsChz1 on gene expression.

The analysis is rather limited to Venn diagram, and would require in depth clustering to clarify if there are distinct group of genes affected by the loss of OsChz1 and if there is a link with nucleosome occupancy. The difference shown in Fig 6d is really not convincing and the MNase data required analysis in further depth using clustering together with the expression data.

Response: The nucleosome occupancy difference between *oschz1* and WT, shown in Fig 6d, is statistically significant. Clustering analysis revealed that the nucleosome occupancy reduction is associated with up-regulated but not down-regulated genes in *oschz1-1*. Additionally, these conclusions were similarly obtained in analysis of nucleosome occupancy across the gene body (Fig 6f and 6g) or just adjacent to TSS (Supplementary Fig 11 added in our revised manuscript). We have carefully revised the corresponding text in our revised manuscript for clarity.

4) Impact on Chromatin composition in H2A/H2A.Z.

It is quite unclear why, whereas the Authors emphasize the fact the absence of specific binding of OsChz1 to H2A.Z, they profile only H2A.Z. Profiles of H2A should be provided in WT and the KO mutant. In addition, normalization should not use "input" but H3, because nucleosome occupation changes in the mutant versus WT and here the Authors might rather report the effect of nucleosome occupation. The specificity of the anti-H2A.Z used is unclear and should be demonstrated.

Response: We have tried two different commercially available H2A antibodies (Abcam, ab18255; Abmart, T55481S) but failed to obtain satisfactory results in rice ChIP-seq. We noticed that most of the previous published ChIP-seq studies in rice or Arabidopsis did not include H2A. One exception came up during our manuscript revision: Wang *et al*. (Nat Commun. 2020 Jun 8;11(1):2887; Ref 55 in our revised manuscript) reported unchanged genome-wide H2A enrichment in *nrp1nrp2* by using home-made antibodies generated against Arabidopsis H2A peptide. Similar approach in rice would need additional time. Most importantly, in our study MNase-seq already provided important information about OsChz1 function in genome-wide nucleosome occupancy. We consider that H2A ChIP-seq could provide additional interesting information but its lack does not prevent appreciations of OsChz1 functions as already described in our manuscript.

We have followed the reviewer's suggestion and performed H3 ChIP-seq and normalized H2A.Z to H3. We found that the H3 levels were largely similar but the normalized H2A.Z levels remained significantly reduced across the gene body in *oschz1-1* as compared to WT, which were shown as Fig 7g and described in our revised manuscript. The anti-H2A.Z antibody had been generated against rice H2A.Z peptides and its specificity had been already validated in the previous study, as cited Ref 59.

5) OsChz1 localization.

In Yeast, Chz1 delivers H2A.Z-H2B heterodimers to the SWR1 complex, in charge of the deposition of H2A.Z. Hence, Chz1 is not expected to contact chromatin in a specific manner. ChIP is performed after crosslinking, which is liable to generate artifacts – Here the localization of OsChz using ChIpseq lacks a proper control, which is the localization of OsChz1-MYC in the KO mutant background versus the KO mutant without the construct to eliminate background signal. Also, the Authors should compare the expression levels of the OsChZ1MYC fusion protein with WT at least using gene expression levels as a proxy if they are unable to do this using Mass-spec. Without this control it is not possible to interpret this dataset.

Response: We have followed the reviewer's suggestion and performed ChIP-seq by using complemented line COM1 for OsChz1-MYC and using *oschz1-1* to eliminate background signal. The expression data were now shown in Supplementary Fig 9c and 9d, and the ChIP-seq results shown in Fig 8a in our revised manuscript. While the major conclusions remained unchanged, these new data significantly improved our manuscript quality. We thank the reviewer for this very helpful suggestion.

6) In the end it is unclear whether OsChz1 is homolog to Chz1 or not. The Authors should discuss this point in detail.
Response: OsChz1 contain a CHZ-domain and our study clearly shows that OsChz1 binds both H2A-H2B and H2A.Z-H2B and is required for proper nucleosome occupancy across the genome and for H2A.Z incorporation in chromatin. Binding of AtChz1 to both H2A-H2B and H2A.Z-H2B in ITC was also reported in Wang *et al*. (2019). We don't feel necessity to discuss back the nomenclature of CHZ-domain proteins.

Minor points
1) histone binding activity of OsChz1-M
The Authors report that OsChz1-M (containing PTZ001908 motif whereas yeast does not have this domain) and OsChz1-C (containing the conserved CHZ domain) bind to histone with a preference with H2A in Chz1-C (supplementary figure 4). The fact that the CHZ domain can bind to histone is not emphasized and should be described in further detail.
Response: In contrast, our GST pull-down assay showed that CHZ-domain alone did not bind H2A-H2B nor H2A.Z-H2B (Supplementary figure 4; now Fig 2d in our revised manuscript). This is expected because the OsChz1 residues involved in interaction with H2A are located outside of the CHZ-domain (Supplementary Fig 5 in our revised manuscript). Similarly, the yeast yChz1 also interacted with H2A.Z through residues located outside of the CHZ-domain (Wang *et al*., 2019; indicated also in our Supplementary Fig 5).

Reviewer #1 (Remarks to the Author):

In the revised version of the manuscript, the authors have responded well to most of my criticisms and the revised version is improved, including Discussion and Materials and Methods section.

In my opinion, the authors also addressed Reviewer 3's comments in a satisfactory manner.
- They removed the doubts on the mis-folding of H2A.Z dimers (Figure provided for the Reviewer)
- The reviewer requested a ChIP-seq with an H2A antibody. As the authors had technical problems with available H2A antibodies, this experiment was not included in this study. As suggested by the reviewer they performed H3-ChIP-seq for normalization of H2A.Z.
- Finally the authors included new datasets for the OsChz1 ChIP-seq analysis including a complemented line OsChz1-MYC and the knock-out oschz1-1 mutant to eliminate background signals.

While most of the new data are convincing, I just have a concern with the discrepancy between the new H3-ChIP data in WT and the oschz1-1 mutant (Figure 7) and the MNase data (Figure 6) as they should in principle reveal the same phenomenon: reduced nucleosomal occupancy in the mutant.
While there is a little less H3-occupancy in the 3' region of genes in the oschz1-1 mutant, no major difference can be found at the TSS in Figure 7G compared to Figure 6d.
How do the authors explain this? Do they envisage remaining tetrasomes revealed by the H3-ChIP that would allow digestion of DNA by MNase?

Minor comments:

The authors have not really responded to the question whether in their dataset (Figure 7) there are also regions that show an increase in H2A.Z occupancy?

line 188: The absence of OsChz1 preference for H2A.Z or even a significant preference of OsChz1-C for H2A is in contrast to
should it not read ... a significant preference of OsChz1-C for H2A.Z is in ...

line 392 and 393: trends = tends

nucleosome down occupancy (several times in the text) should be replaced by reduced nucleosome occupancy

line 470: H2A.X marks " responsive DNA-damage sites in euchromatin". Maybe better: that can be phosphorylated upon DNA-damage.
H2A.X marks DNA-damage sites in its phosphorylated form.

Reviewer #2 (Remarks to the Author):

The revised manuscript has addressed most of my concerns on the structural parts.

It is worth noting that the CHZ domain-containing proteins may present less or no preference for H2A.Z-H2B dimer, as demonstrated by OsChz1 in this study and AtChz1b in the previous study (Wang et al.). The high eukaryotes CHZ domain-containing proteins have been considered functionally related to yeast Chz1 until Wang et al. reveal the molecular basis for the preferential recognition of H2A.Z-H2B dimer by Chz1. In fact, most CHZ domain-containing proteins lack the key residues that are selectively interacting with H2A.Z. From my point of view, the CHZ domain-containing proteins do not necessarily show specificity to H2A.Z. It should be reasonable if these proteins have distinct histone

dimer binding preferences.

The authors may need to consider to tune down the conclusion regarding the OsChz1 binding preference for H2A over H2A.Z. First, OsChz1-C presents a limited preference for H2A over H2A.Z, as suggested by the pull-down and the ITC experiments. Second, although the crystal structure revealed some interactions between OsChz1 and H2A-H2B dimer, it lacks H2A specific residues involving the preferential binding of OsChz1-C.

Minor points
The residues numbers of aligned sequences, as least for OsChz1, should be given in the FigS3 and FigS5.

[Editor: Reviewer #3: unavailable.]

AUTHOR RESPONSES to REVIEWER COMMENTS

Reviewer #1 (Remarks to the Author):

In the revised version of the manuscript, the authors have responded well to most of my criticisms and the revised version is improved, including Discussion and Materials and Methods section.

In my opinion, the authors also addressed Reviewer 3's comments in a satisfactory manner.
- They removed the doubts on the mis-folding of H2A.Z dimers (Figure provided for the Reviewer)
- The reviewer requested a ChIP-seq with an H2A antibody. As the authors had technical problems with available H2A antibodies, this experiment was not included in this study. As suggested by the reviewer they performed H3-ChIP-seq for normalization of H2A.Z.
- Finally the authors included new datasets for the OsChz1 ChIP-seq analysis including a complemented line OsChz1-MYC and the knock-out oschz1-1 mutant to eliminate background signals.

While most of the new data are convincing, I just have a concern with the discrepancy between the new H3-ChIP data in WT and the oschz1-1 mutant (Figure 7) and the MNase data (Figure 6) as they should in principle reveal the same phenomenon: reduced nucleosomal occupancy in the mutant. While there is a little less H3-occupancy in the 3' region of genes in the oschz1-1 mutant, no major difference can be found at the TSS in Figure 7G compared to Figure 6d.
How do the authors explain this? Do they envisage remaining tetrasomes revealed by the H3-ChIP that would allow digestion of DNA by MNase?
Response: MNase-seq analyzed single nucleosome size DNA fragments ranging from 100 to 200 bp (Fig 6c), whereas H3 ChIP-seq analyzed DNA fragments ranging in size from 100 to 500 bp. The lower resolution of ChIP-seq likely explains the weak reduction of H3-occupancy observed in the *oschz1-1* mutant. It is generally known that ChIP-seq poorly resolves neighbouring nucleosomes whereas MNase-seq has higher resolution in assessing the presence or absence of a nucleosome (Lai and

Pugh, 2017, Nat Rev Mol Cell Biol 18: 548-562). The idea of ‘remaining tetrasomes’ may also explain/contribute to the weak reduction of H3-occupancy observed in the *oschz1-1* mutant. However, such subnucleosomal structures are generally low in eukaryotic genomes and their detection needs specific techniques such as ChIP-exonuclease analysis (Rhee et al., 2014, Cell 159: 1377-1388). Future studies may be undertaken to investigate whether or not a lack of OsChz1 causes non-canonical nucleosomes (including tetrasomes).

Minor comments:

The authors have not really responded to the question whether in their dataset (Figure 7) there are also regions that show an increase in H2A.Z occupancy?
Response: Sorry for the missed response information. Yes, increased H2A.Z could be detected at 1,262 genes, a number much lower than the 5,270 genes showing decreased H2A.Z. The increases were mainly at regions containing low levels of H2A.Z in WT; thus we believe that independent experimental approaches will be necessary to verify that they are bona fide increases but not as caused by background noises.

line 188: The absence of OsChz1 preference for H2A.Z or even a significant preference of OsChz1-C for H2A is in contrast to
should it not read ... a significant preference of OsChz1-C for H2A.Z is in ...
Response: Sorry for this complicated sentence. We have now revised the sentence to: “The absence of OsChz1 preference for H2A.Z is in contrast to the preference of yChz1 for H2A.Z reported previously”.

line 392 and 393: trends = tends
Response: Corrected

nucleosome down occupancy (several times in the text) should be replaced by reduced nucleosome occupancy
Response: Done

line 470: H2A.X marks " responsive DNA-damage sites in euchromatin". Maybe

better: that can be phosphorylated upon DNA-damage.
H2A.X marks DNA-damage sites in its phosphorylated form.
Response: Changed

Reviewer #2 (Remarks to the Author):

The revised manuscript has addressed most of my concerns on the structural parts.

It is worth noting that the CHZ domain-containing proteins may present less or no preference for H2A.Z-H2B dimer, as demonstrated by OsChz1 in this study and AtChz1b in the previous study (Wang et al.). The high eukaryotes CHZ domain-containing proteins have been considered functionally related to yeast Chz1 until Wang et al. reveal the molecular basis for the preferential recognition of H2A.Z-H2B dimer by Chz1. In fact, most CHZ domain-containing proteins lack the key residues that are selectively interacting with H2A.Z. From my point of view, the CHZ domain-containing proteins do not necessarily show specificity to H2A.Z. It should be reasonable if these proteins have distinct histone dimer binding preferences.

The authors may need to consider to tune down the conclusion regarding the OsChz1 binding preference for H2A over H2A.Z. First, OsChz1-C presents a limited preference for H2A over H2A.Z, as suggested by the pull-down and the ITC experiments. Second, although the crystal structure revealed some interactions between OsChz1 and H2A-H2B dimer, it lacks H2A specific residues involving the preferential binding of OsChz1-C.
Response: Agree, we have carefully checked through our manuscript and made necessary changes to avoid any overstatement about the OsChz1 binding preference for H2A over H2A.Z.

Minor points
The residues numbers of aligned sequences, as least for OsChz1, should be given in the FigS3 and FigS5.
Response: Done

Reviewer #1 (Remarks to the Author):

The authors have replied to all my comments in a satisfactory manner. In my opinion, the manuscript can now be published in its current form.

Reviewer #2 (Remarks to the Author):

The authors have addressed all of my concerns satisfactorily. I recommend its publication.
